# Supplementary figures and images for: Differential expression of lysine acetylation proteins in gastric cancer treated with a new antitumor agent bioactive peptide chelate selenium
Source: PeerJ. 2023 Jan 17;11:e14384. doi: 10.7717/peerj.14384 (PMC9854375; doi:10.7717/peerj.14384)

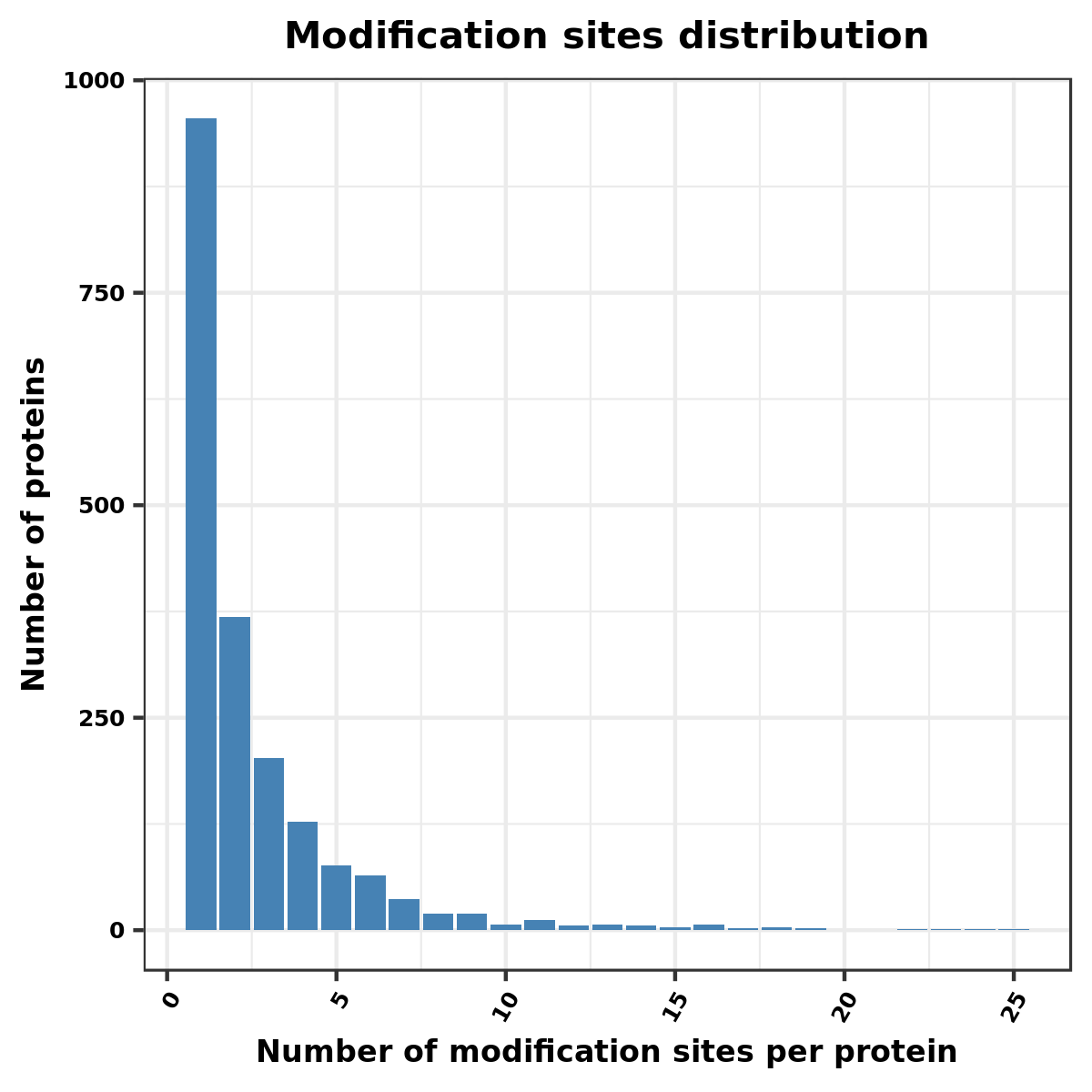

Supplement: Supplemental Information 8 [file peerj-11-14384-s008.png]

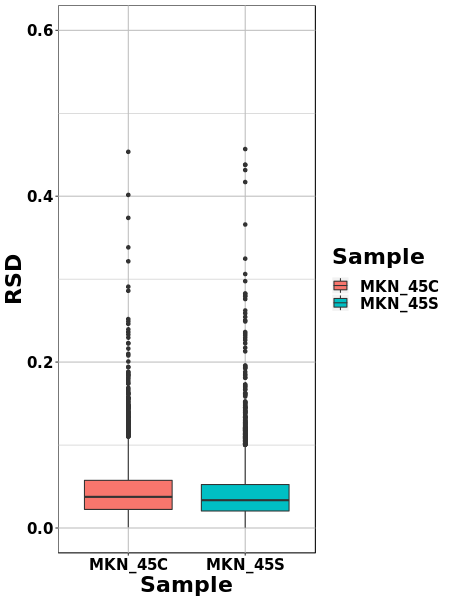

Supplement: Supplemental Information 9 [file peerj-11-14384-s009.png]

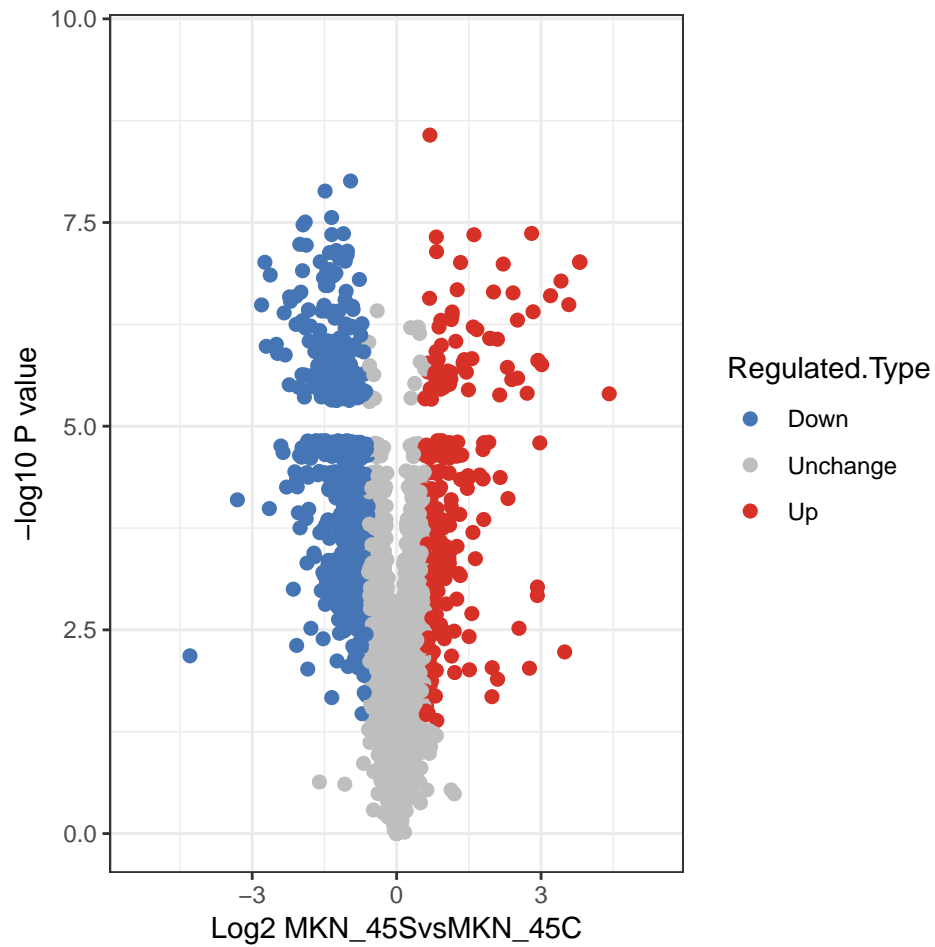

Supplement: Data S1 [file peerj-11-14384-s010.zip › Raw Data/KA076TPAc_FC1.5_update_clean/3-Differentially_expressed_protein/MKN_45SvsMKN_45C-volcano_plot.pdf]

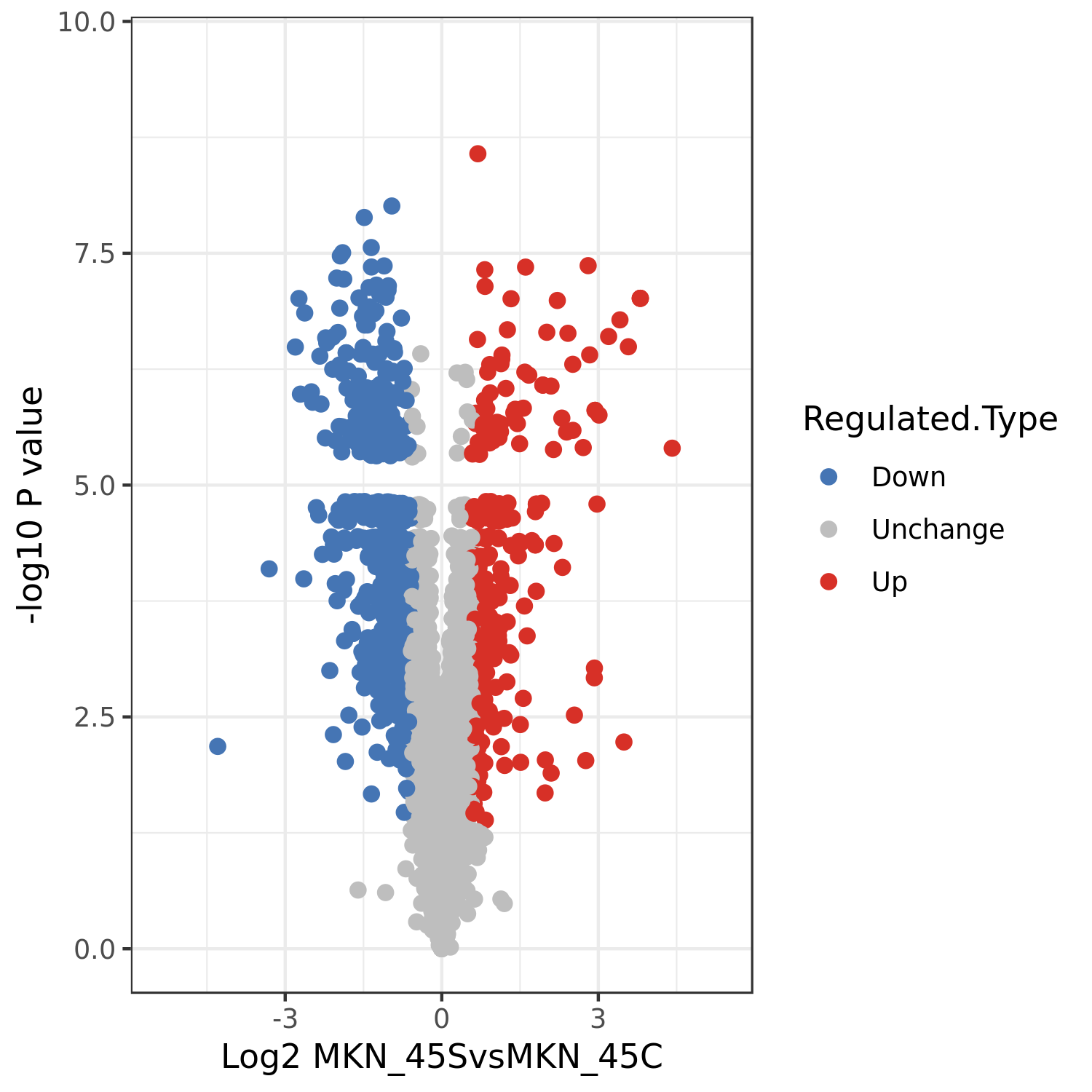

Supplement: Data S1 [file peerj-11-14384-s010.zip › Raw Data/KA076TPAc_FC1.5_update_clean/3-Differentially_expressed_protein/MKN_45SvsMKN_45C-volcano_plot.png]

Regulation   ■ Up   ■ Down

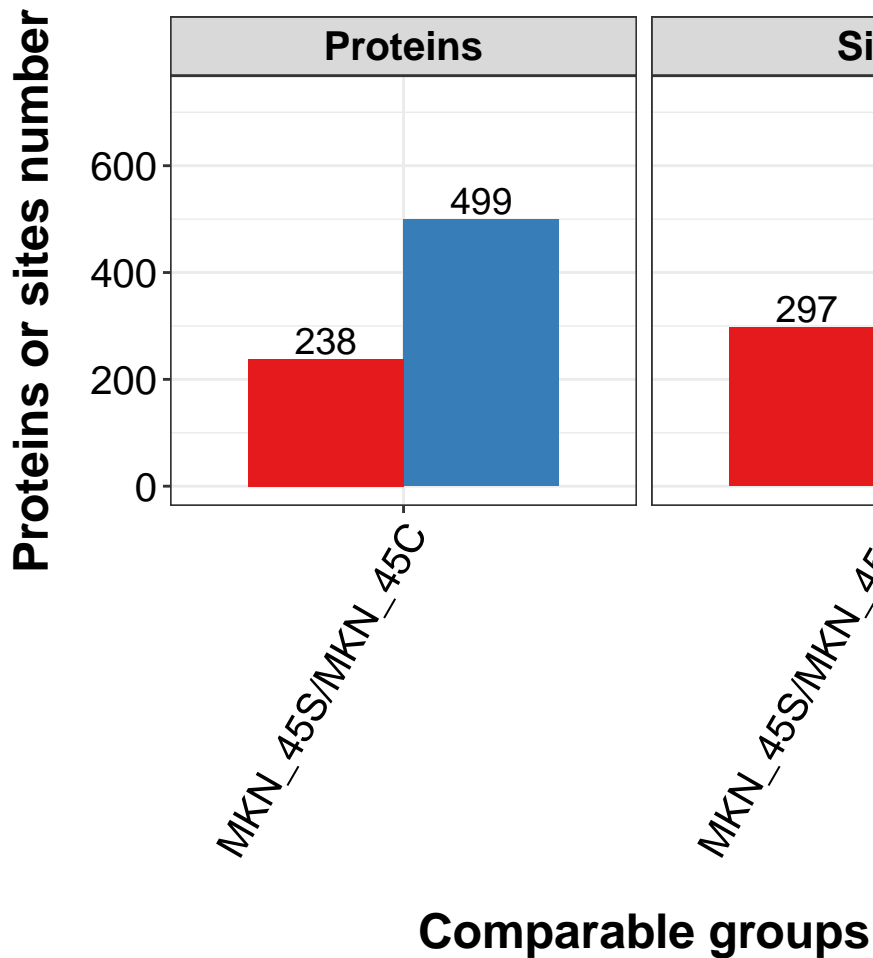

Supplement: Data S1 [file peerj-11-14384-s010.zip › Raw Data/KA076TPAc_FC1.5_update_clean/3-Differentially_expressed_protein/Statistics.pdf]

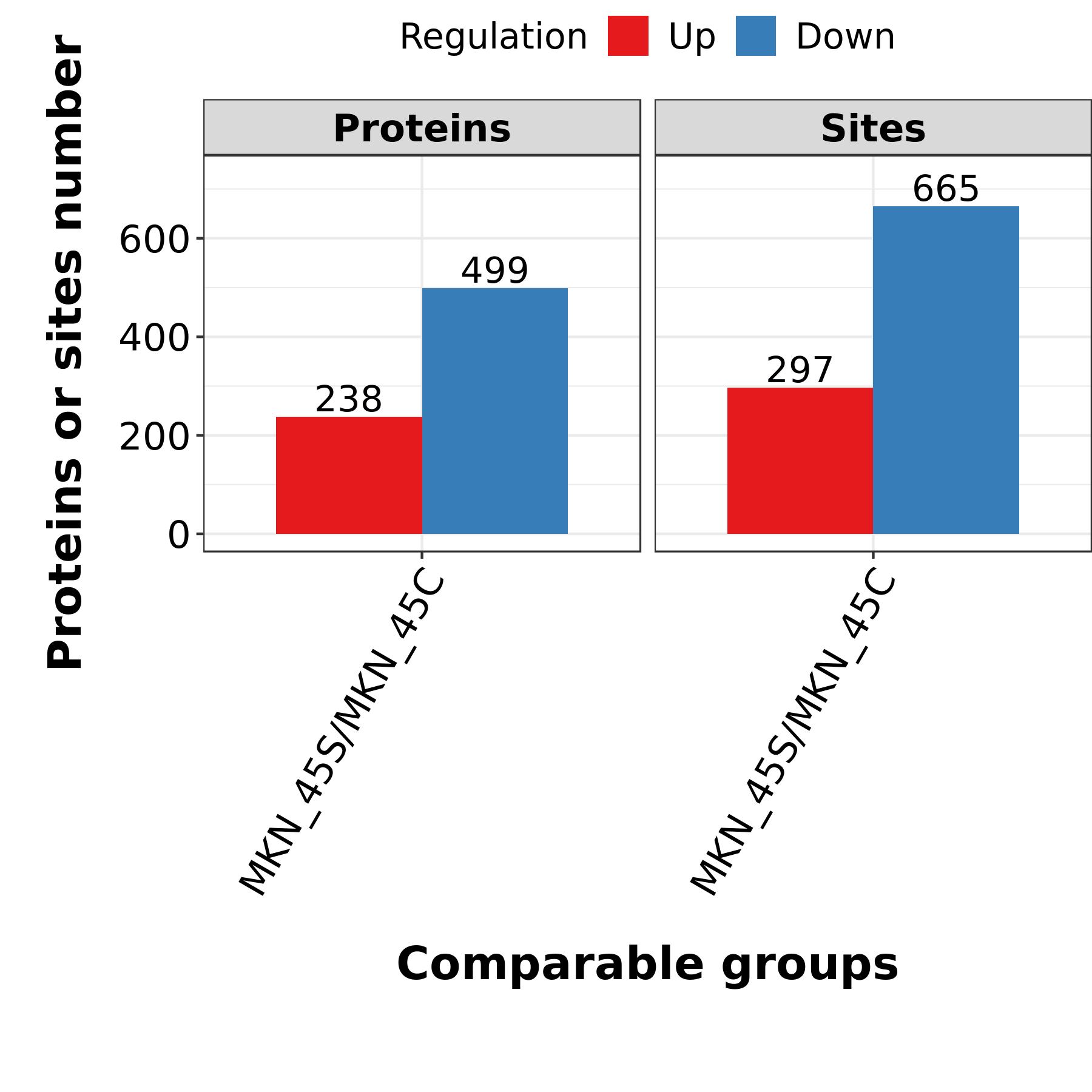

Supplement: Data S1 [file peerj-11-14384-s010.zip › Raw Data/KA076TPAc_FC1.5_update_clean/3-Differentially_expressed_protein/Statistics.png]

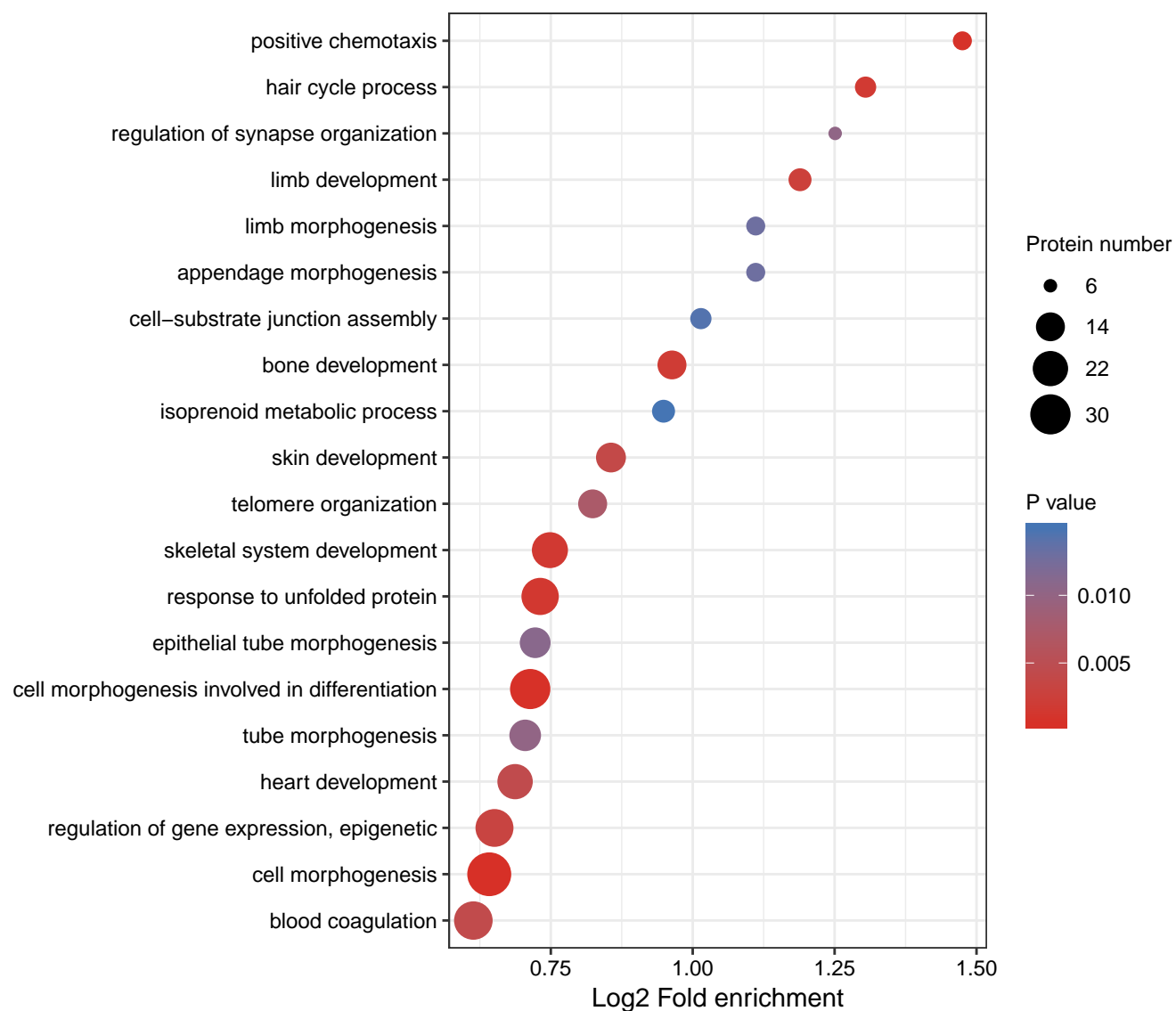

Supplement: Data S1 [file peerj-11-14384-s010.zip › Raw Data/KA076TPAc_FC1.5_update_clean/6-Functional_enrichment/MKN_45SvsMKN_45C/all-BP.pdf]

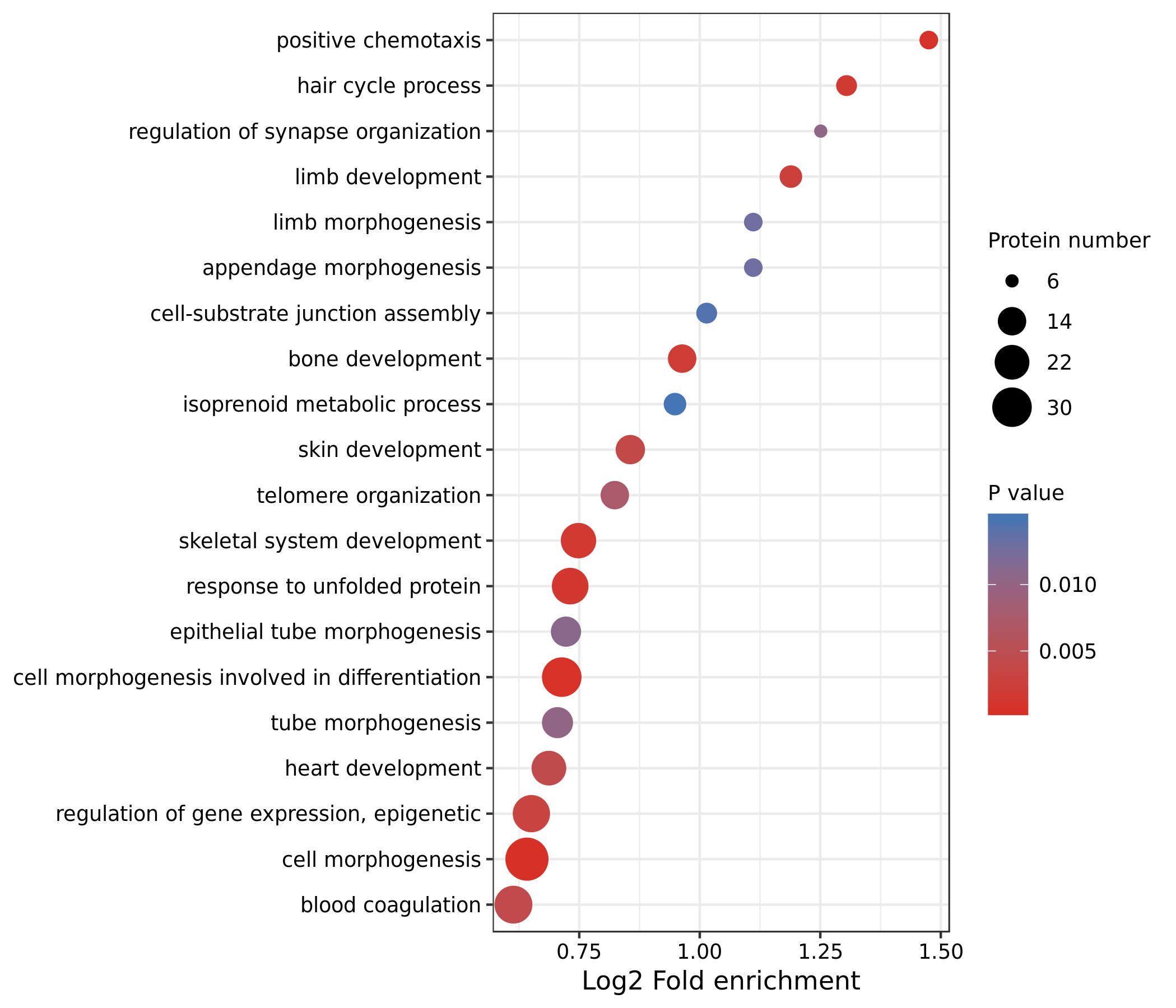

Supplement: Data S1 [file peerj-11-14384-s010.zip › Raw Data/KA076TPAc_FC1.5_update_clean/6-Functional_enrichment/MKN_45SvsMKN_45C/all-BP.png]

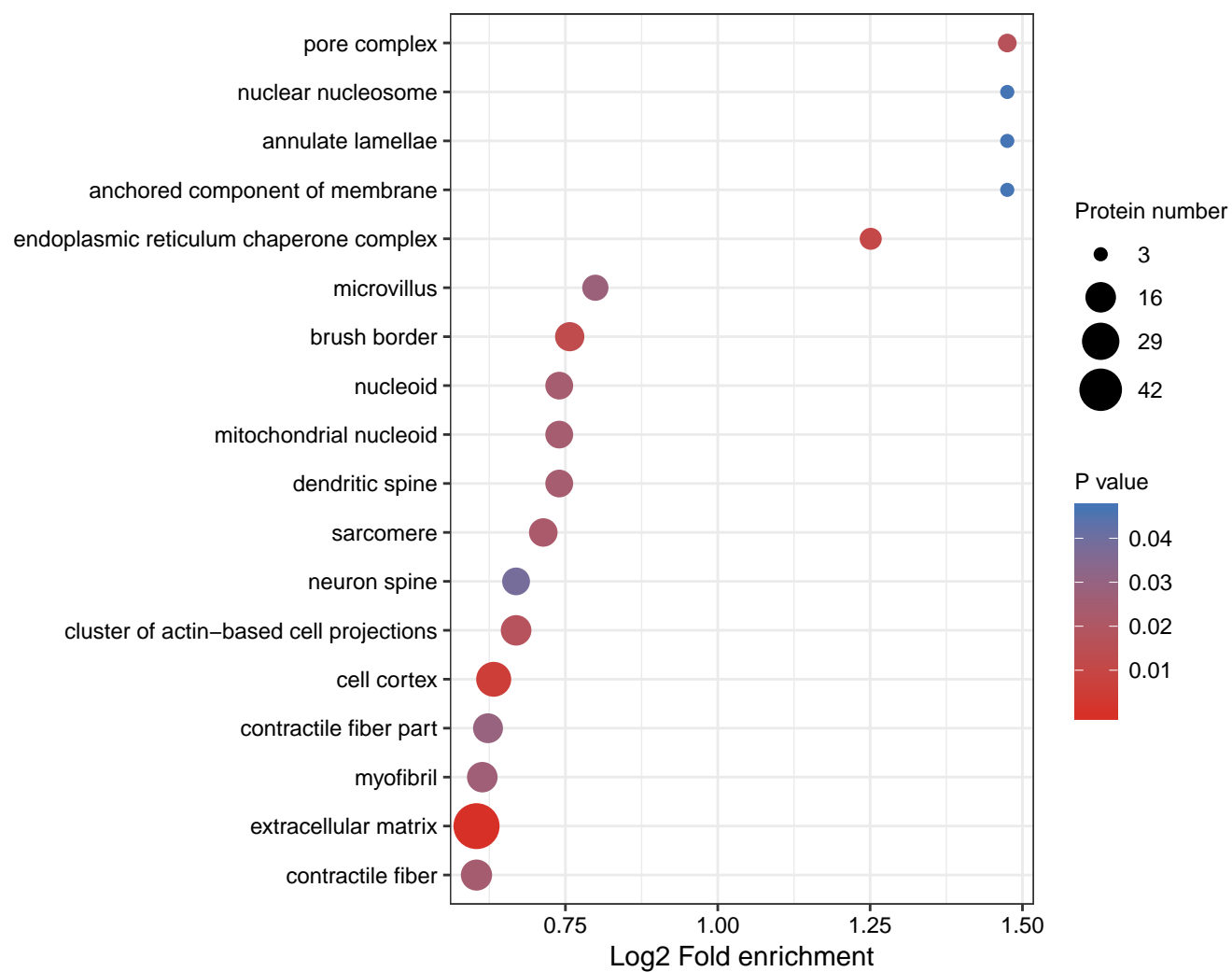

Supplement: Data S1 [file peerj-11-14384-s010.zip › Raw Data/KA076TPAc_FC1.5_update_clean/6-Functional_enrichment/MKN_45SvsMKN_45C/all-CC.pdf]

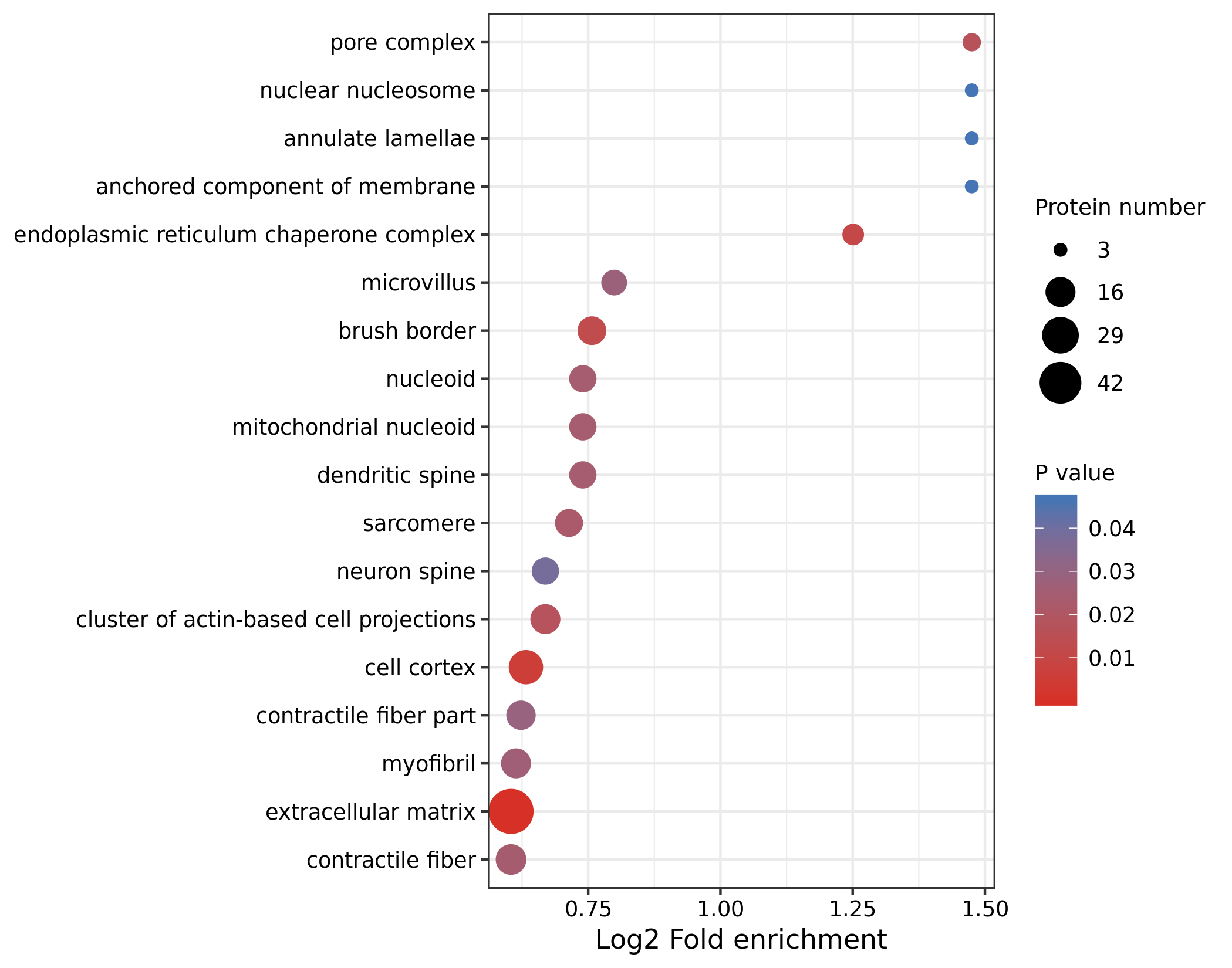

Supplement: Data S1 [file peerj-11-14384-s010.zip › Raw Data/KA076TPAc_FC1.5_update_clean/6-Functional_enrichment/MKN_45SvsMKN_45C/all-CC.png]

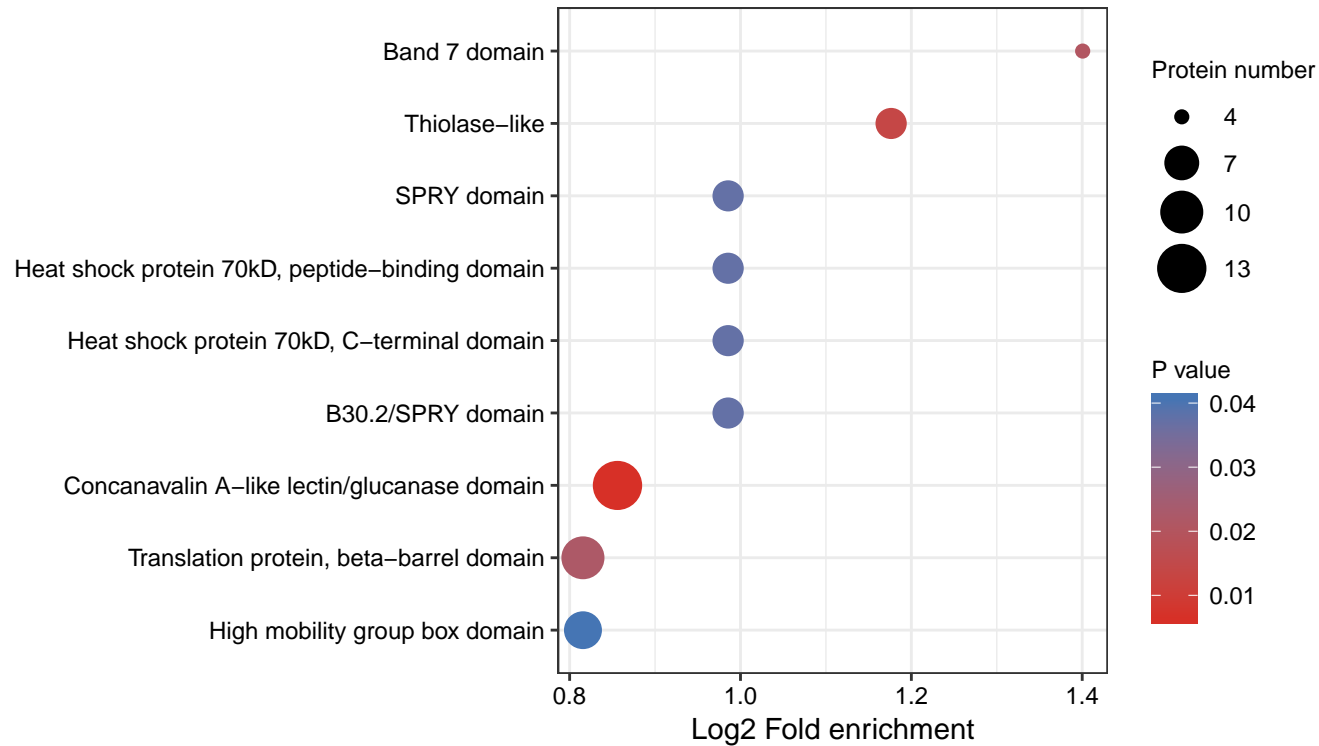

Supplement: Data S1 [file peerj-11-14384-s010.zip › Raw Data/KA076TPAc_FC1.5_update_clean/6-Functional_enrichment/MKN_45SvsMKN_45C/all-domain.pdf]

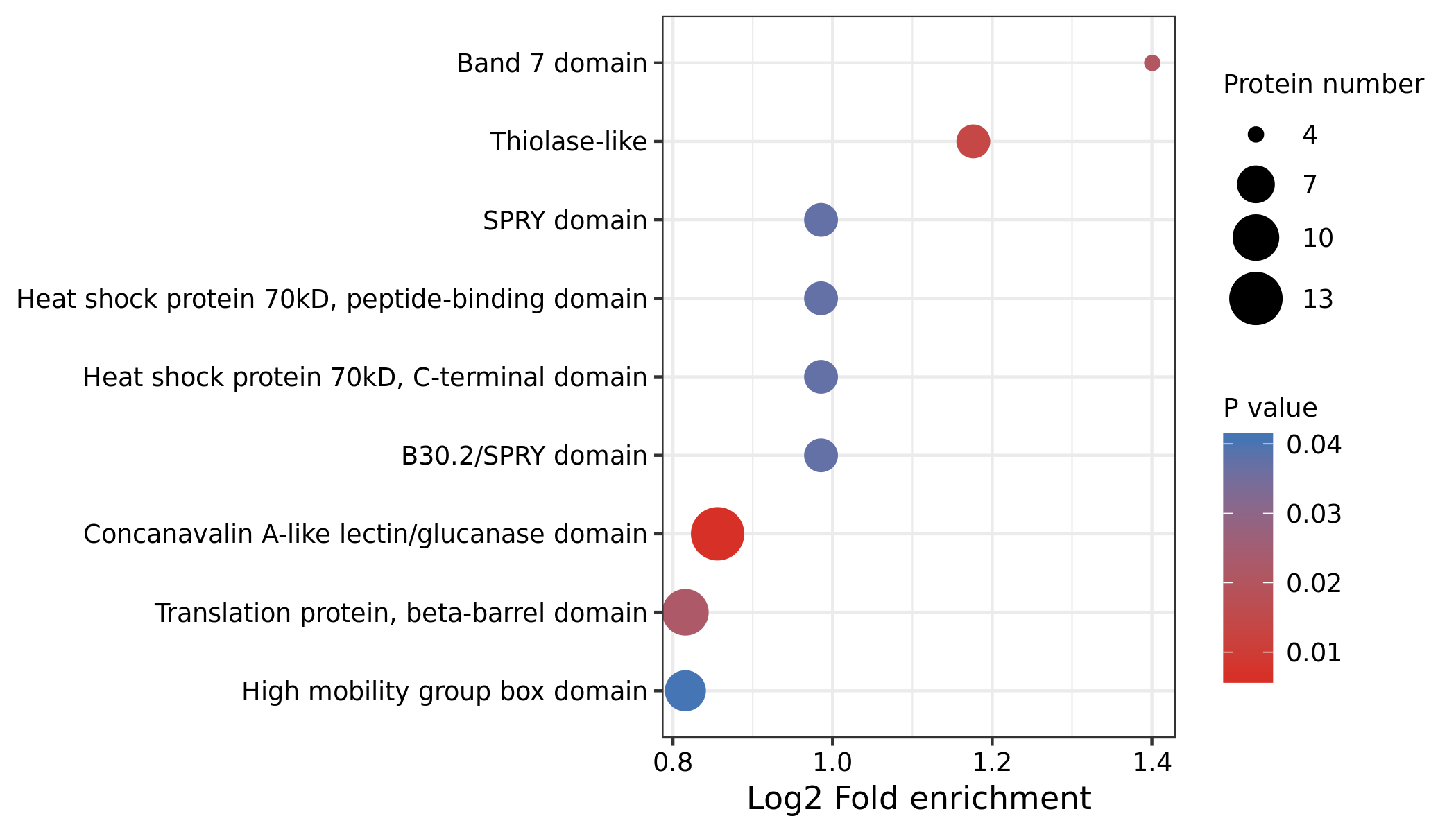

Supplement: Data S1 [file peerj-11-14384-s010.zip › Raw Data/KA076TPAc_FC1.5_update_clean/6-Functional_enrichment/MKN_45SvsMKN_45C/all-domain.png]

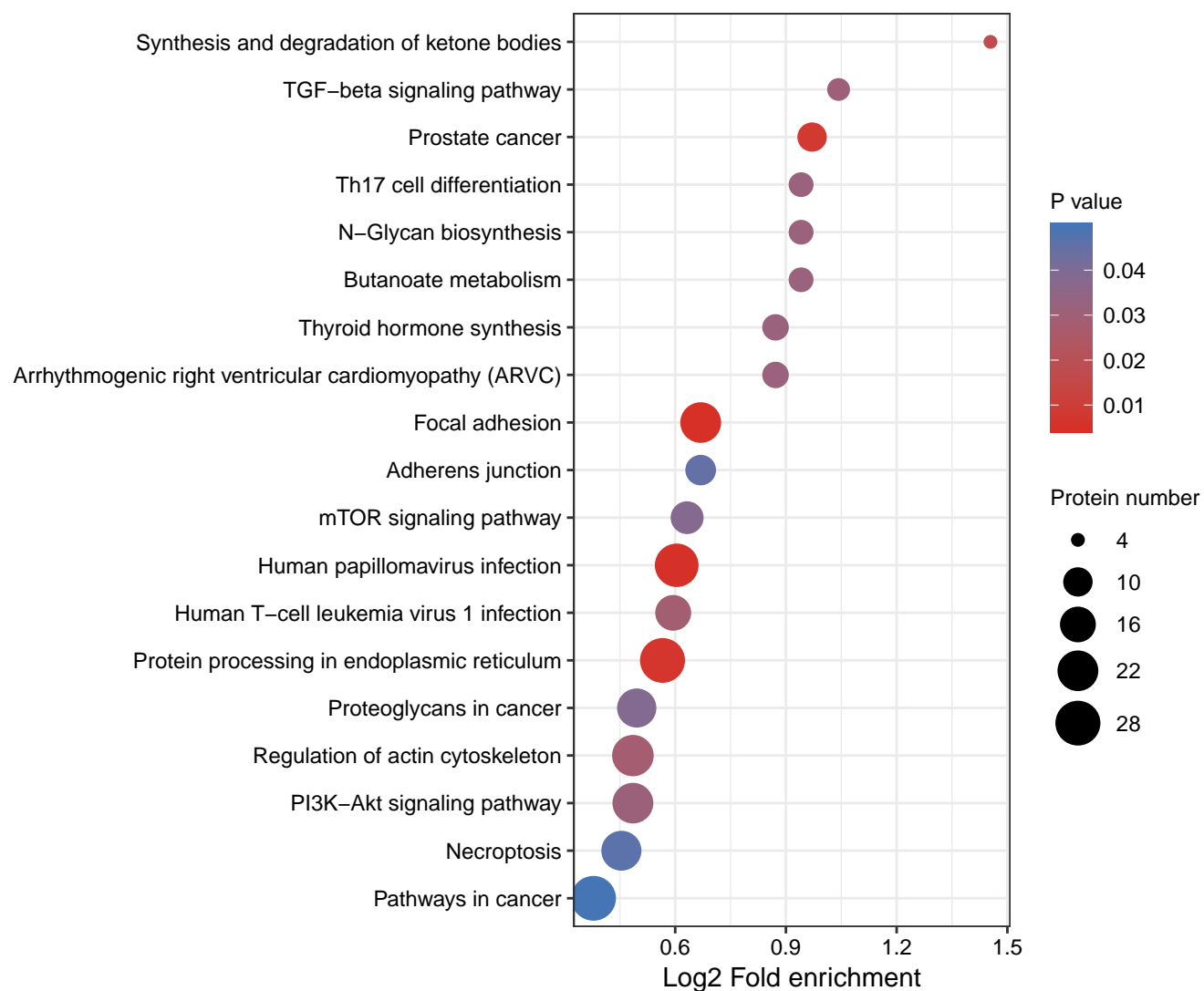

Supplement: Data S1 [file peerj-11-14384-s010.zip › Raw Data/KA076TPAc_FC1.5_update_clean/6-Functional_enrichment/MKN_45SvsMKN_45C/all-kegg.pdf]

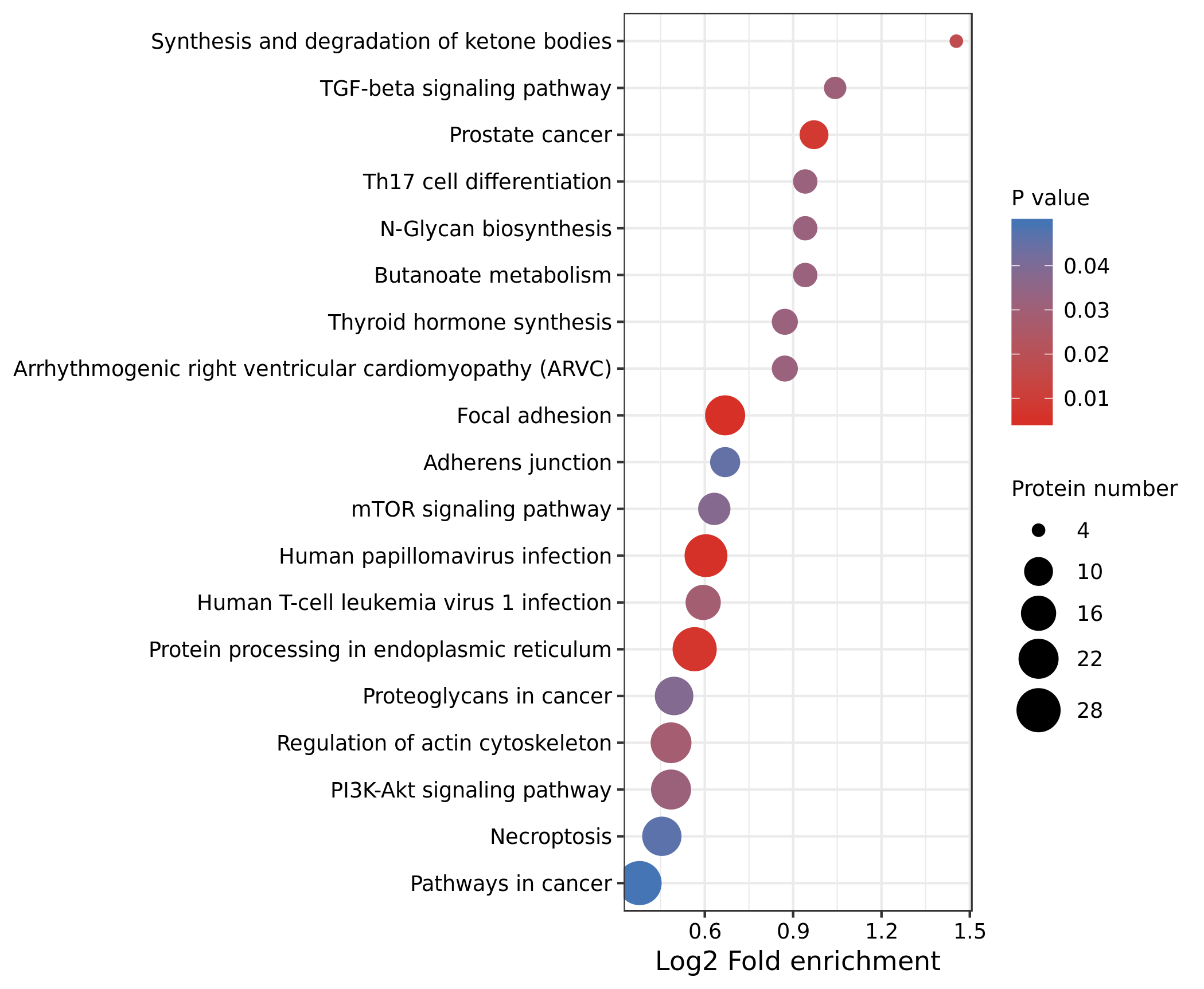

Supplement: Data S1 [file peerj-11-14384-s010.zip › Raw Data/KA076TPAc_FC1.5_update_clean/6-Functional_enrichment/MKN_45SvsMKN_45C/all-kegg.png]

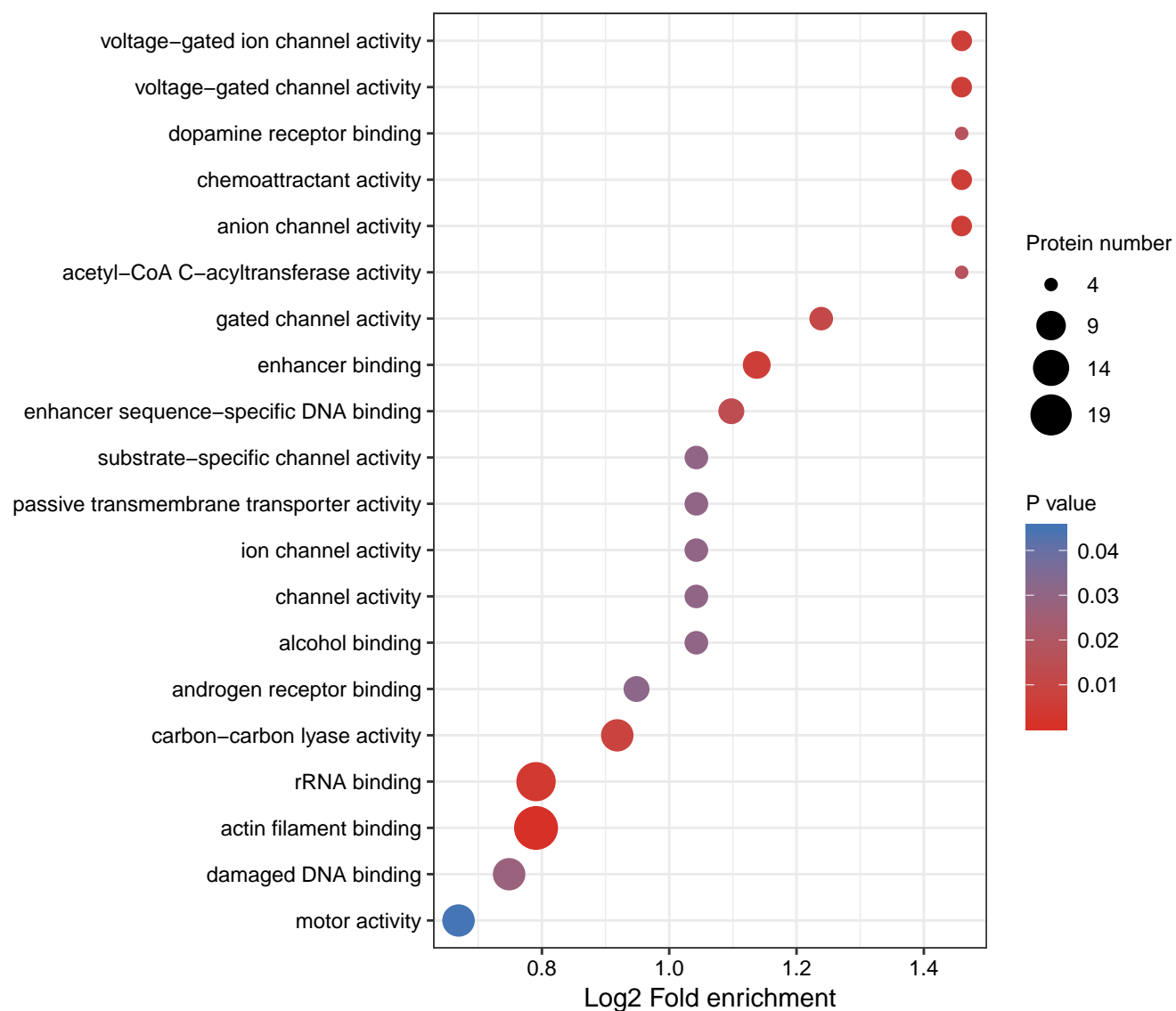

Supplement: Data S1 [file peerj-11-14384-s010.zip › Raw Data/KA076TPAc_FC1.5_update_clean/6-Functional_enrichment/MKN_45SvsMKN_45C/all-MF.pdf]

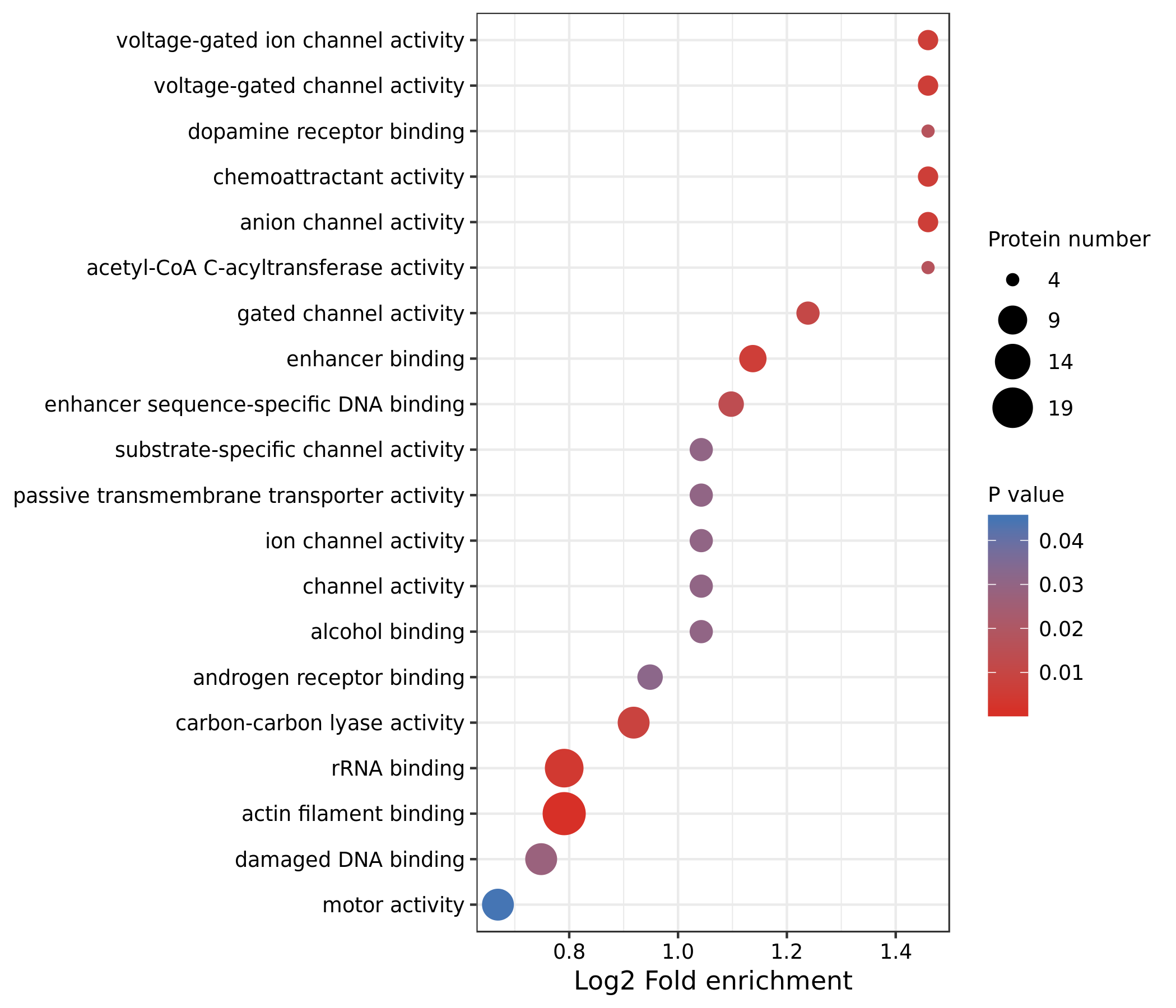

Supplement: Data S1 [file peerj-11-14384-s010.zip › Raw Data/KA076TPAc_FC1.5_update_clean/6-Functional_enrichment/MKN_45SvsMKN_45C/all-MF.png]

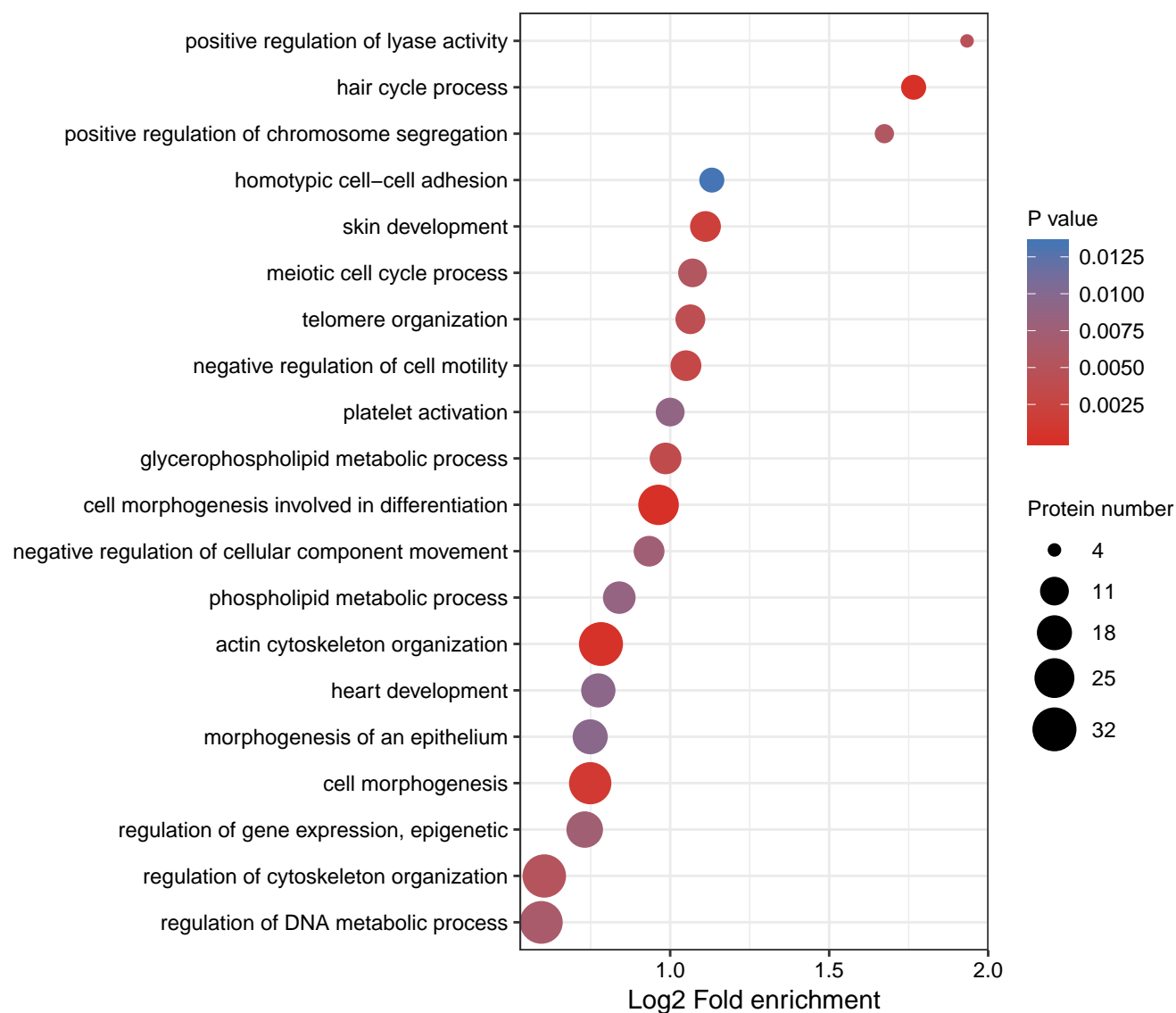

Supplement: Data S1 [file peerj-11-14384-s010.zip › Raw Data/KA076TPAc_FC1.5_update_clean/6-Functional_enrichment/MKN_45SvsMKN_45C/Down-BP.pdf]

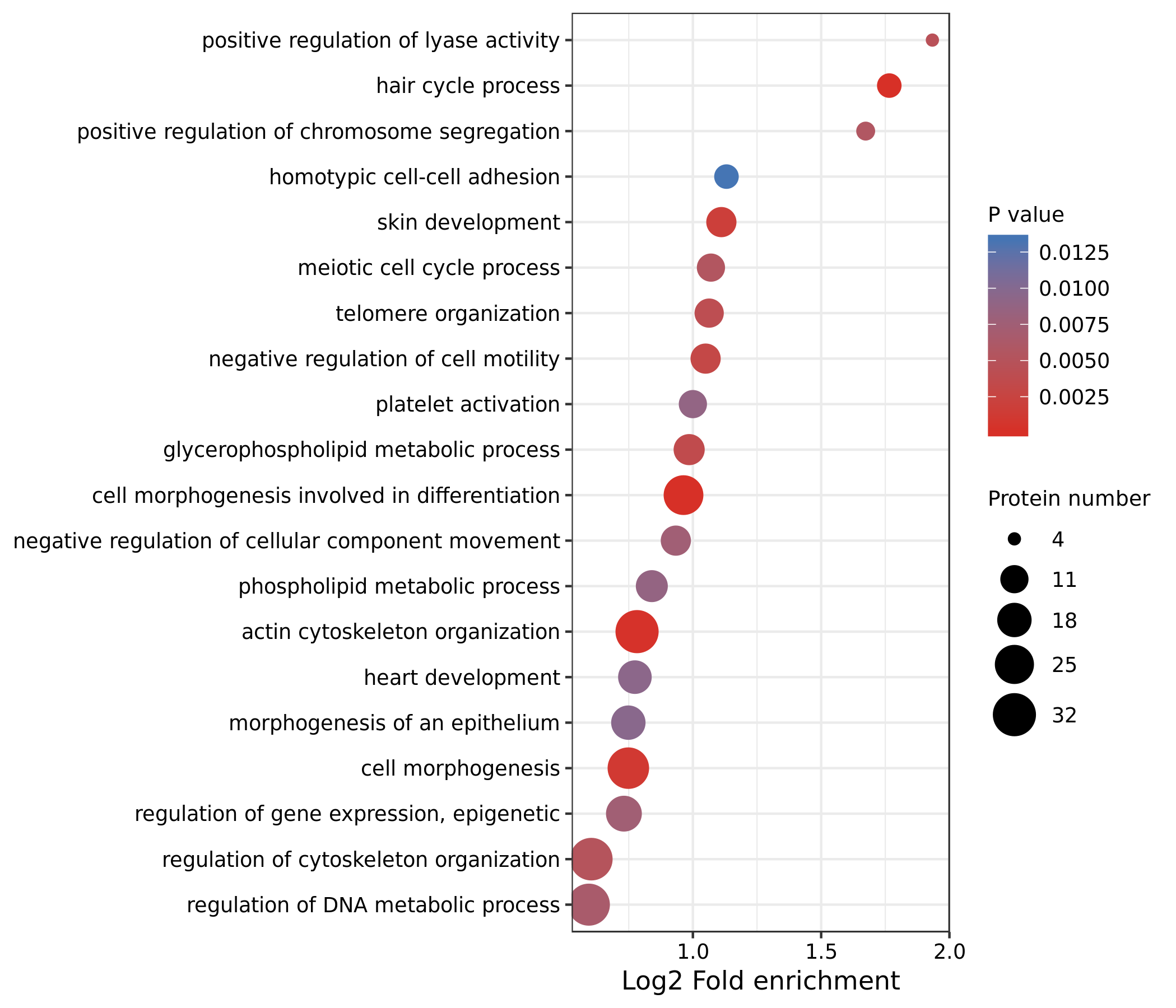

Supplement: Data S1 [file peerj-11-14384-s010.zip › Raw Data/KA076TPAc_FC1.5_update_clean/6-Functional_enrichment/MKN_45SvsMKN_45C/Down-BP.png]

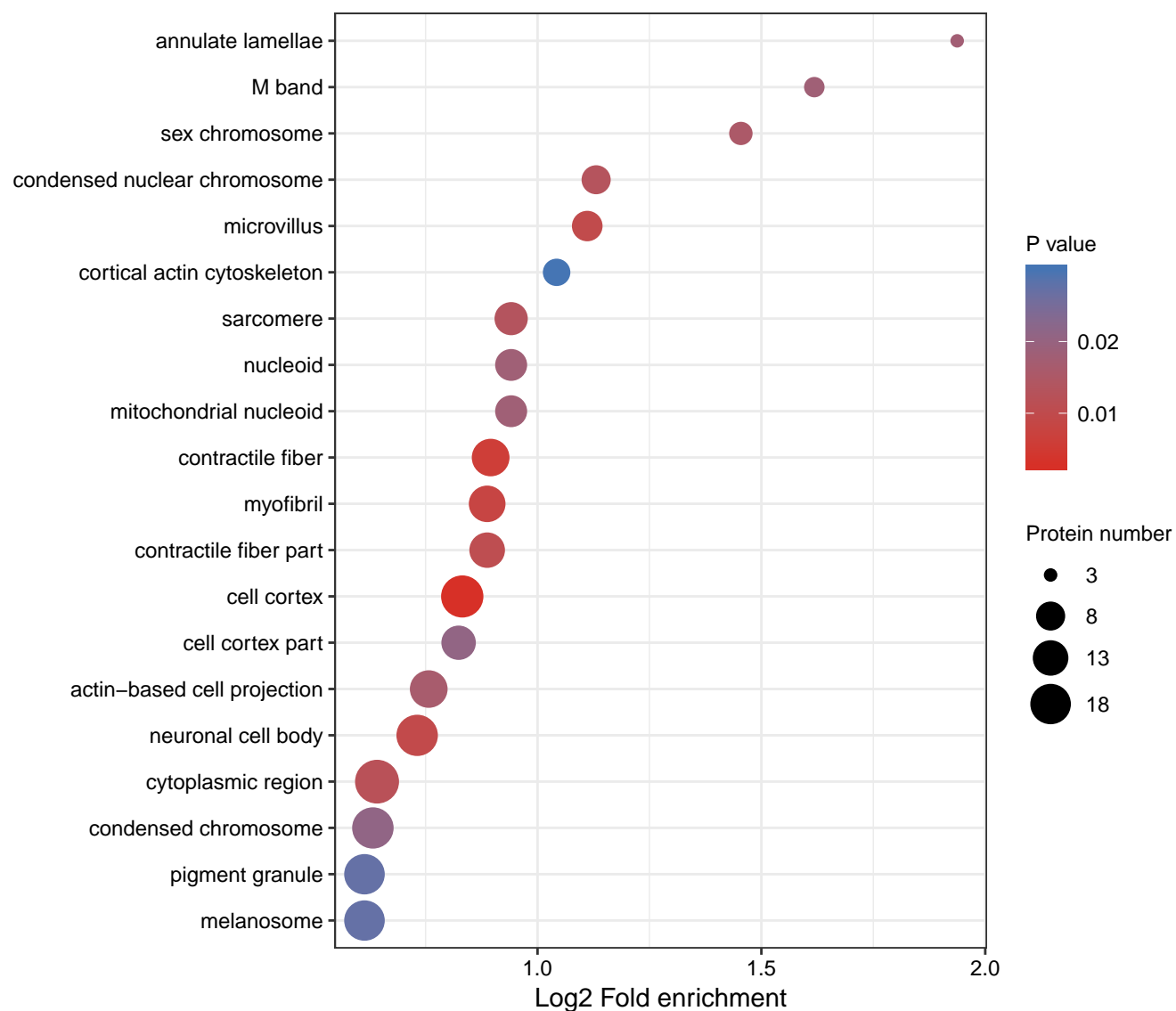

Supplement: Data S1 [file peerj-11-14384-s010.zip › Raw Data/KA076TPAc_FC1.5_update_clean/6-Functional_enrichment/MKN_45SvsMKN_45C/Down-CC.pdf]

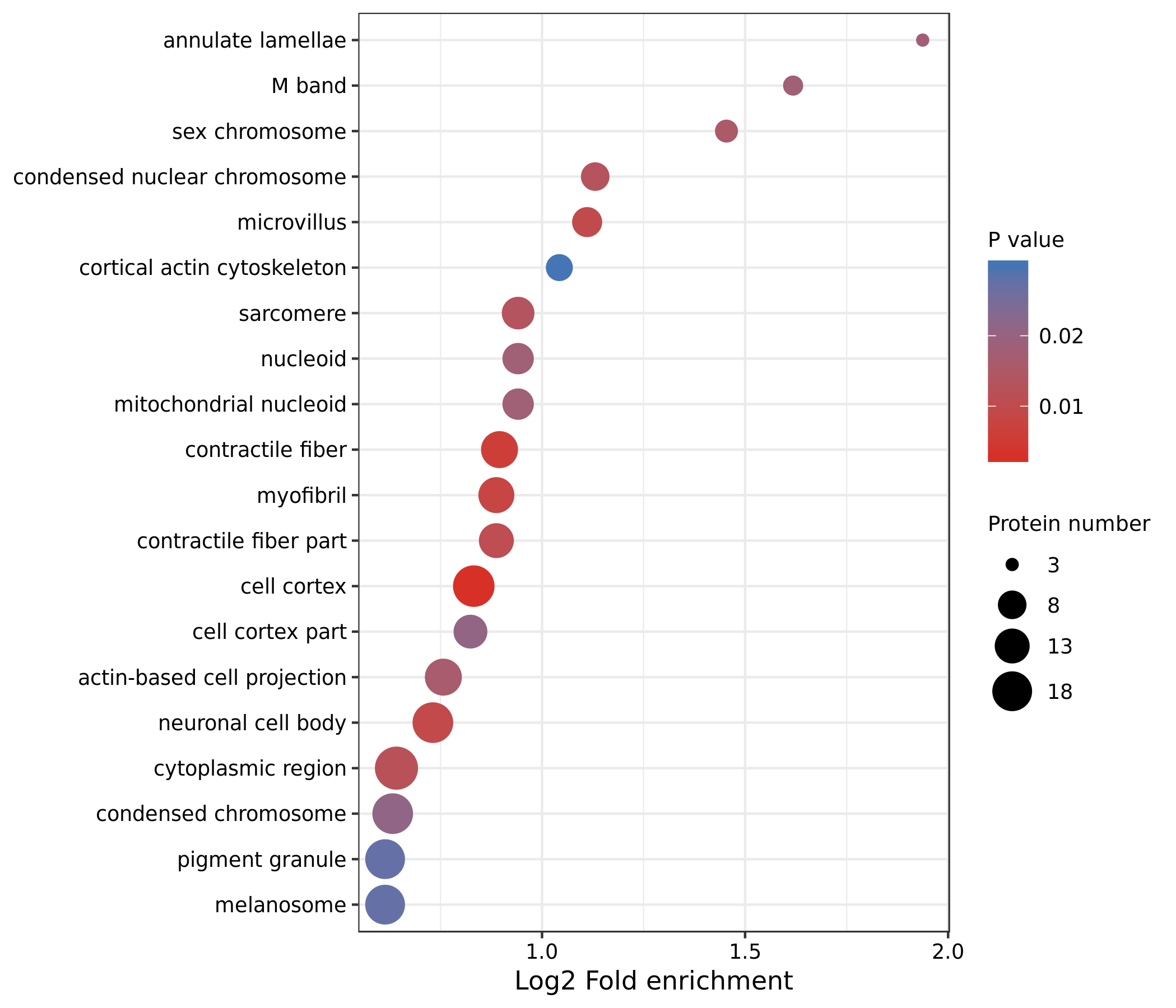

Supplement: Data S1 [file peerj-11-14384-s010.zip › Raw Data/KA076TPAc_FC1.5_update_clean/6-Functional_enrichment/MKN_45SvsMKN_45C/Down-CC.png]

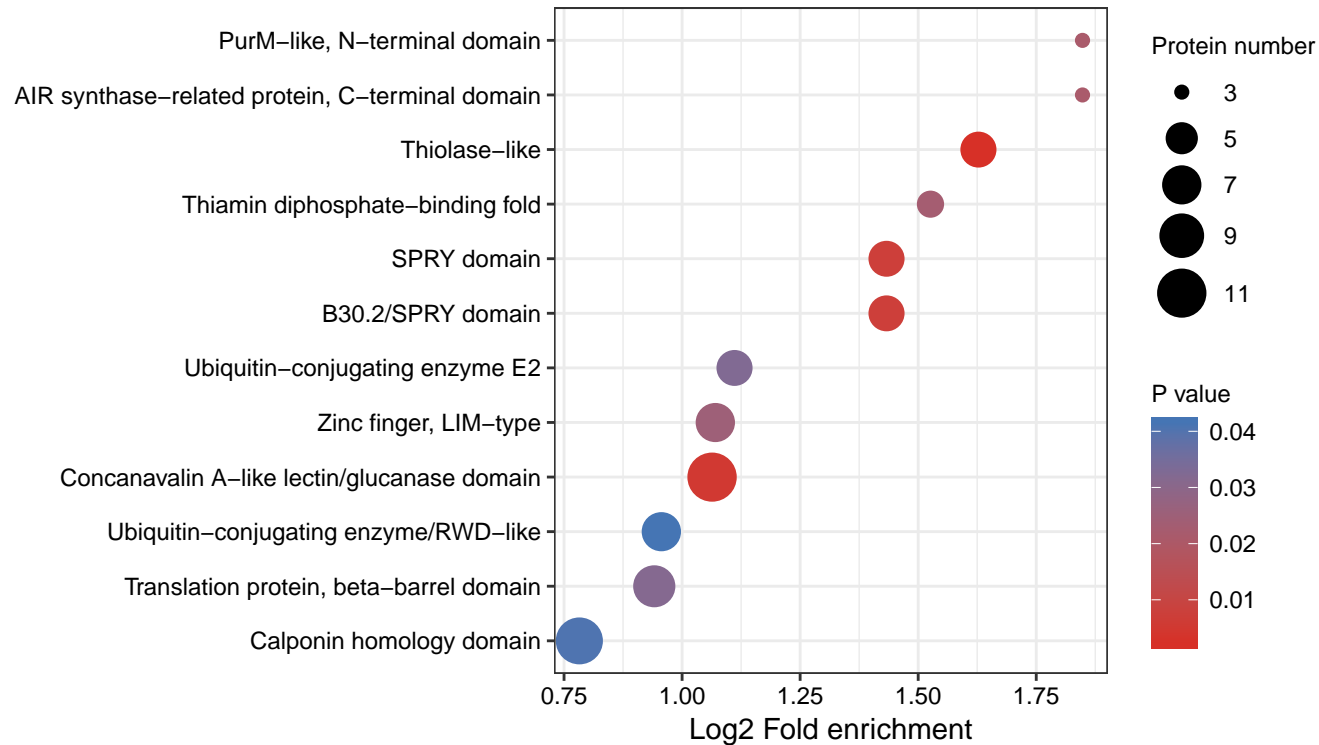

Supplement: Data S1 [file peerj-11-14384-s010.zip › Raw Data/KA076TPAc_FC1.5_update_clean/6-Functional_enrichment/MKN_45SvsMKN_45C/Down-domain.pdf]

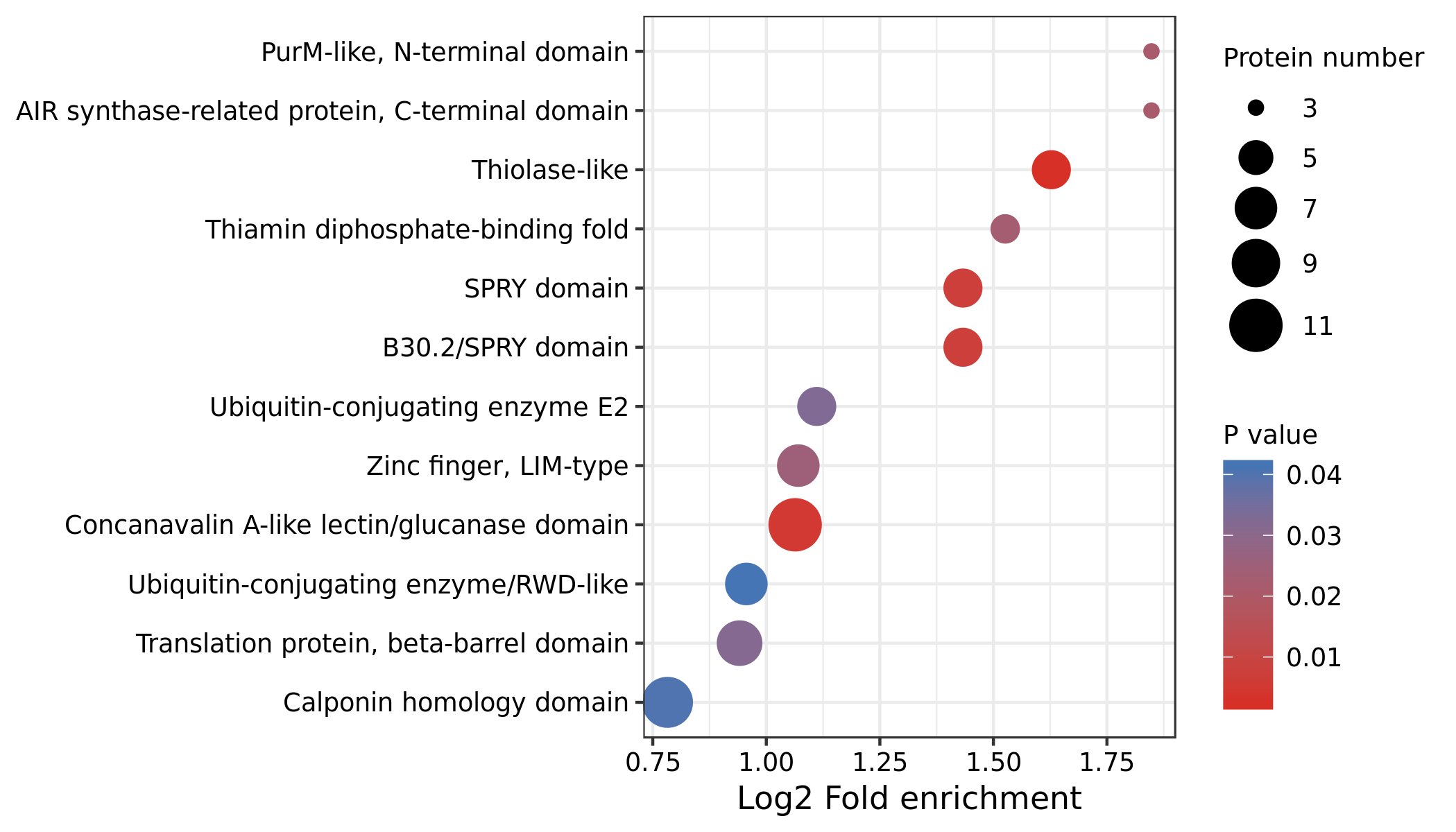

Supplement: Data S1 [file peerj-11-14384-s010.zip › Raw Data/KA076TPAc_FC1.5_update_clean/6-Functional_enrichment/MKN_45SvsMKN_45C/Down-domain.png]

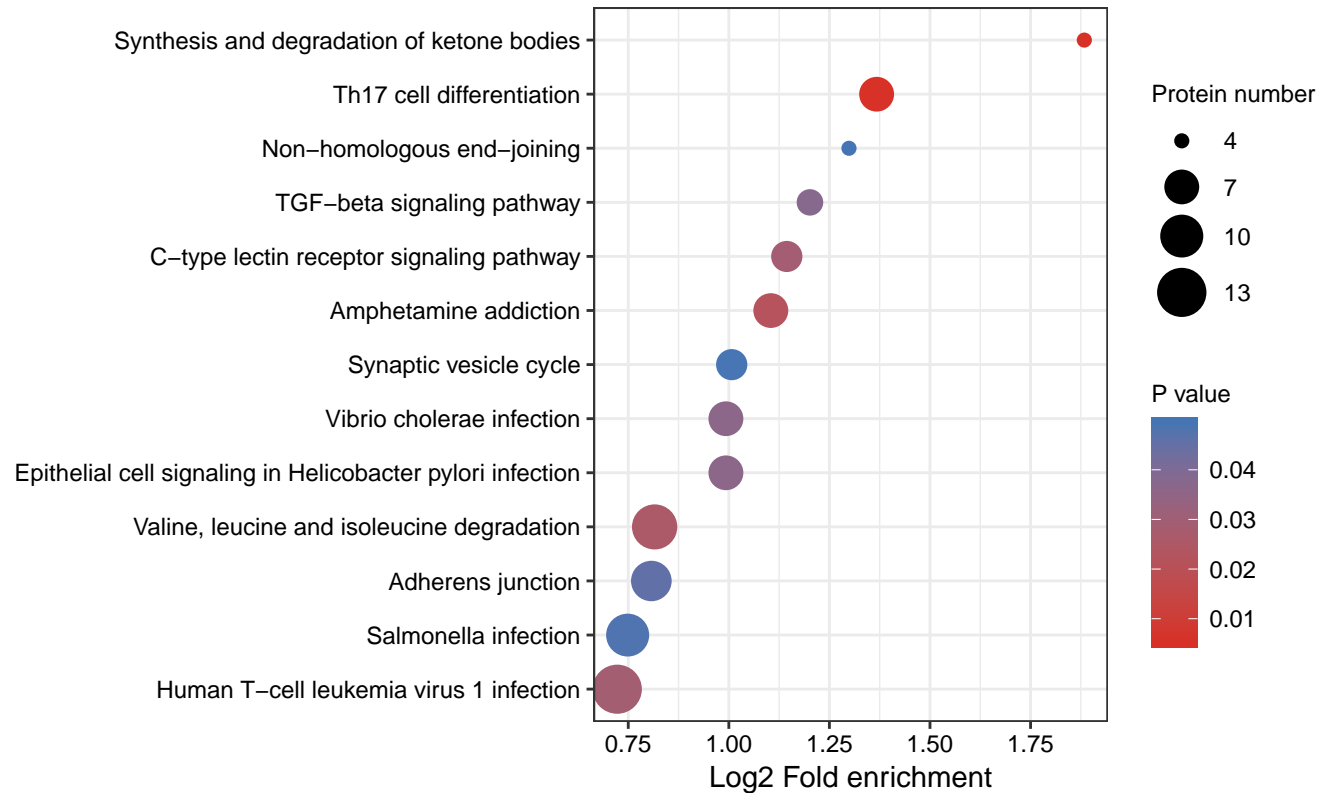

Supplement: Data S1 [file peerj-11-14384-s010.zip › Raw Data/KA076TPAc_FC1.5_update_clean/6-Functional_enrichment/MKN_45SvsMKN_45C/Down-kegg.pdf]

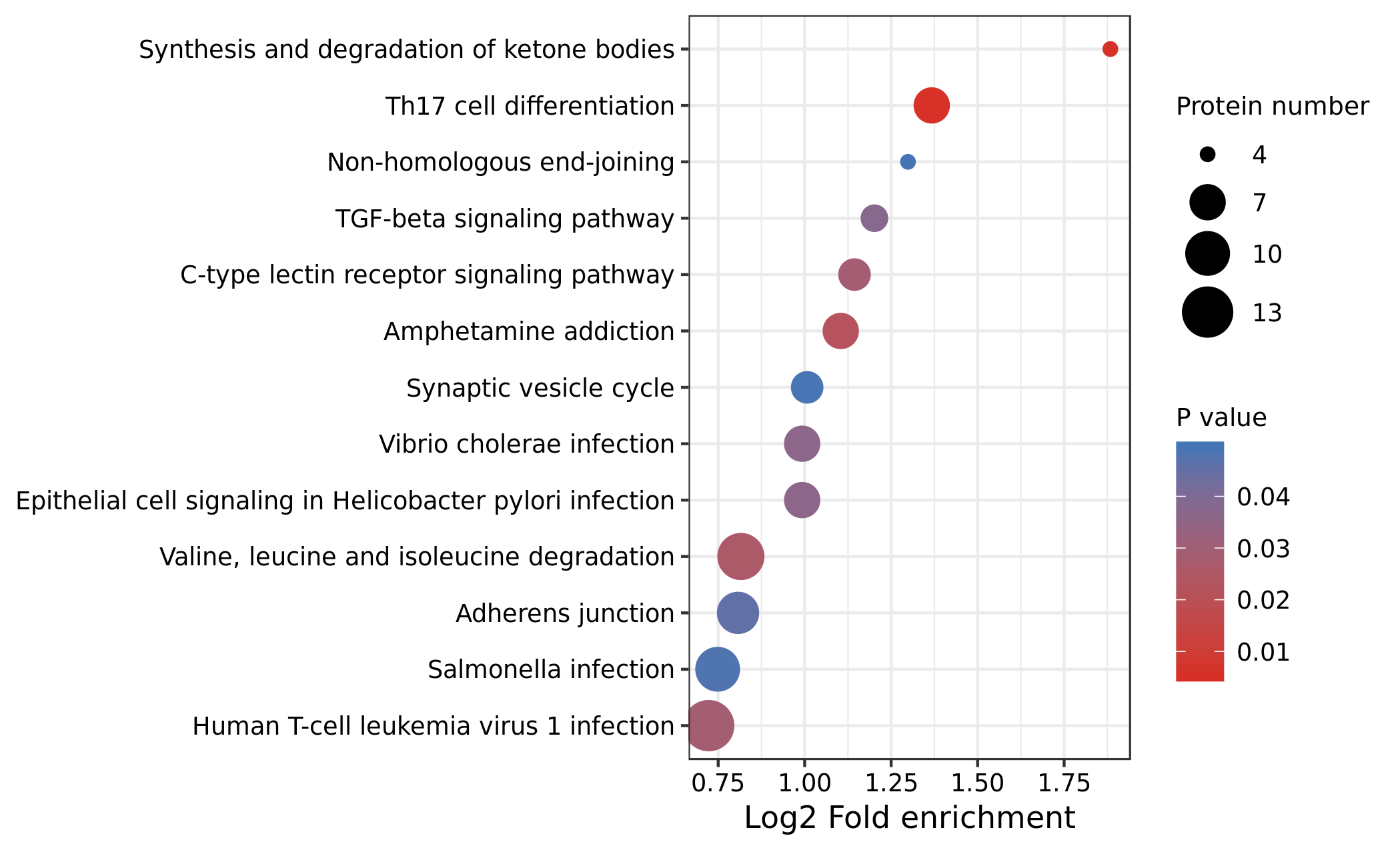

Supplement: Data S1 [file peerj-11-14384-s010.zip › Raw Data/KA076TPAc_FC1.5_update_clean/6-Functional_enrichment/MKN_45SvsMKN_45C/Down-kegg.png]

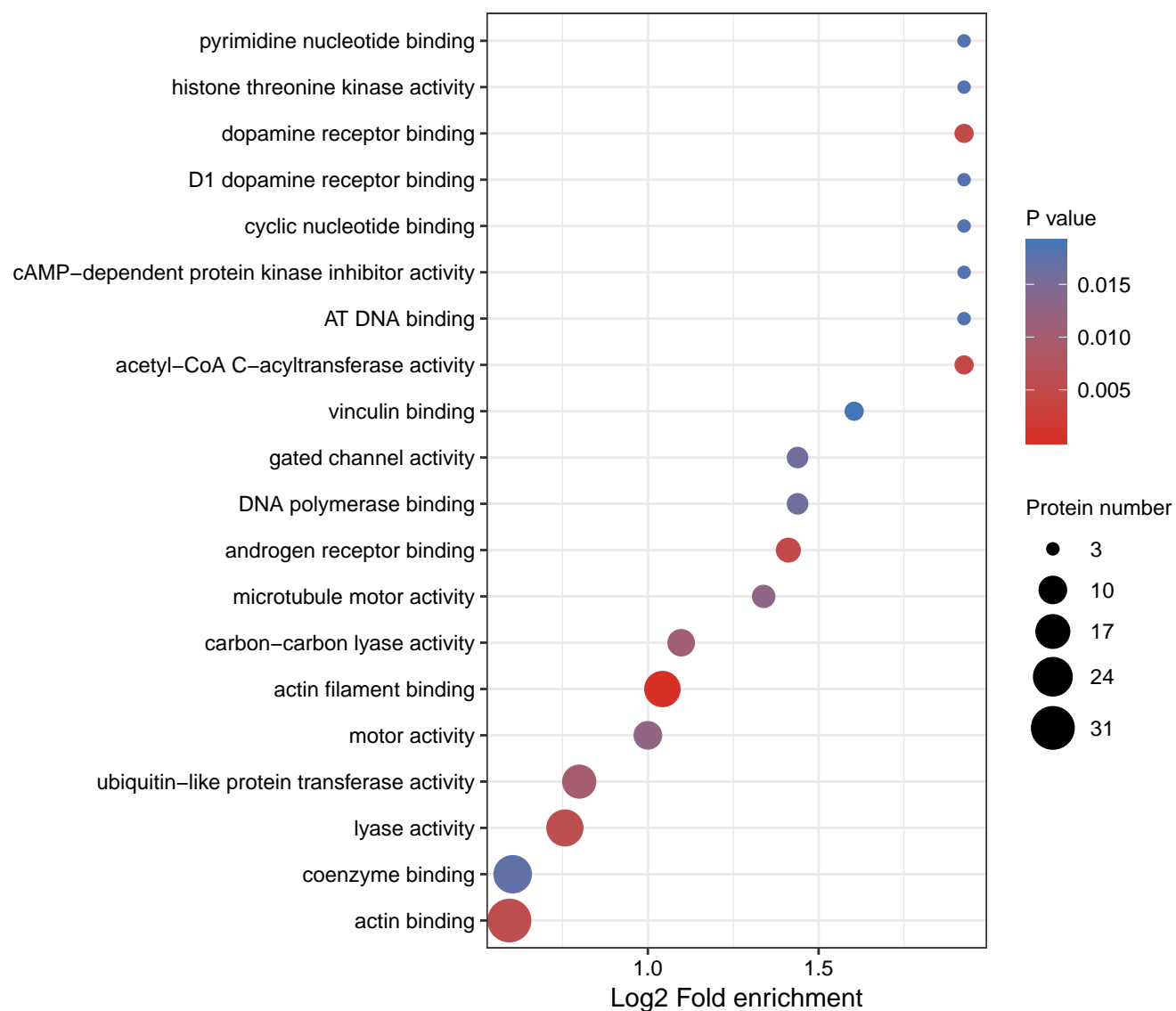

Supplement: Data S1 [file peerj-11-14384-s010.zip › Raw Data/KA076TPAc_FC1.5_update_clean/6-Functional_enrichment/MKN_45SvsMKN_45C/Down-MF.pdf]

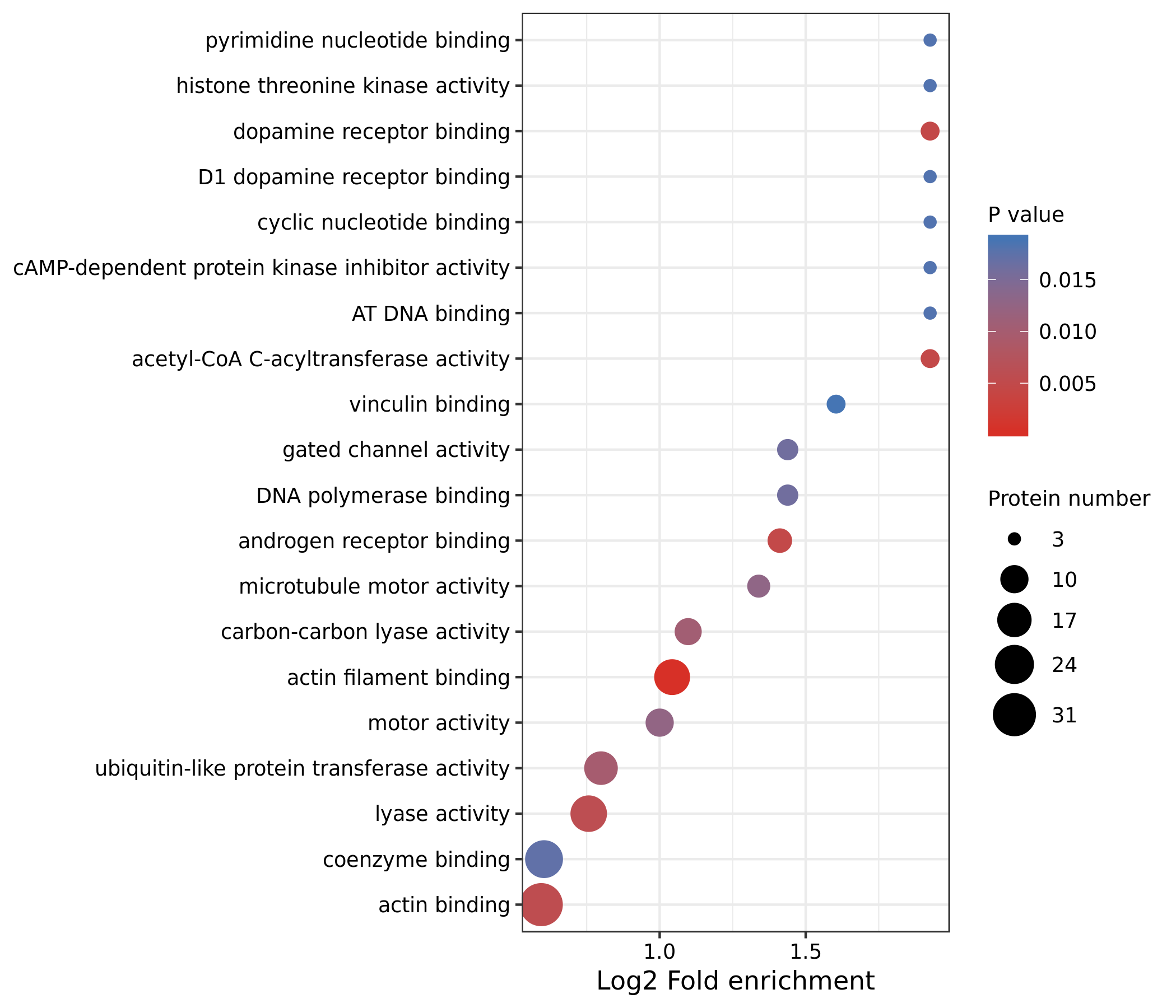

Supplement: Data S1 [file peerj-11-14384-s010.zip › Raw Data/KA076TPAc_FC1.5_update_clean/6-Functional_enrichment/MKN_45SvsMKN_45C/Down-MF.png]

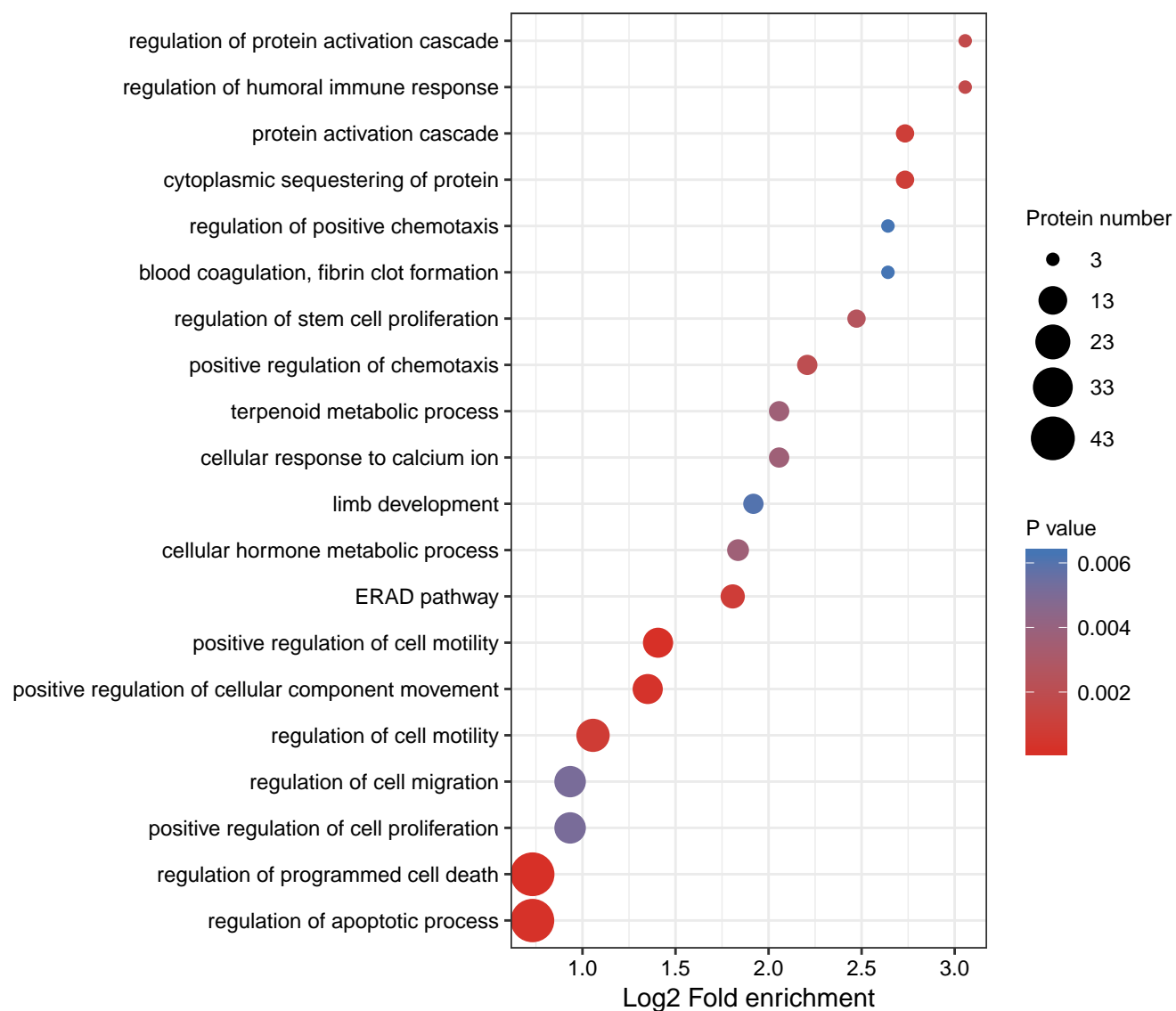

Supplement: Data S1 [file peerj-11-14384-s010.zip › Raw Data/KA076TPAc_FC1.5_update_clean/6-Functional_enrichment/MKN_45SvsMKN_45C/Up-BP.pdf]

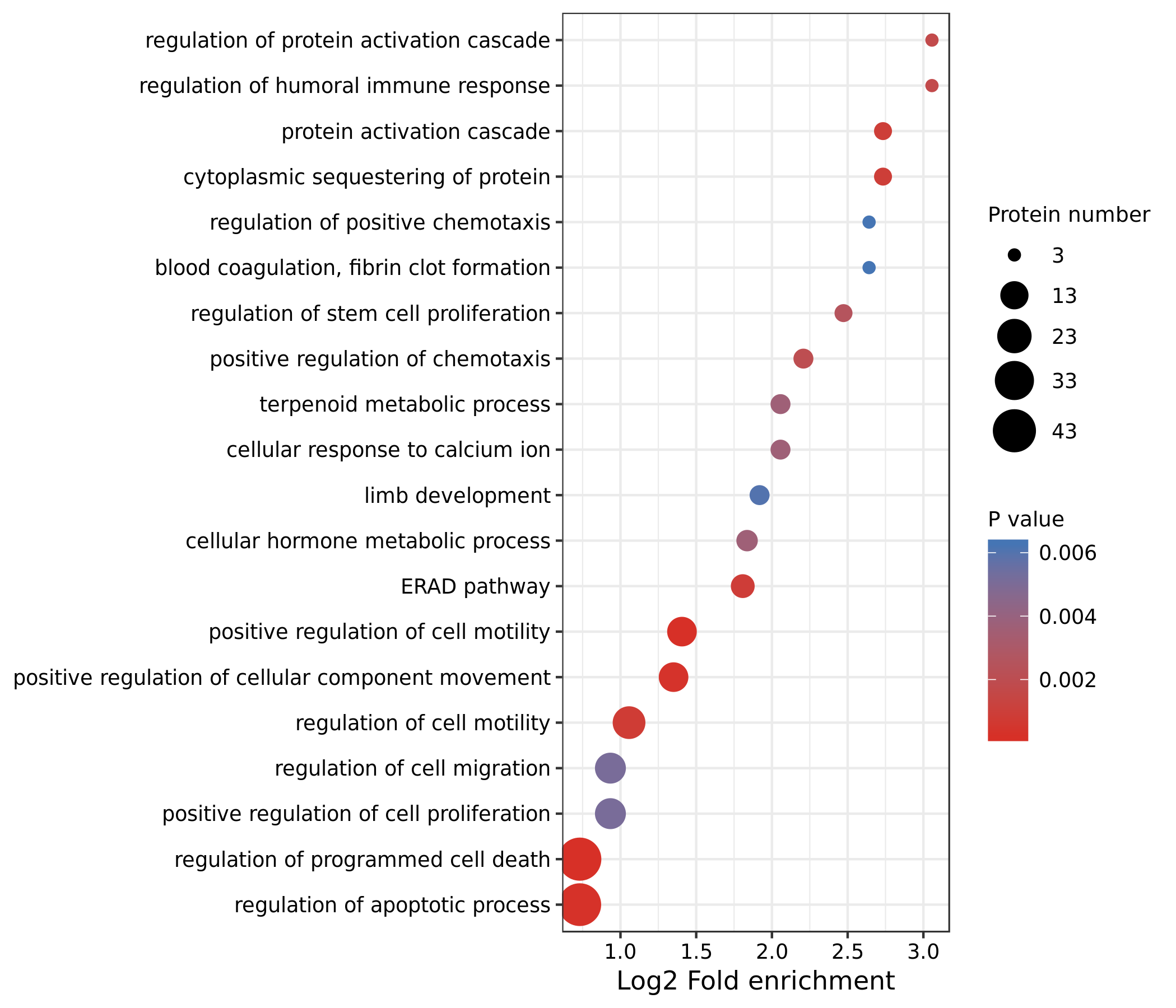

Supplement: Data S1 [file peerj-11-14384-s010.zip › Raw Data/KA076TPAc_FC1.5_update_clean/6-Functional_enrichment/MKN_45SvsMKN_45C/Up-BP.png]

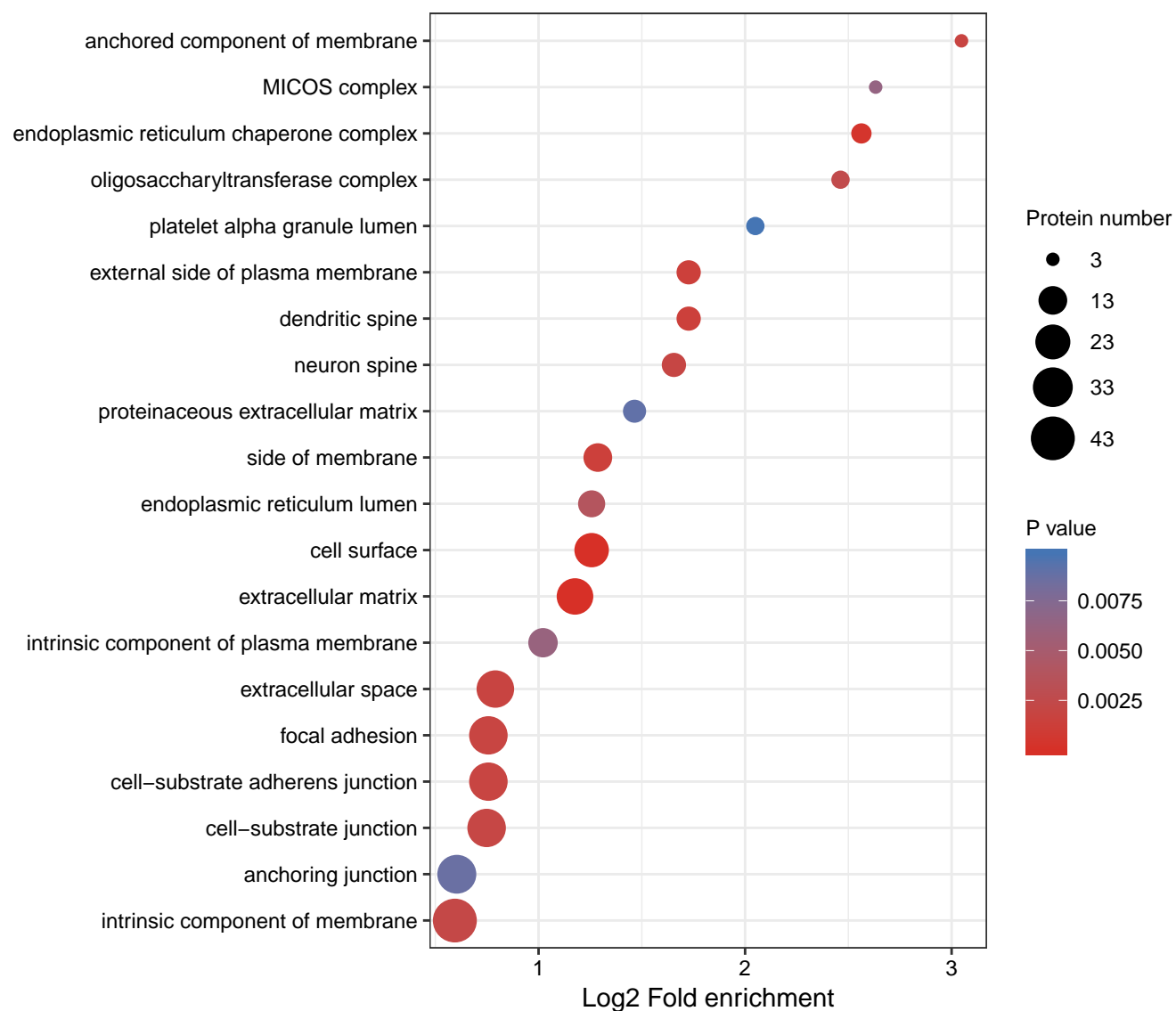

Supplement: Data S1 [file peerj-11-14384-s010.zip › Raw Data/KA076TPAc_FC1.5_update_clean/6-Functional_enrichment/MKN_45SvsMKN_45C/Up-CC.pdf]

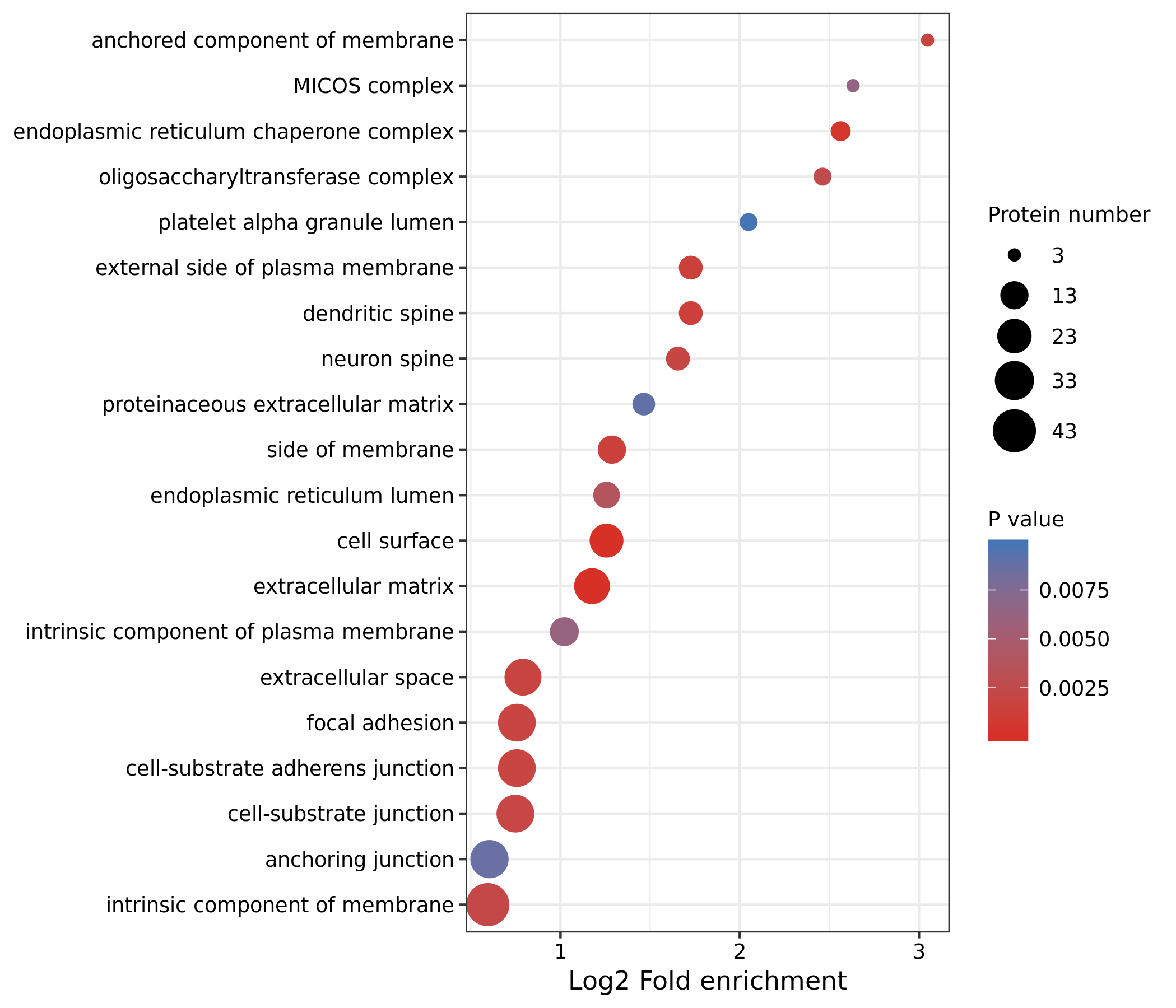

Supplement: Data S1 [file peerj-11-14384-s010.zip › Raw Data/KA076TPAc_FC1.5_update_clean/6-Functional_enrichment/MKN_45SvsMKN_45C/Up-CC.png]

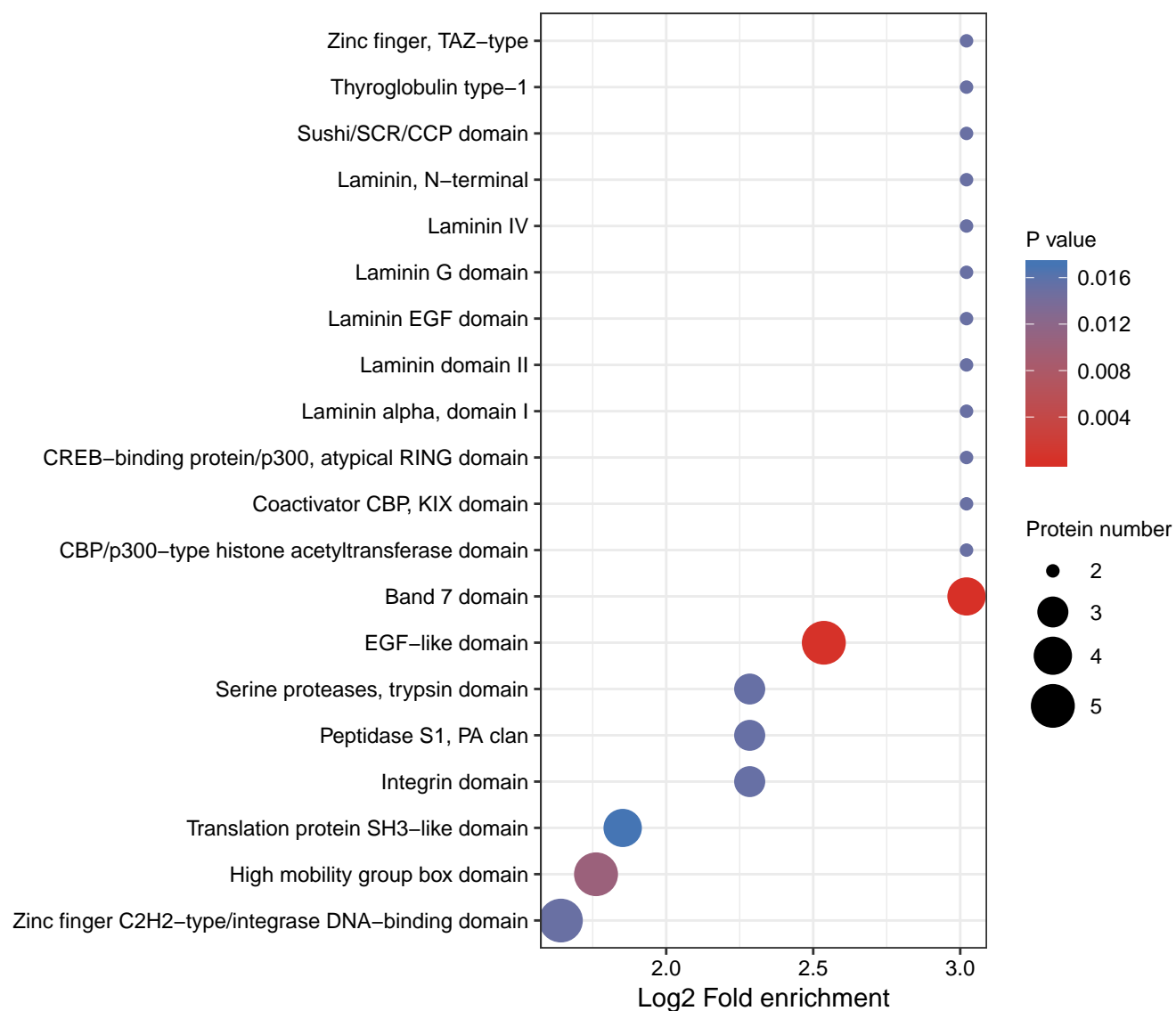

Supplement: Data S1 [file peerj-11-14384-s010.zip › Raw Data/KA076TPAc_FC1.5_update_clean/6-Functional_enrichment/MKN_45SvsMKN_45C/Up-domain.pdf]

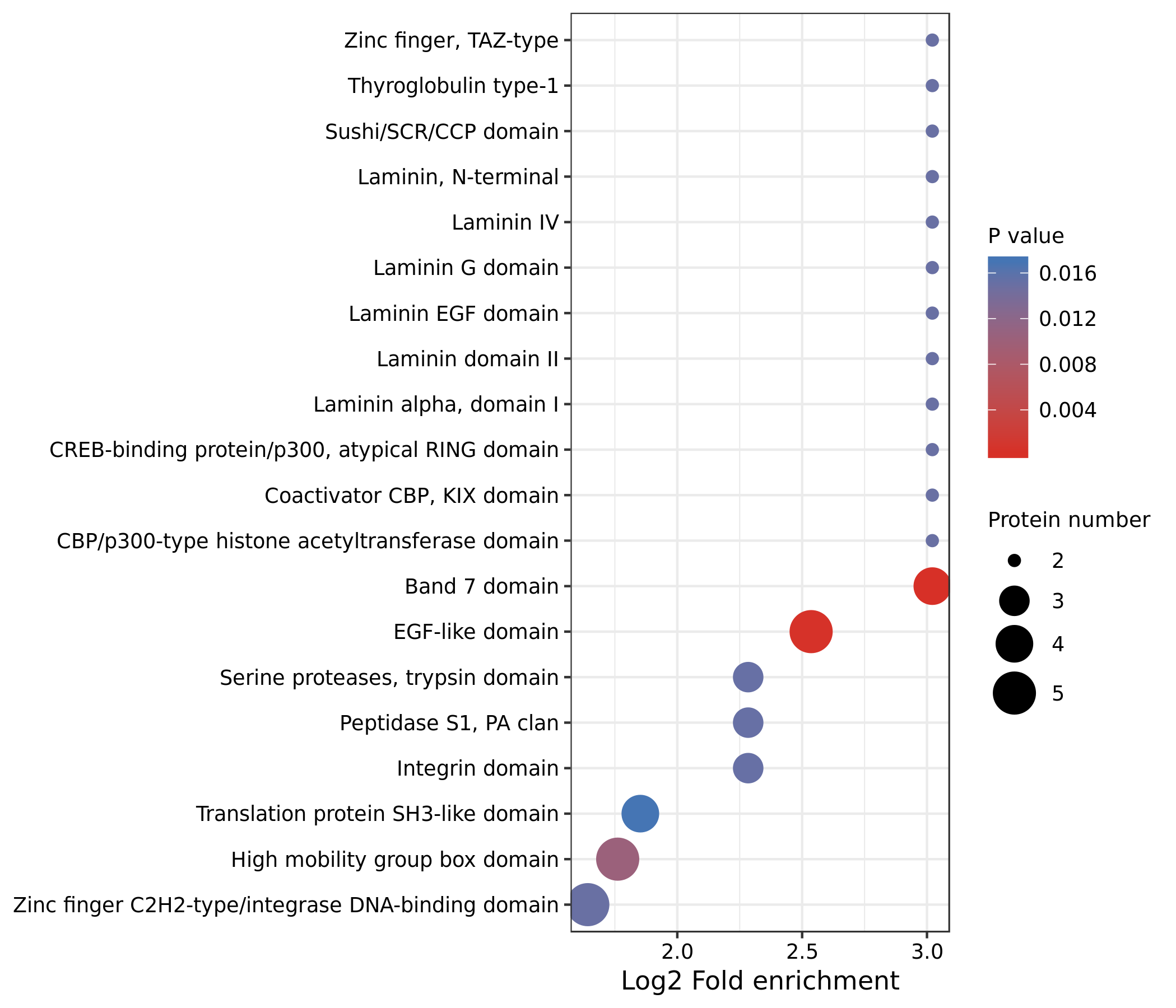

Supplement: Data S1 [file peerj-11-14384-s010.zip › Raw Data/KA076TPAc_FC1.5_update_clean/6-Functional_enrichment/MKN_45SvsMKN_45C/Up-domain.png]

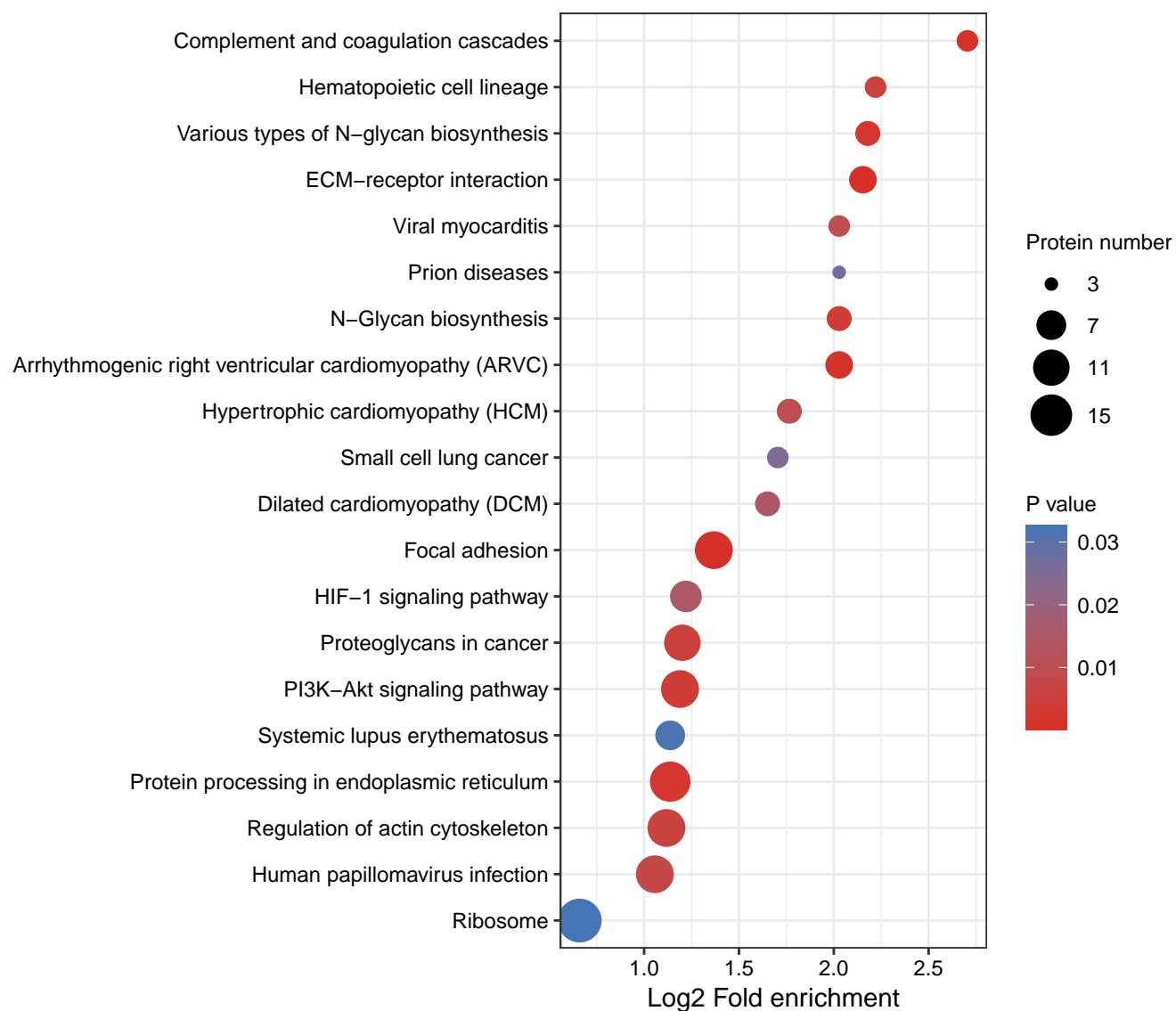

Supplement: Data S1 [file peerj-11-14384-s010.zip › Raw Data/KA076TPAc_FC1.5_update_clean/6-Functional_enrichment/MKN_45SvsMKN_45C/Up-kegg.pdf]

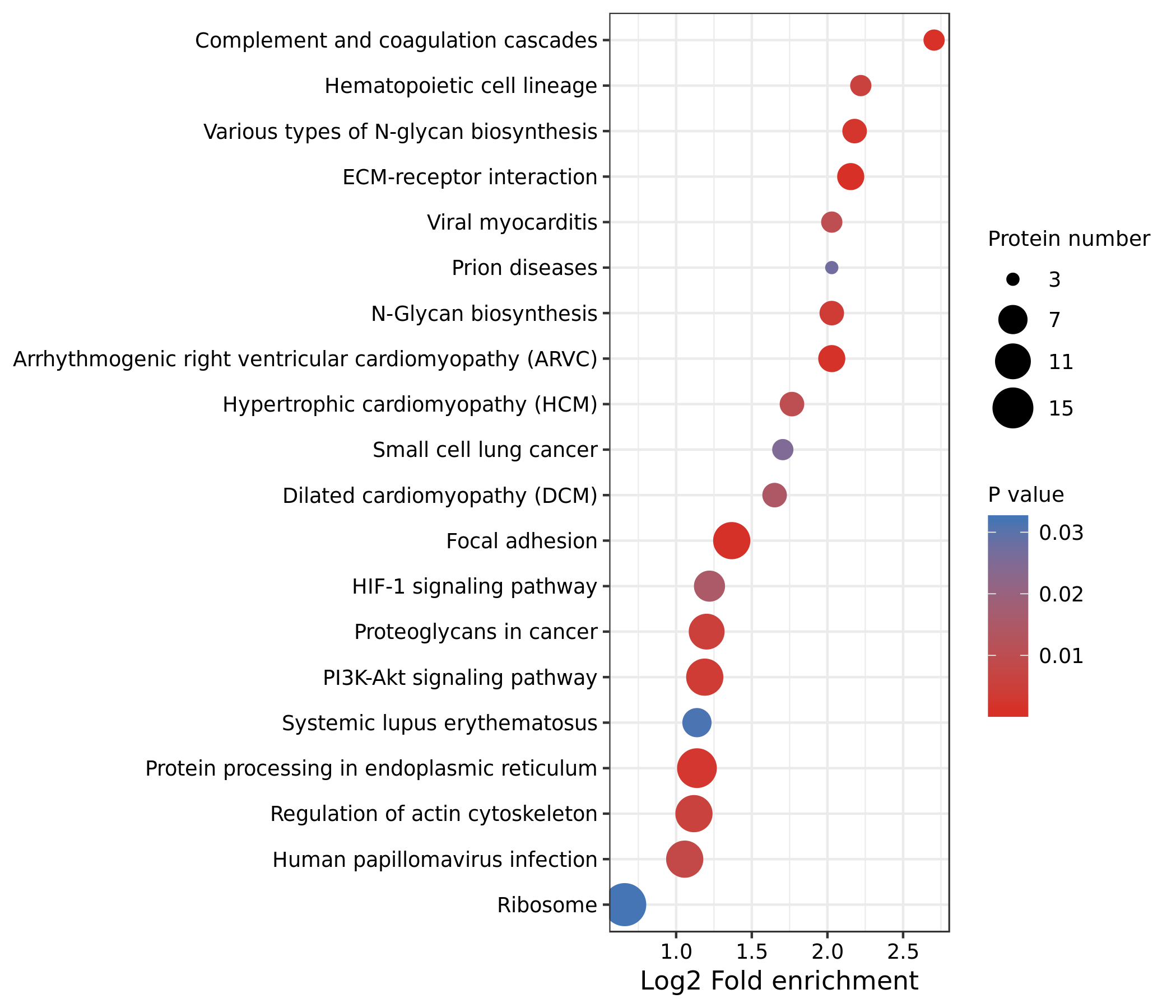

Supplement: Data S1 [file peerj-11-14384-s010.zip › Raw Data/KA076TPAc_FC1.5_update_clean/6-Functional_enrichment/MKN_45SvsMKN_45C/Up-kegg.png]

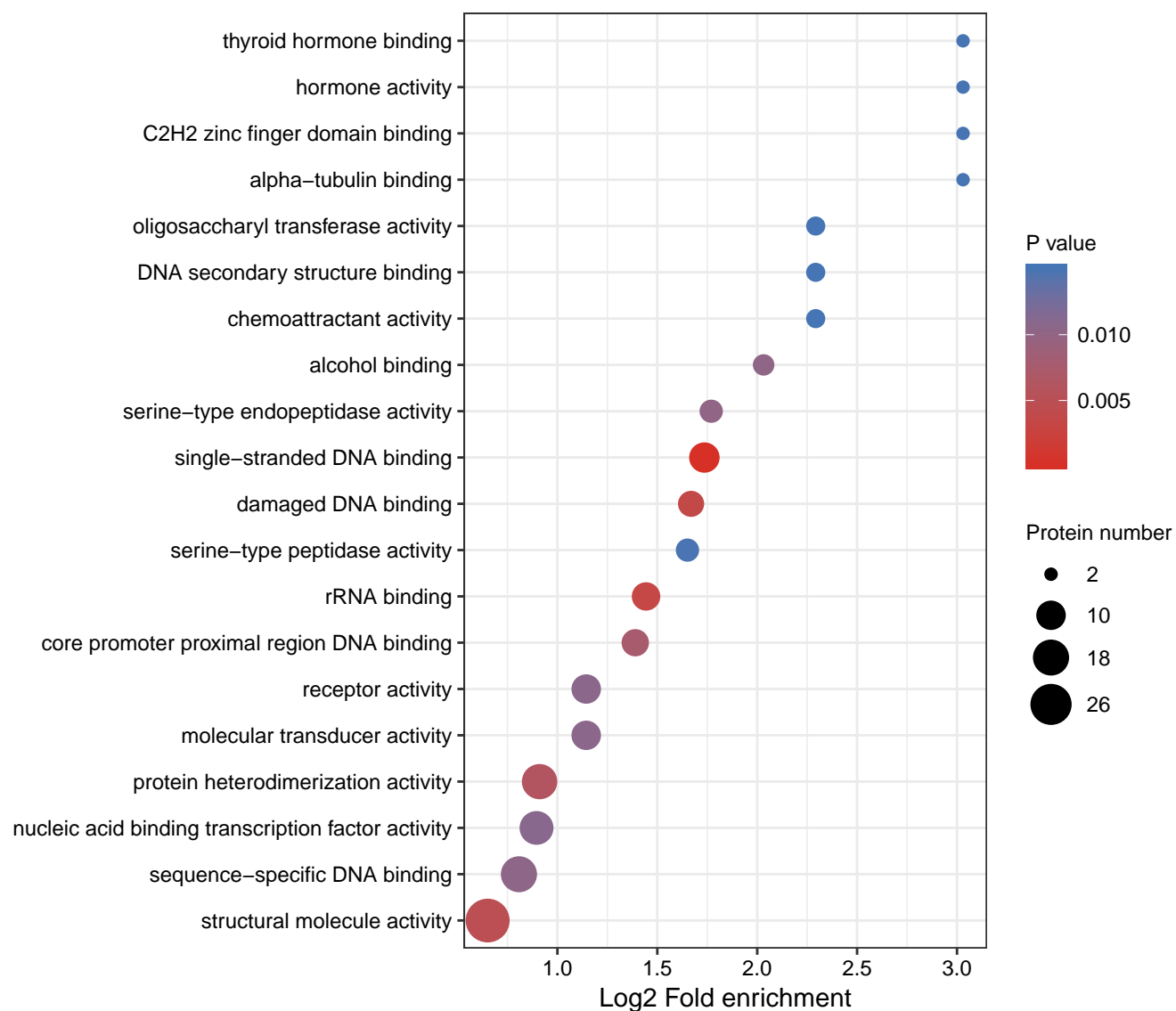

Supplement: Data S1 [file peerj-11-14384-s010.zip › Raw Data/KA076TPAc_FC1.5_update_clean/6-Functional_enrichment/MKN_45SvsMKN_45C/Up-MF.pdf]

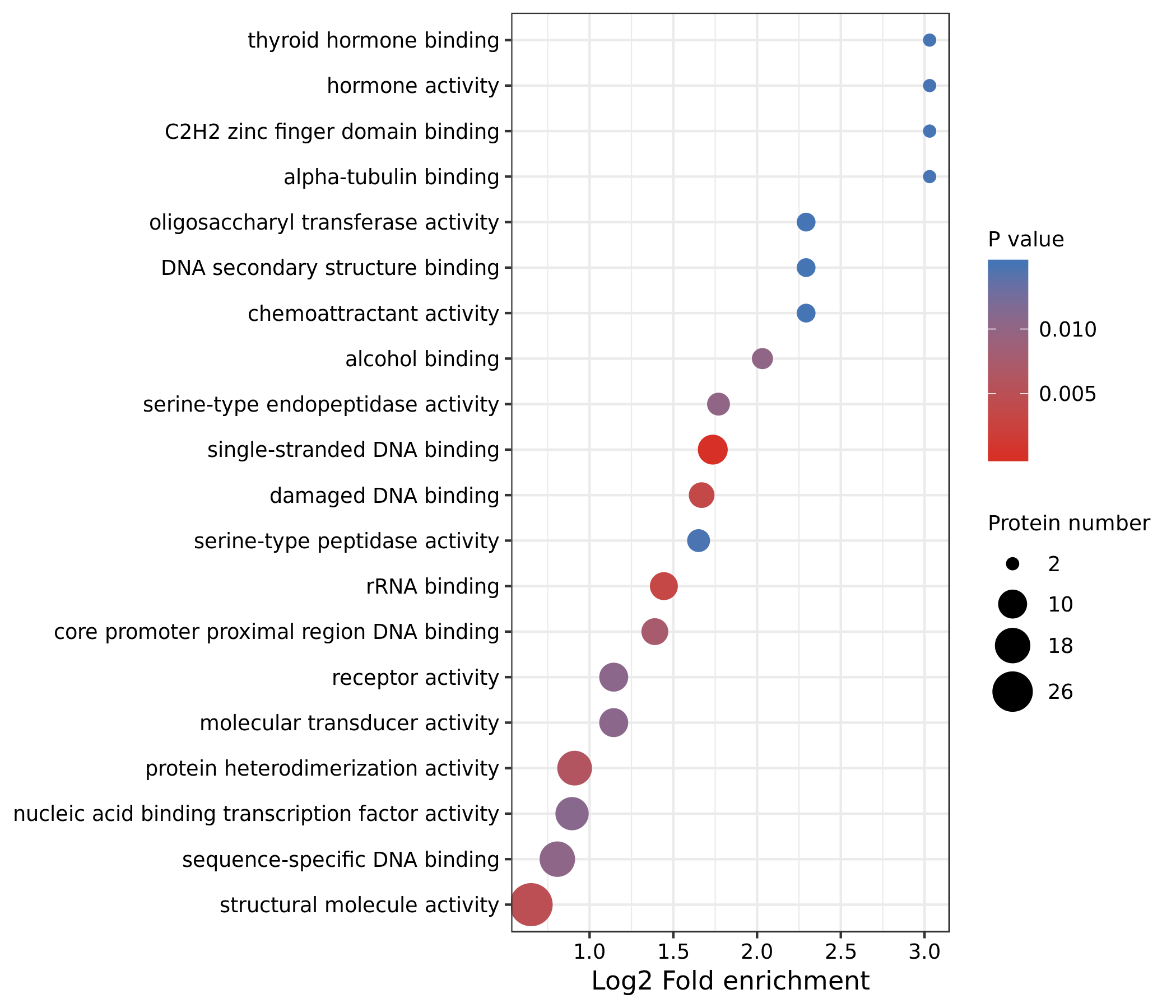

Supplement: Data S1 [file peerj-11-14384-s010.zip › Raw Data/KA076TPAc_FC1.5_update_clean/6-Functional_enrichment/MKN_45SvsMKN_45C/Up-MF.png]

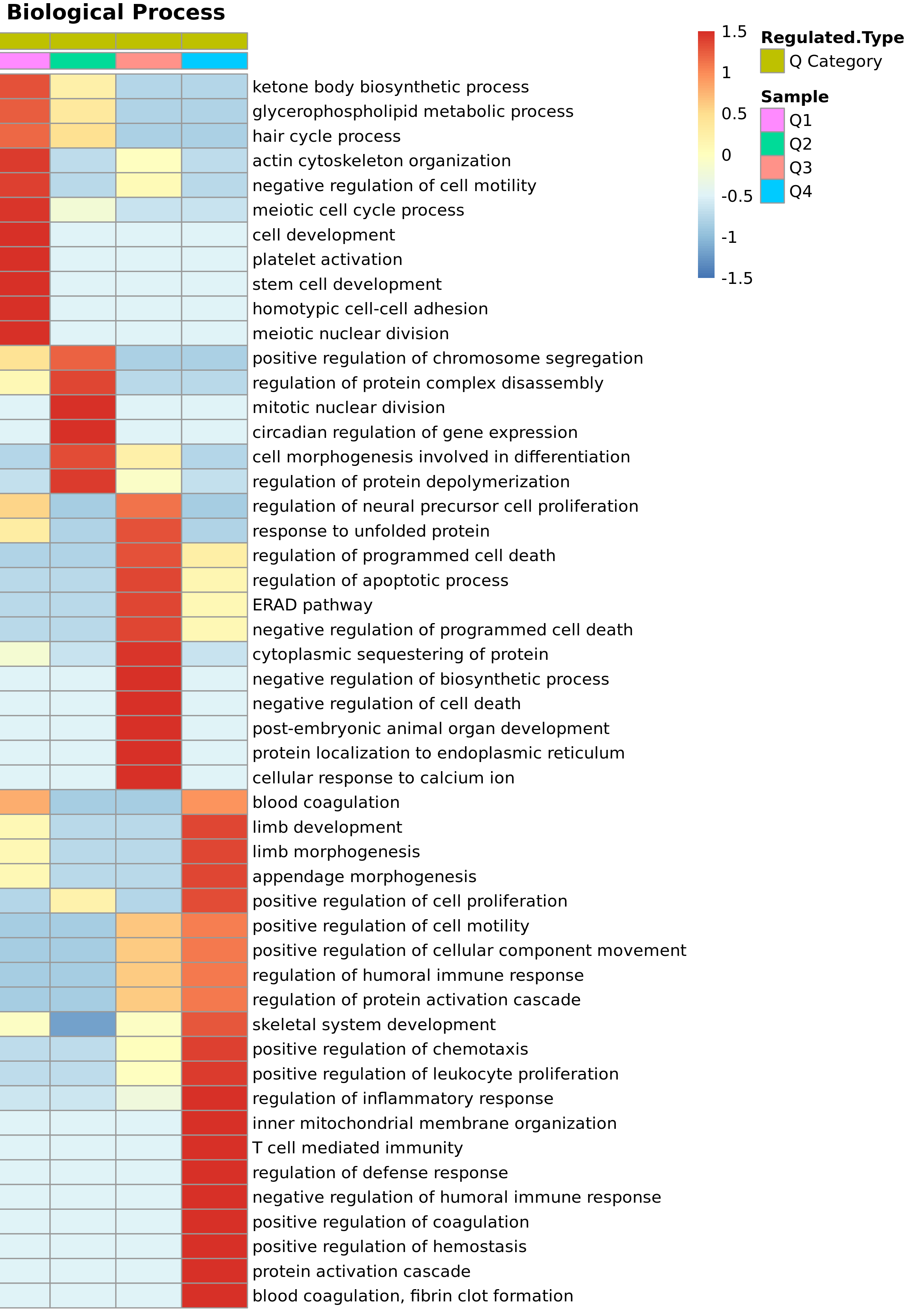

Supplement: Data S1 [file peerj-11-14384-s010.zip › Raw Data/KA076TPAc_FC1.5_update_clean/7-Functional_enrichment_cluster/MKN_45SvsMKN_45C/Biological Process.png]

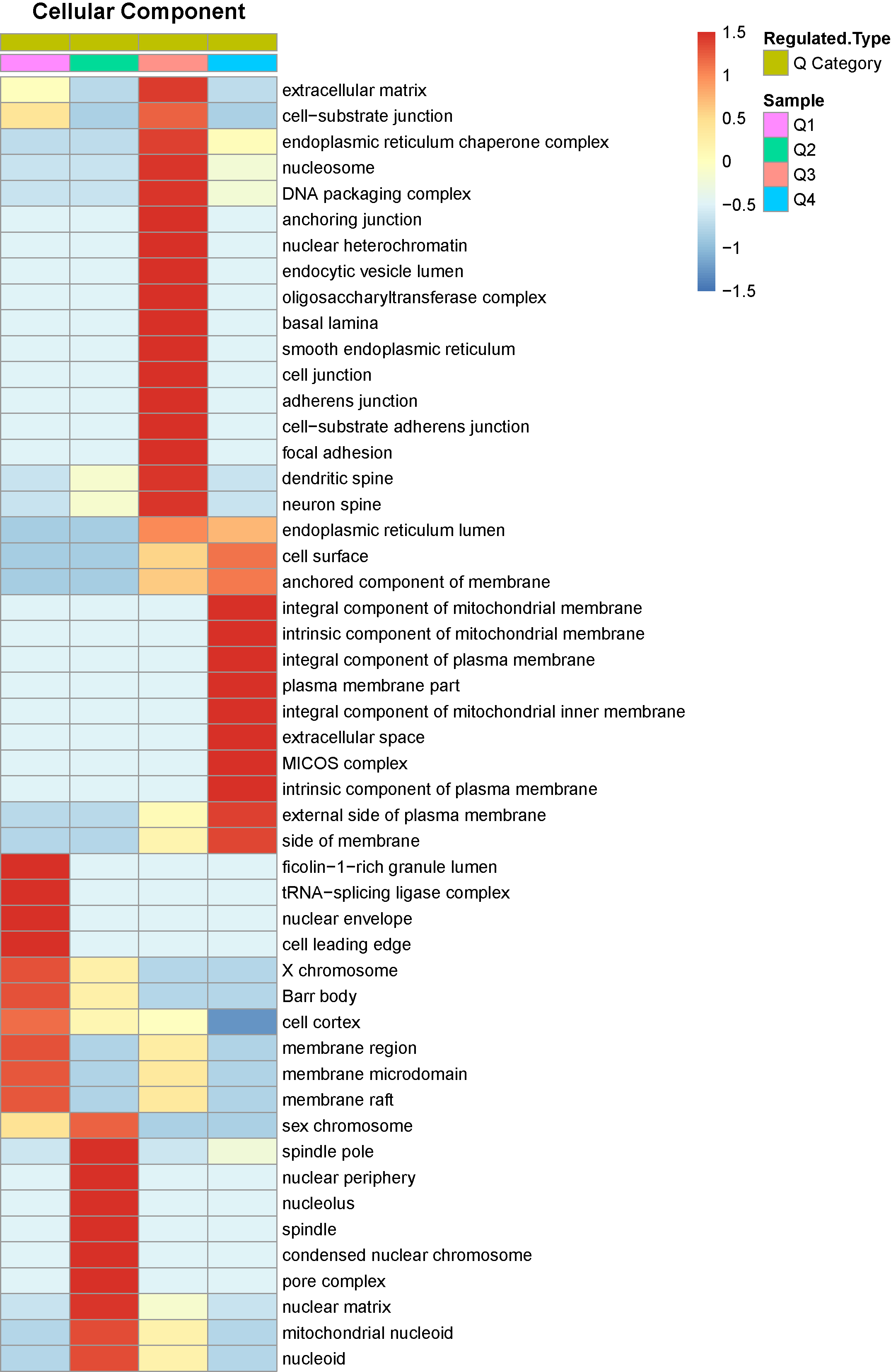

Supplement: Data S1 [file peerj-11-14384-s010.zip › Raw Data/KA076TPAc_FC1.5_update_clean/7-Functional_enrichment_cluster/MKN_45SvsMKN_45C/Cellular Component.png]

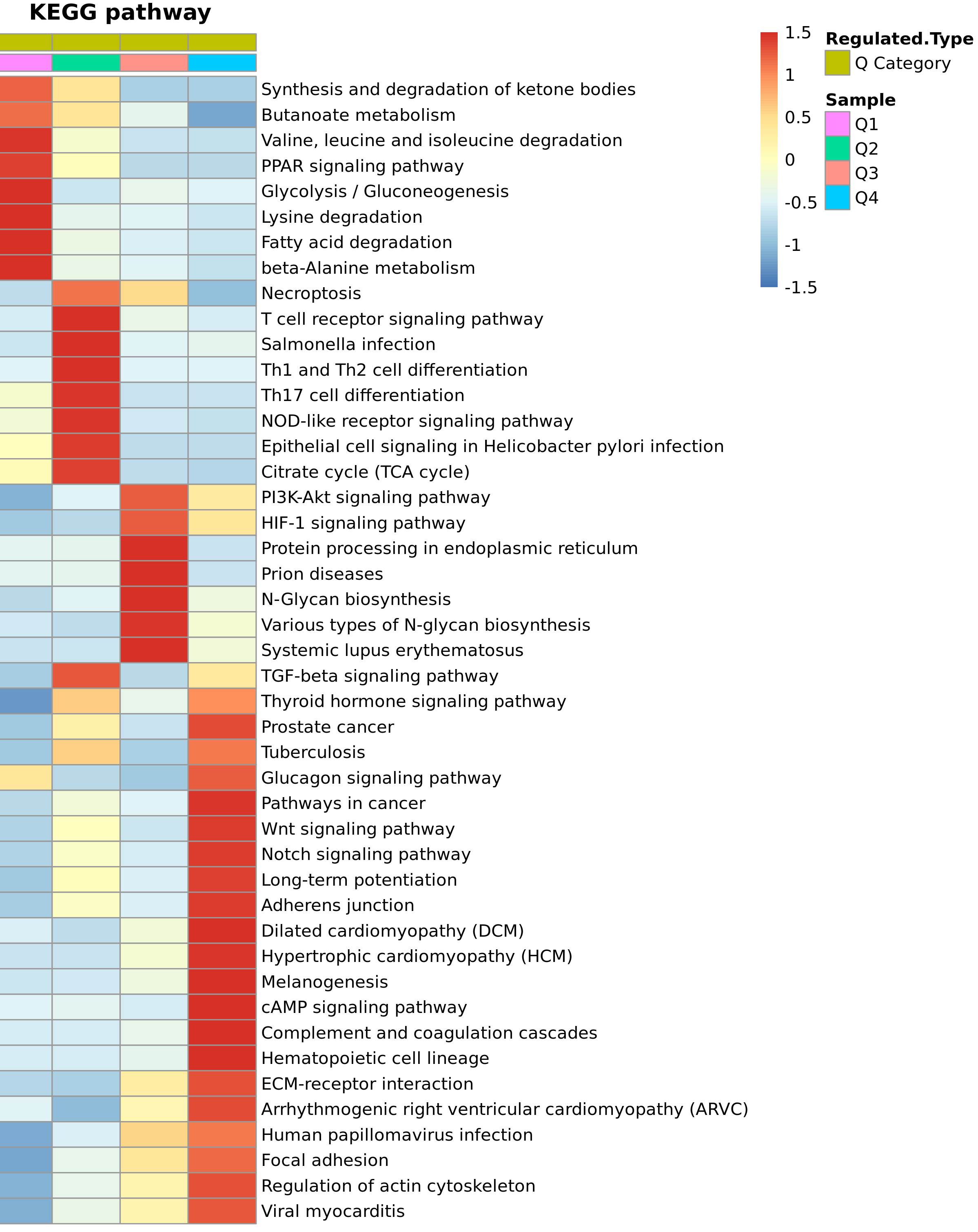

Supplement: Data S1 [file peerj-11-14384-s010.zip › Raw Data/KA076TPAc_FC1.5_update_clean/7-Functional_enrichment_cluster/MKN_45SvsMKN_45C/KEGG pathway.png]

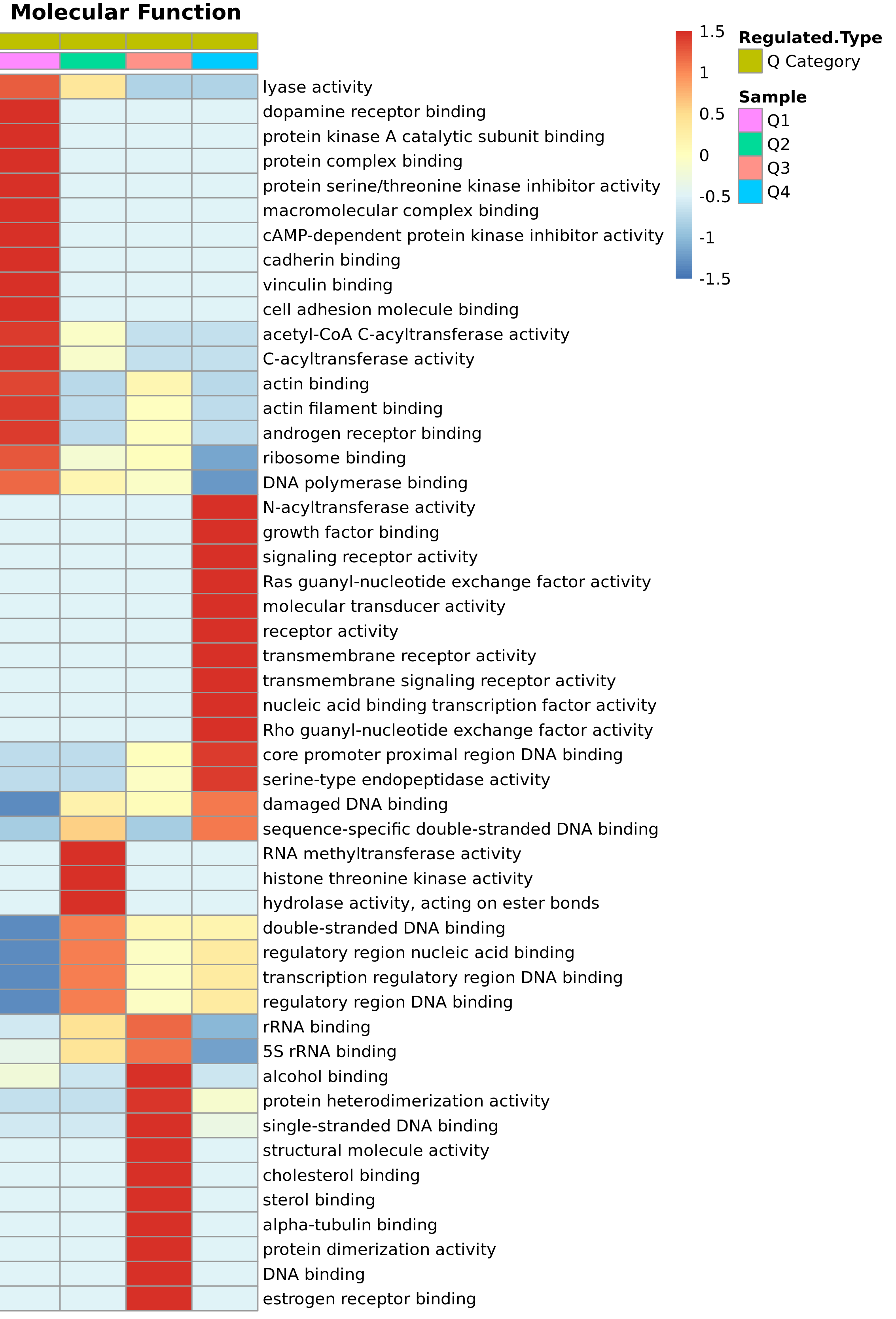

Supplement: Data S1 [file peerj-11-14384-s010.zip › Raw Data/KA076TPAc_FC1.5_update_clean/7-Functional_enrichment_cluster/MKN_45SvsMKN_45C/Molecular Function.png]

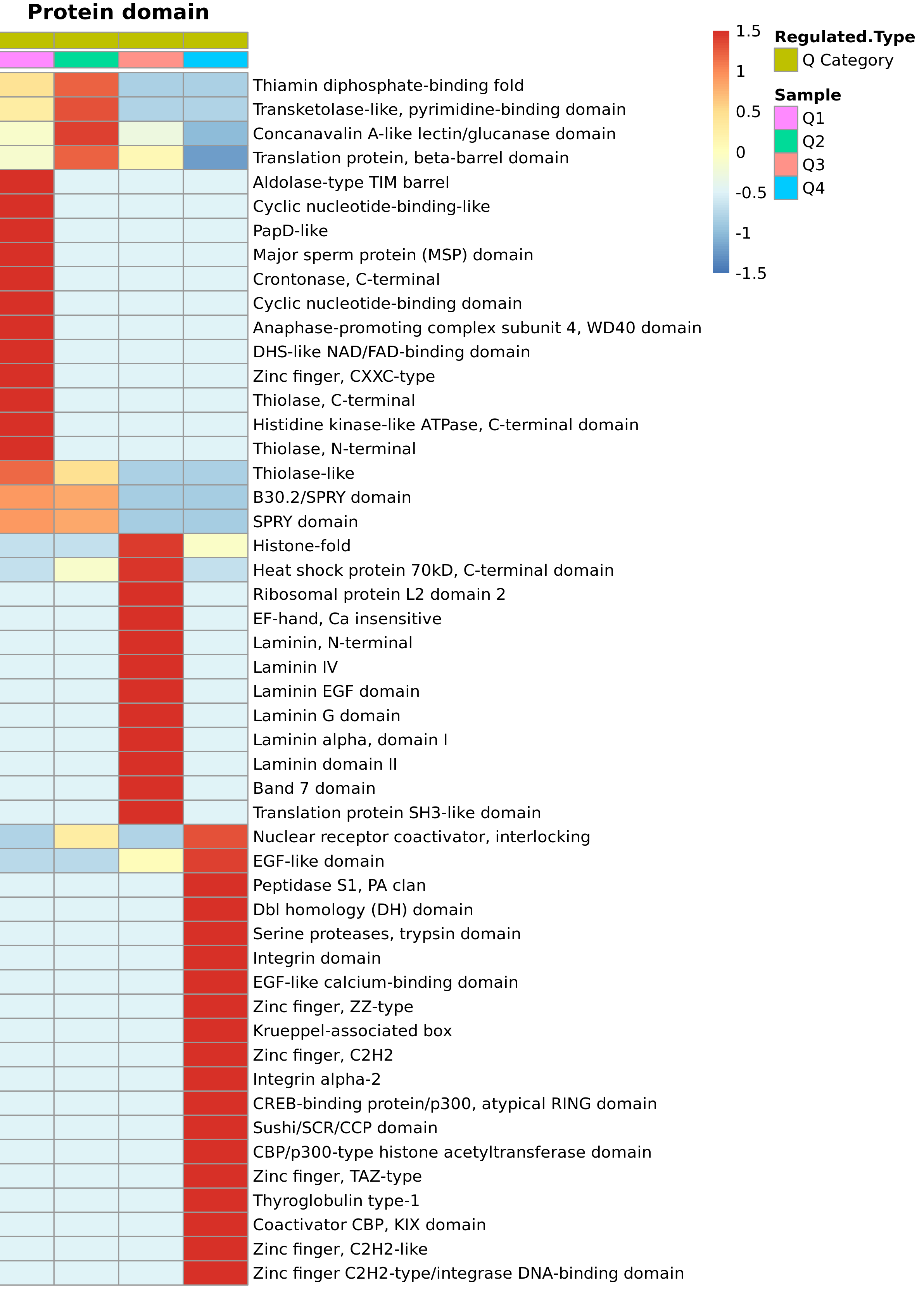

Supplement: Data S1 [file peerj-11-14384-s010.zip › Raw Data/KA076TPAc_FC1.5_update_clean/7-Functional_enrichment_cluster/MKN_45SvsMKN_45C/Protein domain.png]

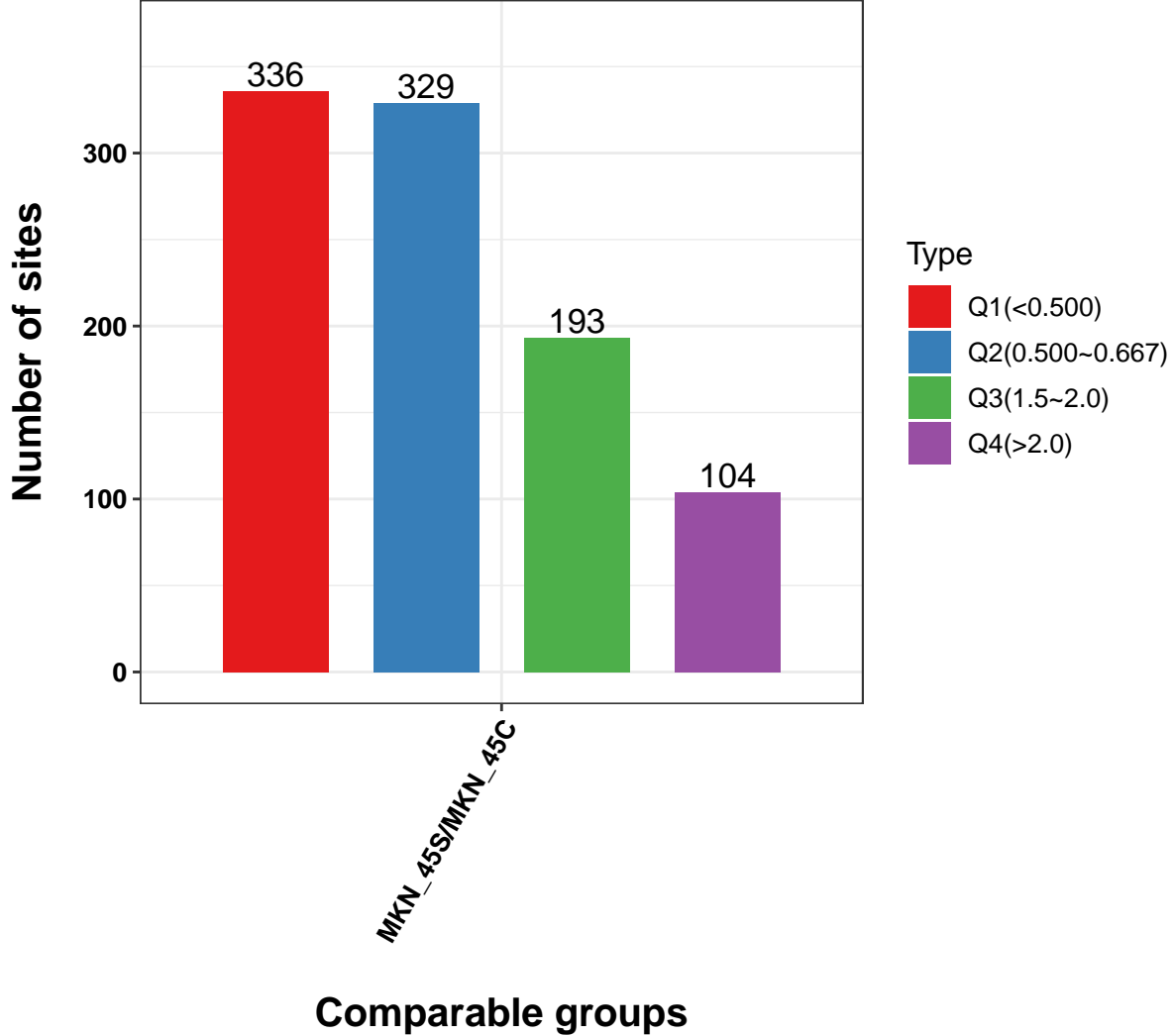

Supplement: Data S1 [file peerj-11-14384-s010.zip › Raw Data/KA076TPAc_FC1.5_update_clean/7-Functional_enrichment_cluster/Q_category/Statistics.pdf]

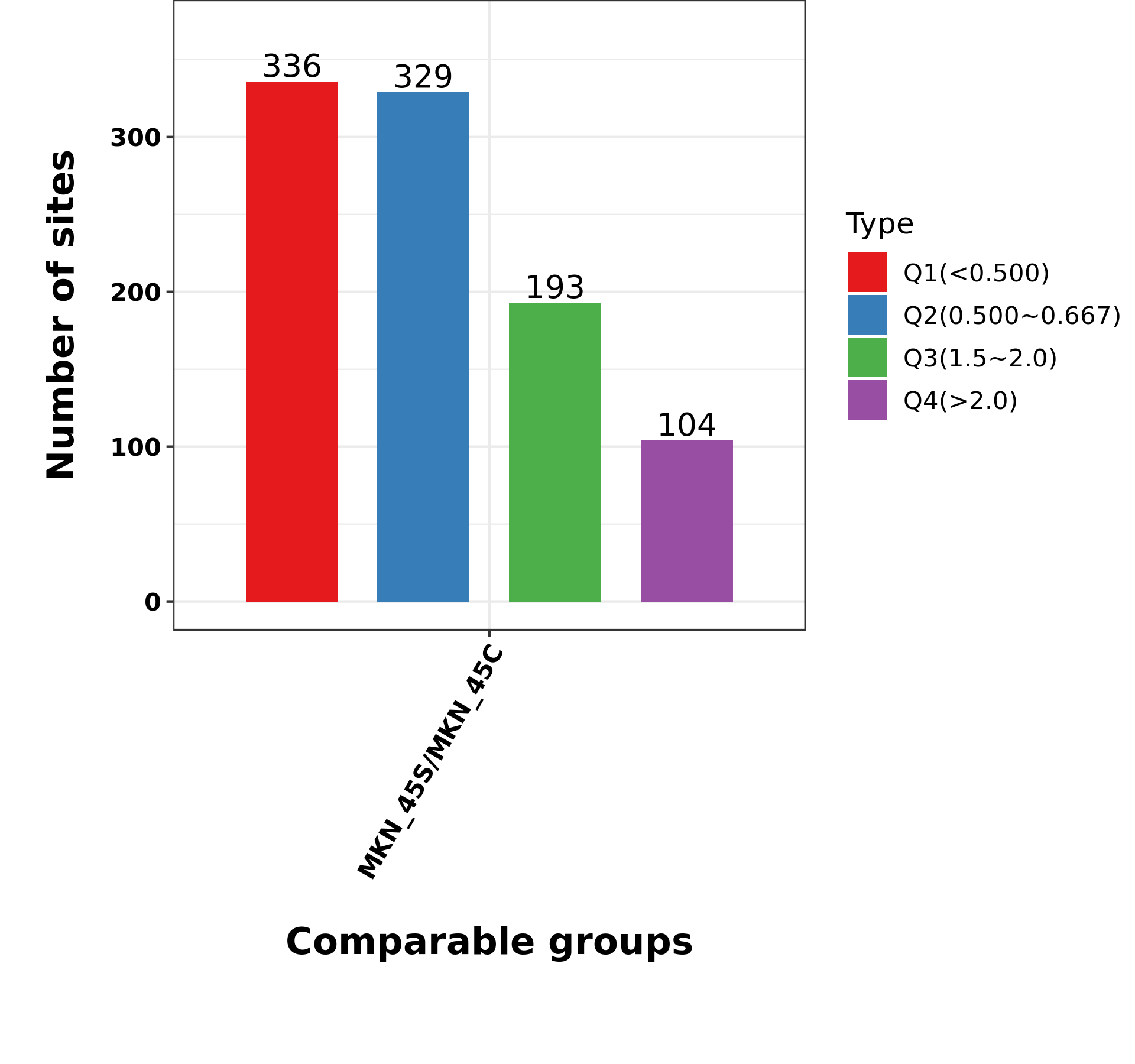

Supplement: Data S1 [file peerj-11-14384-s010.zip › Raw Data/KA076TPAc_FC1.5_update_clean/7-Functional_enrichment_cluster/Q_category/Statistics.png]

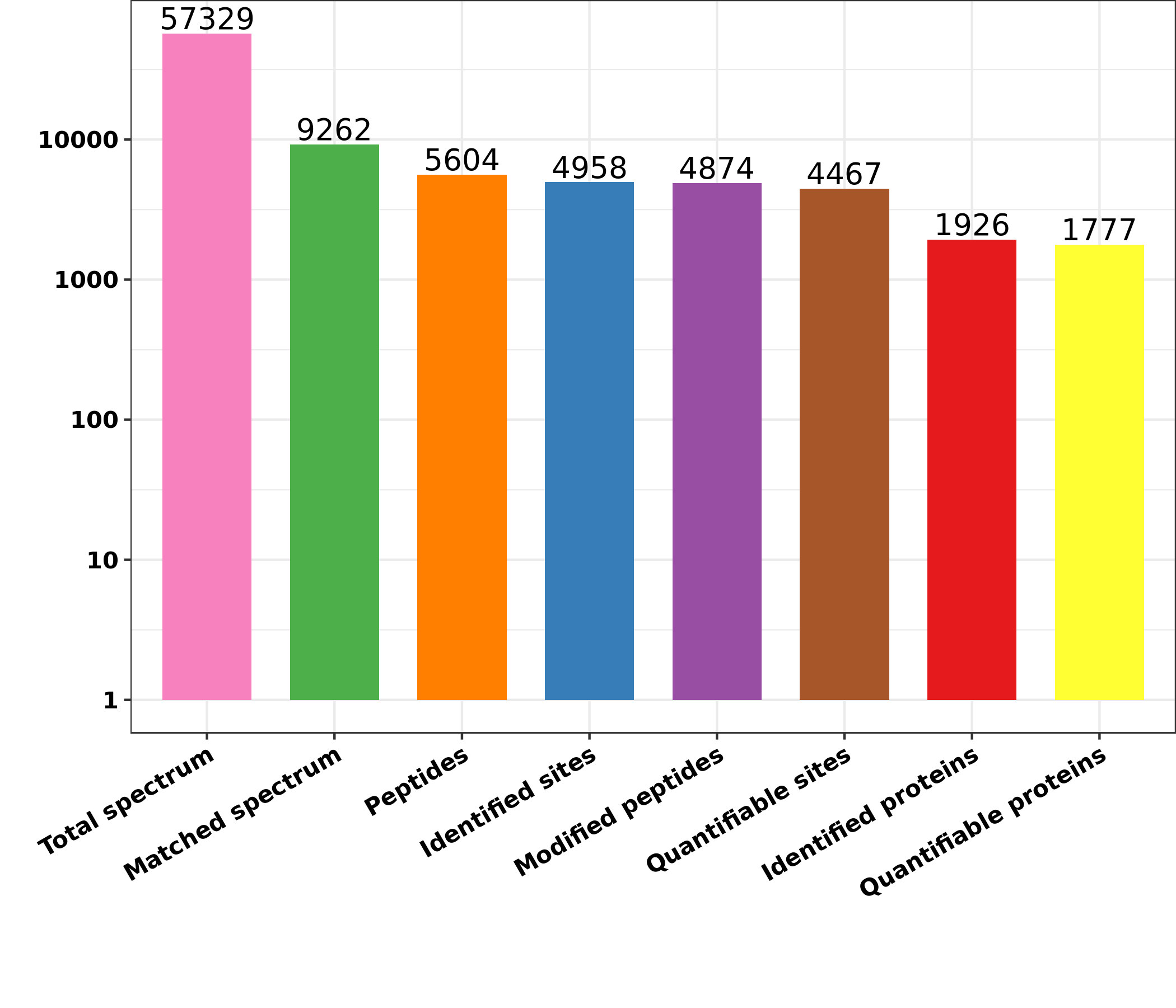

Supplement: Supplemental Information 11 [file peerj-11-14384-s011.png]

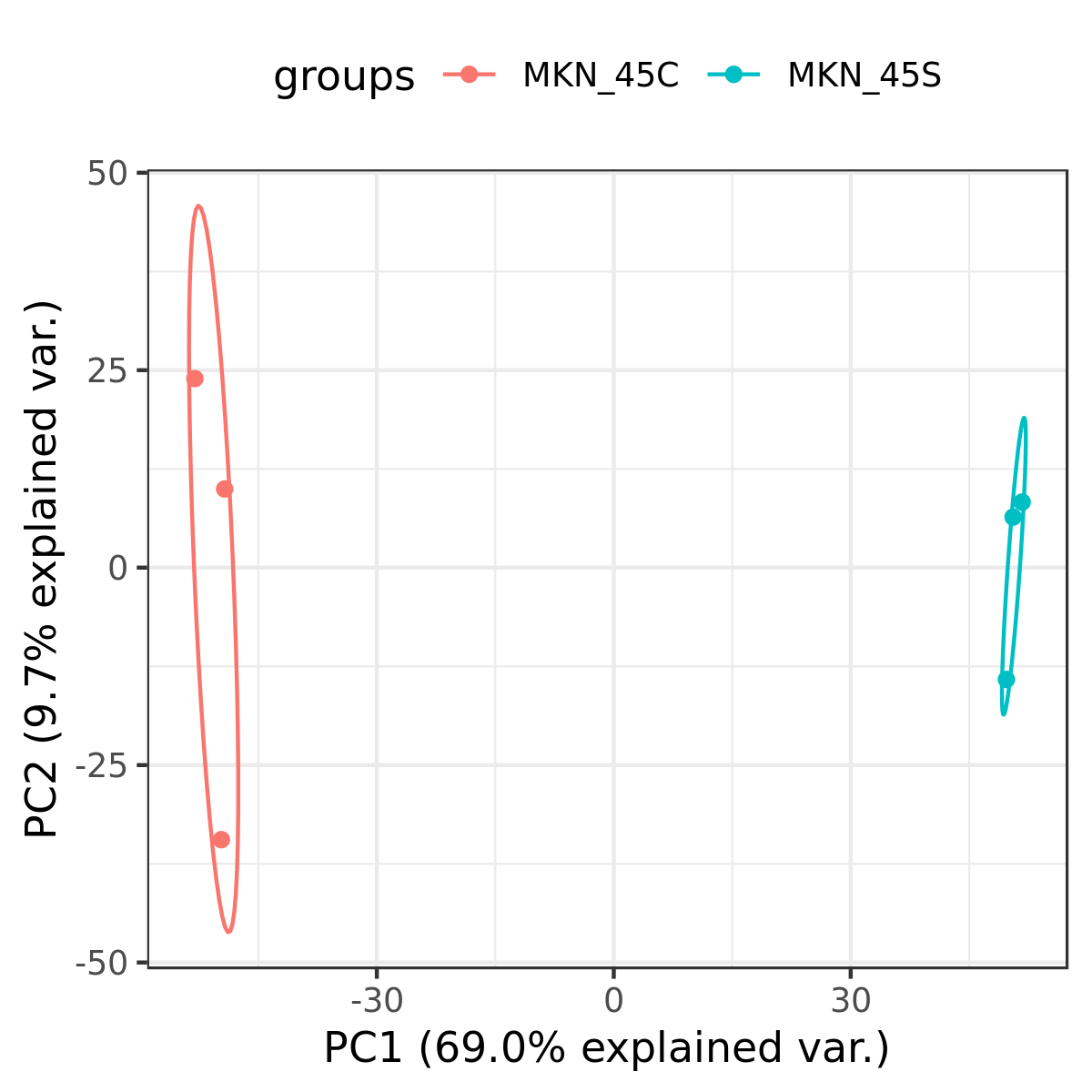

Supplement: Figure S1 [file peerj-11-14384-s012.png]

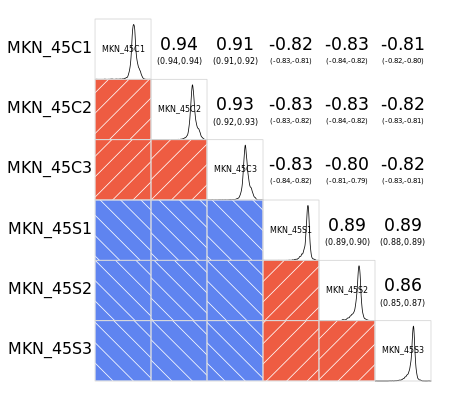

Supplement: Figure S2 [file peerj-11-14384-s013.png]

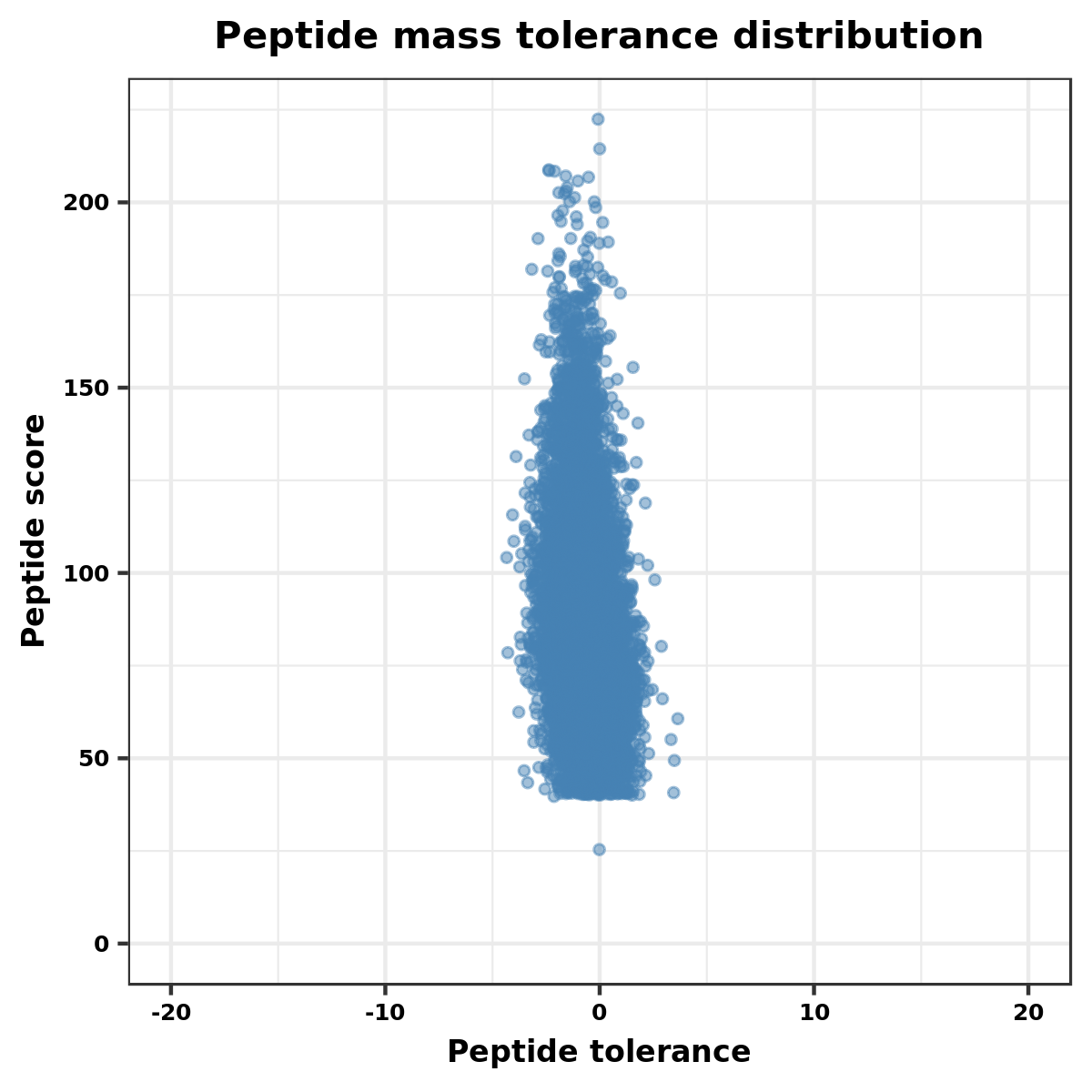

Supplement: Figure S3 [file peerj-11-14384-s014.png]

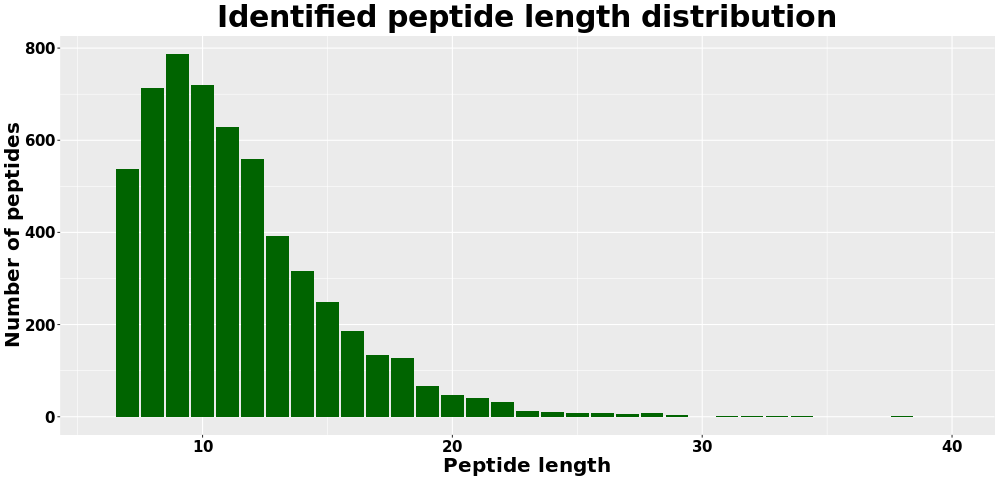

Supplement: Figure S4 [file peerj-11-14384-s015.png]
