# Supplementary material for: Differential expression of lysine acetylation proteins in gastric cancer treated with a new antitumor agent bioactive peptide chelate selenium
Source: PeerJ. 2023 Jan 17;11:e14384. doi: 10.7717/peerj.14384 (PMC9854375; doi:10.7717/peerj.14384)
Supplement: Data S1 [file peerj-11-14384-s010.zip › Raw Data/KA076TPAc_FC1.5_update_clean/1-HTML_report/report_html.html]

TMT乙酰化修饰定量组学分析报告


# TMT乙酰化修饰定量组学分析报告

#### *PTM bioinformatics Team*

#### *项目报告结题日期： 2020-04-07*

### 项目信息：

合同编号: PTM20200119M01  
 项目编号: KA076TPAc  
 客户单位: 内蒙古医科大学  
 联 系 人: 苏秀兰

---

### 联系方式:

李丽娜, 客户经理   
 Email: lina\_li@ptm-biolab.com   
 秦丽欢, 技术支持  
 电话号码：0571-28833567-8003 ；Email: lihuan\_qin@ptm-biolab.com  
 杭州景杰生物科技有限公司   
 浙江省 杭州市 经济技术开发区 6号大街452号2号楼15楼A区

---

# 项目总览

通过TMT标记和乙酰化修饰富集技术以及高分辨率液相色谱-质谱联用的 定量蛋白质组学研究策略，本项目进行了乙酰化修饰蛋白质组学定量研究。我们最终确定到位于1926.0 个蛋白上的4958.0个乙酰化修饰位点，其中1777.0个蛋白的4467.0个位点包含定量信息。差异位点的筛选遵循以下的标准：1.5倍为变化阈值，t-test p-value<0.05 。基于以上数据和标准，我们发现在MKN\_45SvsMKN\_45C比较 组中有297个位点的修饰水平发生上调，665个位点的修饰水平发生下调 。随后，我们对包含定量信息位点的蛋白质进行了系统 的生物信息学分析，包括蛋白注释、功能分类、功能富集及基于功能富集的聚类分析。并综合 以上信息，对下游基于蛋白质组的深入研究提供了参考方向。

# 技术路线

本项目通过将TMT标记、高效液相色谱分级技术、乙酰化修饰肽段的富集技术以及基于质谱的定量蛋白质组学技术等一系列前沿技术的有机结合，对样本中的乙酰化修饰定量组学进行研究。其技术路线如下图所示：

# 1. 生物信息学分析

## 1.1. 修饰鉴定总览

在本项目中，通过质谱分析共得到57329张二级谱图。质谱二级谱图经蛋白理论数据搜库后，得到可利用有效谱图数为9262，谱图利用率为16.2%，通过谱图解析共鉴定到5604条肽段，4874个乙酰化修饰肽段。我们一共鉴定到1926.0个蛋白上的4958.0个乙酰化修饰位点，其中1777.0个蛋白上的4467.0个位点具有定量信息（**表1**）。相关数据及表头详细注解参见：../2-Basic\_analysis/中表格。

##### 表1：质谱数据结果基本统计表

| Total spectrum | Matched spectrum | Peptides | Modified peptides | Identified proteins | Quantifiable proteins | Identified sites | Quantifiable sites |
| --- | --- | --- | --- | --- | --- | --- | --- |
| 57329.0 | 9262 | 5604.0 | 4874.0 | 1926.0 | 1777.0 | 4958.0 | 4467.0 |

#### 图1：质谱数据结果基本统计图

**蛋白差异修饰分析**：本项目通过多次修饰定量重复实验，分别得到了每个样本在多次重复中的定量值。第一步计算比较组中两个样本间修饰位点的差异修饰情况，首先计算出每个样本在多次重复中的定量值的平均值，然后再计算两个样本之间平均值的比值，该比值作为比较组最终的差异修饰相对定量（Ratio）。第二步计算该差异修饰的显著性P-value，首先将各个样本的定量值取log2（以使得数据符合正态分布），然后用双样本双尾T检验方法计算p-value。当p-value<0.05时，以差异修饰量变化超过1.5作为显著上调的变化阈值，小于1/1.5作为显著下调的变化阈值。本项目所有差异修饰的蛋白和位点汇总数据参见**表2**。

##### 表2：修饰水平差异修饰统计信息

| Compared sample name | Type | Up-regulated | Down-regulated |
| --- | --- | --- | --- |
| MKN\_45S/MKN\_45C | Sites | 297.0 | 665.0 |
|  | Proteins | 238.0 | 499.0 |

#### 图2：不同比较组中差异修饰蛋白和修饰位点数量分布柱形图

图3中横轴为修饰位点差异倍数值经过Log2对数转换后的值，纵轴为差异显著性检验p-value值经过-Log10对数转换后的值。图中红色点表示表达量显著上调位点，蓝色点表示表达量显著下调位点。

#### 图3：差异修饰位点火山图

##### MKN\_45SvsMKN\_45C

相关数据参见：../3-Differentially\_expressed\_protein/

## 1.2 样品重复性检验

对于生物重复或技术重复样本，我们检验生物重复或技术重复样本的定量结果是否符合统计学上的一致性。这里我们分别采用了主成分分析（PCA）、相对标准差（RSD）和皮尔森相关性（Pearson’s Correlation Coefficient）三种统计分析方法评估修饰定量重复性。**图4**为所有样本的修饰定量主成分分析结果展示图，图中重复样本之间的聚集程度越好代表定量重复性越好。**图5**为各重复样本间修饰定量值的相对标准差（RSD）绘制的箱线图，当整体RSD值越小，定量重复性越好。**图6**为利用所有样本两两之间计算皮尔森相关系数而绘制的热图。此系数是度量两组数据线性相关程度的值：当皮尔森系数越接近-1为负相关，越接近1为正相关，越接近0为不相关。

#### 图4 重复样本间修饰定量主成分分析二维散点图

#### 图5 重复样本间修饰定量RSD分布箱线图

#### 图6 两两样品间修饰定量的皮尔森相关系数热图

重复性检验数据可见：../2-Basic\_analysis 。

## 1.3 蛋白注释

为了透彻了解数据中鉴定到和定量到的修饰蛋白，我们对这些蛋白的功能、特征等，从基因本论（Gene Ontology，以下简称GO）、蛋白结构域（protein domain）、KEGG通路以及亚细胞结构定位等方面进行了详细的注释。相关数据参见：../4-Protein\_annotation/。

## 1.4 蛋白修饰的基序（motif）分析

蛋白质基序（motif）分析通过对样品中所有乙酰化修饰位点前后氨基酸序列的规律进行统计，计算出发生乙酰化修饰位点区域内氨基酸序列的规律趋势。此类分析可发现修饰的位点序列特征，从而推测或者确定修饰相关的酶。

##### 表3：通过MoMo得到的此修饰的位点特征序列及其富集统计信息

##### 图7 所有鉴定到的乙酰化修饰位点上下游氨基酸的基序富集热图。红色代表此氨基酸在修饰位点附近显著富集，绿色代表此氨基酸在修饰位点附近显著减少。

注: 对于基序分析的数据，请参见 ../9-Motif\_analysis

## 1.5 差异修饰位点对应蛋白的功能分类

### 1.5.1. GO二级注释分类

Gene Ontology（GO）即基因本论, 是一个重要的生物信息学分析方法和工具，用于表述基因和基因产物的各种属性。GO注释分为3个大类：生物进程（Biological Process），细胞组成（Cellular Component）和分子功能（Molecular Function），从不同角度阐释蛋白的生物学作用。我们对差异修饰位点对应蛋白在GO二级注释中的分布进行了统计。

#### 图8 差异乙酰化修饰位点对应蛋白在GO二级分类中统计分布图

##### MKN\_45SvsMKN\_45C

#### 

注：对于所有比较组中GO二级注释的分布情况的具体文件，请参见 ../5-Functional\_classification.

### 1.5.2 亚细胞结构定位分类

我们使用专业软件对于差异修饰蛋白进行了亚细胞结构的预测和分类统计。

#### 图9 差异乙酰化修饰位点对应蛋白的亚细胞结构定位分布图

##### MKN\_45SvsMKN\_45C

#### 

注：所有比较组中亚细胞结构定位的详细文件，请参见 ../5-Functional\_classification.

### 1.5.3 COG/KOG功能分类

COG，即Clusters of Orthologous Groups of proteins。构成每个COG的蛋白都是被假定为来自于一个祖先蛋白，并且因此或者是orthologs或者是paralogs。Orthologs是指来自于不同物种的由垂直家系（物种形成）进化而来的蛋白，并且典型的保留与原始蛋白有相同的功能。COG是NCBI的数据库。COG的中文释义即“同源蛋白簇”。COG分为两类，一类是原核生物的，另一类是真核生物。原核生物的一般称为COG数据库；真核生物的一般称为KOG数据库。我们数据库比对分析，将差异修饰蛋白进行了COG/KOG功能分类统计。

#### 图10 差异乙酰化修饰位点对应蛋白的COG/KOG功能分类分布图

##### MKN\_45SvsMKN\_45C

#### 

注：所有比较组中COG/KOG功能分类的详细文件，请参见 ../5-Functional\_classification.

## 1.6 差异修饰位点对应蛋白的功能富集分析

根据1.1中对于鉴定到的所有含有修饰位点蛋白的注释以及差异修饰位点对应蛋白的筛选，我们进行了GO分类、KEGG通路和蛋白结构域（domain）三个层面的富集分析，目的是检测差异修饰是否在某些功能类型有显著性的富集趋势。对于富集检验（此处运用Fisher’s exact test即费希尔精确检验）得到的p-value通过气泡图方式展现了差异修饰蛋白显著富集（p<0.05）的功能分类和通路。气泡图中给出了最显著富集的前20个分类的结果。气泡图中纵轴为功能分类或通路，横轴数值为差异修饰蛋白在该功能类型中所占比例相比于鉴定蛋白所占比例的变化倍数的Log2转换后的数值。圆圈颜色表示富集显著性p-value，圆圈大小表示功能类或通路中差异修饰蛋白个数。

### 1.6.1 GO富集

我们将GO分类中的三大类（Biological Process，Cellular Component，Molecular Function）分别进行了差异修饰蛋白富集分析。相关信息见“../6-Functional\_enrichment”文件夹。

#### 图11 差异乙酰化修饰位点对应蛋白在GO功能分类中富集分布气泡图。

##### MKN\_45SvsMKN\_45C Biological Process

##### MKN\_45SvsMKN\_45C Cellular component

##### MKN\_45SvsMKN\_45C Molecular Function

#### 图12 差异乙酰化修饰位点对应蛋白GO富集结果的有向无环图展示。图中圆圈表示差异修饰蛋白所在的GO分类，红色代表差异修饰蛋白极其显著(P<0.01)富集GO分类，黄色代表差异修饰蛋白显著(P<0.05)富集GO分类，蓝色表示非显著富集GO分类。带箭头的连线表示GO分类的上下层级关系，圆圈大小代表富集程度(Fold Enrichment)。

##### MKN\_45SvsMKN\_45C

#### 

注：对于比较组中GO富集检验条形图，请参见 “../6-Functional\_enrichment”.

### 1.6.2 KEGG 通路富集

KEGG是连接已知分子间相互作用的信息网络，如代谢通路、复合物、生化反应。KEGG途径主要包括：代谢、遗传信息处理、环境信息处理、细胞过程、人类疾病、药物开发等。富集分析得到的KEGG通路可以网页形式进行可视化展示。

#### 图13 差异乙酰化修饰位点对应蛋白显著富集的某KEGG通路示意图。图中红色表示差异上调蛋白；绿色表示差异下调蛋白；黄色表示此节点中存在多个蛋白，且包含差异上调及差异下调蛋白。

注：对于所有比较组中富集到的KEGG通路图，请参见“../8-Enrichment\_pathway\_image”。

#### 图14 差异乙酰化修饰位点对应蛋白在KEGG通路中富集分布气泡图

##### MKN\_45SvsMKN\_45C

#### 

注：对于所有比较组中KEGG富集情况，请参见 “../6-Functional\_enrichment”。

### 1.6.3 蛋白结构域富集

蛋白质结构域是指在不同蛋白质分子中重复出现的某些组分，具有相似的序列、结构和功能，是蛋白质进化的单元。结构域的长度通常在25个氨基酸和500个氨基酸长度之间。

#### 图15 差异乙酰化修饰位点对应蛋白在蛋白结构域分类中富集分布气泡图。

##### MKN\_45SvsMKN\_45C

#### 

注：对于所有比较组中结构域富集详细结果，请参见 “../6-Functional\_enrichment”。

## 1.7 聚类分析

对于修饰水平差异变化的位点，我们根据其差异修饰倍数将其分成了4个部分，称为Q1到Q4，如图所示。然后，我们对于每一个Q组分别进行GO、KEGG及蛋白结构域的富集，并进行聚类分析，旨在找到不同差异修饰倍数的位点功能的相关性。

#### 图16 差异修饰位点根据倍数分为Q1-Q4的个数分布

聚类方法：根据富集分析得到的富集检验（Fisher’s exact test）p值使用分层聚类的方法将不同组中的相关功能聚到一起，绘制为热图（heatmap）。热图的横向代表不同比较组，纵向为差异修饰富集相关功能（GO，KEGG pathway，protein domain）的描述。不同组的差异修饰蛋白与功能描述对应的色块表示富集程度强弱。红色代表富集程度强，蓝色代表富集程度弱。

#### 图17 基于GO分类、KEGG通路和蛋白结构域富集的聚类分析热图。

##### Biological Process

##### Cellular Component

##### Molecular Function

##### KEGG Pathway

##### Protein Domain

#### 

注：此结果请参见 “../7-Functional\_enrichment\_cluster”。

## 1.8 蛋白互作网络

将不同比较组中筛选得到的差异修饰蛋白数据库编号或蛋白序列，通过与STRING（v.11.0）蛋白网络互作数据库比对后，按照confidence score >0.7(high confidence)提取得到差异蛋白互作关系。然后通过R package “networkD3”工具对差异蛋白互作网络进行可视化展示。如下图所示：图中圆圈表示差异修饰蛋白，不同颜色代表蛋白的差异修饰情况（蓝色为下调蛋白，红色为上调蛋白）。圆圈大小代表差异修饰蛋白与其互作蛋白个数。圆圈越大表示与其互作的蛋白越多，说明该蛋白在网络中越重要。为了能清晰的展示蛋白与蛋白之间互作关系，我们筛选出了前50互作关系最紧密的蛋白绘制了蛋白互作网络。

#### 图18 差异修饰蛋白互作网络图

##### MKN\_45SvsMKN\_45C

#### 

注：此结果请参见 “../10-PPI”。

# 2. 对于开展进一步研究的建议（供参考）

如项目报告所陈述，您的项目在蛋白质组学分析的层面上已相对完整周详。基于项目的现有数据，如果您计划开展进一步的研究工作，我们提出如下建议供您参考：

## 2.1. 功能蛋白筛选的一般性原则

1）选取蛋白表达或修饰水平显著差异的蛋白,建议至少选取5个蛋白或修饰位点加以验证1,2；  
 2）可优先选取功能上已有相关报道或者与本研究实验体系有潜在关系的蛋白；   
 3）选取生物信息学分析中得到的某些特定功能、通路、组分中差异表达显著的关键蛋白；  
 4）选取功能上与某一特定生物学过程密切相关的蛋白，如信号通路中的受体蛋白、重要转录因子或者酶等；  
 5）选取该蛋白是某重要蛋白复合物的核心蛋白；  
 6）关于目的蛋白修饰位点的选择，可优先考虑该修饰位点位于目的蛋白的重要功能域、与其它蛋白相互作用的domain、酶蛋白的活性催化中心等关键功能调控区域3。

## 2.2. 蛋白质表达或蛋白质修饰定量改变验证的基本方法

1）筛选出目的蛋白后，可采用基于抗体的Western Blot、免疫组化、ELISA等方法验证不同样品（如实验组和对照组）中蛋白表达差异。当没有现成抗体可使用时，可以考虑使用RT-PCR方法来验证，但是由于存在转录后调控、蛋白质翻译水平调节和蛋白质降解等现象，转录水平的变化和蛋白水平的变化未必能完全对应。  
 2）筛选出修饰位点之后，可利用基于蛋白质修饰的位点特异性抗体的Western Blot、免疫组化、ELISA等方法验证不同样品（如实验组和对照组）中蛋白质修饰的差异。对于没有现成位点特异性抗体可以使用的，可以考虑将目的蛋白IP下来后使用蛋白质修饰的泛抗体WB来检测4。这种方法实际检测的是该蛋白的整体修饰水平，但由于一个蛋白可能存在多个同种修饰位点，因此这种验证方式未必准确可靠。此外，也可以考虑将目的蛋白IP下来后采用质谱定量分析的方法进一步确定修饰水平的改变程度。

## 2.3. 目的蛋白功能研究的一般性建议

1）分子生物学层面：对于表达水平差异的蛋白质的研究，可使用反向遗传学reverse genetics的方法（knockdown，knockout，CRISPR），观察基因改变后导致的表型变化，并结合目的基因回补实验，进一步证实目的蛋白与表型之间的关联。对于修饰水平差异的蛋白和位点，可以对该位点进行体外定点突变并在研究系统中引入点突变site mutant的方式加以研究，例如磷酸化位点S/T突变为A模拟非修饰状态，突变为D/E模拟组成型修饰状态；乙酰化位点K突变为R模拟非修饰状态，突变为Q模拟组成型修饰状态；琥珀酰化因为具有负电荷(酸性)，其修饰位点K突变为R模拟非修饰状态，而突变为E模拟组成型修饰状态3,5,6。  
 2）生物化学层面：生物化学层面首先要考虑的是目的蛋白的相互作用蛋白的鉴定，常见的方法是利用目的蛋白的内源性抗体，通过免疫沉淀（Immunoprecipitation，IP）结合质谱分析的方法鉴定相互作用蛋白；在没有内源性抗体的情况下，也可考虑采用引入带有标签的过表达目的蛋白的pull down实验并结合质谱分析的方法鉴定相互作用蛋白。如果目标蛋白是酶，可通过检测修饰前后目的蛋白的酶动力学参数变化，分析该修饰对酶活性的影响。某些酶蛋白修饰后，可能导致其稳定性会发生变化，间接对其活性产生影响。如果目的蛋白是某蛋白质修饰底物，则要考虑鉴定催化该底物修饰或去催化该底物修饰的酶，如激酶（kinase）、乙酰转移酶（acetyltransferase, HAT）、去乙酰化转移酶（deacetylase, HDAC）、甲基转移酶（methyltransferase, KMT）、去甲基转移酶（demethylase, KDMT）、E3连接酶（E3 ligase）等；此外，在表观遗传学领域，对于某蛋白质修饰的特异底物，在生化分析层面上还要考虑去鉴定与该修饰特异性结合的相互作用蛋白（称之为“阅读器”，reader），如与乙酰化基团特异结合的含有bromodomain的蛋白、与赖氨酸甲基化基团特异结合的含有chromodomain的蛋白等。这些特异的催化酶、去催化酶、“阅读器”蛋白的筛选与鉴定一方面可以根据文献报道来初步确定，然后进一步生化验证；另一方面也可利用免疫沉淀（Immunoprecipitation，IP）、pull down等手段结合质谱分析寻找鉴定，为进一步的机制研究提供线索。此外，还可以利用结构解析或者软件预测等方式研究修饰基团对蛋白构象的影响。  
 3）细胞生物学层面：利用免疫荧光（Immunoflurescence，IF）、FRET等荧光标记手段对目标分子的亚细胞定位和实时动态变化进行分析，为功能机制的研究和蛋白质相互作用提供依据；敲除目的蛋白或引入目的蛋白的过表达突变体，在表型变化的层面上，观察目的蛋白表达水平改变或修饰水平的改变对细胞周期、细胞增殖、迁移、极性、凋亡以及细胞间通讯等过程的影响。  
 4）模式生物实验层面：如果你筛选出的目的蛋白与肿瘤生成密切相关，则可以考虑通过构建基因敲除小鼠、肿瘤异种移植（Xenograft Model）模型，结合表型分析和生化功能研究，在动物模型中深入探索目的蛋白及相关信号通路在生理病理过程中的意义。在植物学领域，如可采用拟南芥为模式生物，通过构建目的蛋白的突变株，结合表型分析和生化功能研究，深入探索目的蛋白生物学功能。  
 5）其他：建议结合各领域本身的特定研究方法，开展针对性验证实验。  
 基于质谱数据的后续功能性研究，景杰生物选取了部分优秀的文章，其研究思路可供您参考（见报告附件）。  
 **在有关蛋白质表达或蛋白质修饰定量改变验证、目的蛋白功能验证方面，景杰生物能够为您提供高质量的目录抗体或根据您的要求开展蛋白质修饰抗体的定制服务。景杰生物开发有世界上种类最全的修饰泛抗体和组蛋白位点特异性抗体用于实验验证。对于没有现成抗体的蛋白尤其是新型修饰位点来说，我们提供高质量的特异性抗体的定制服务，可以为后续的验证和功能学研究提供帮助。**

# References

1. Li, L. et al. SIRT7 is a histone desuccinylase that functionally links to chromatin compaction and genome stability. Nat Commun 7, 12235, doi:10.1038/ncomms12235 (2016).
2. Du, Y. et al. Lysine malonylation is elevated in type 2 diabetic mouse models and enriched in metabolic associated proteins. Mol Cell Proteomics 14, 227-236, doi:10.1074/mcp.M114.041947 (2015).
3. Song, L., Wang, G., Malhotra, A., Deutscher, M. P. & Liang, W. Reversible acetylation on Lys501 regulates the activity of RNase II. Nucleic Acids Res 44, 1979-1988, doi:10.1093/nar/gkw053 (2016).
4. Du, Y. et al. Lysine malonylation is elevated in type 2 diabetic mouse models and enriched in metabolic associated proteins. Molecular & Cellular Proteomics Mcp 14, 227 (2014).
5. Zhao, D. et al. Lysine-5 acetylation negatively regulates lactate dehydrogenase A and is decreased in pancreatic cancer. Cancer Cell 23, 464-476, doi:10.1016/j.ccr.2013.02.005 (2013).
6. Xiangyun, Y. et al. Desuccinylation of pyruvate kinase M2 by SIRT5 contributes to antioxidant response and tumor growth. Oncotarget 8, 6984-6993, doi:10.18632/oncotarget.14346 (2017).

# 3 材料和方法

## 3.1 实验方法

### 3.1.1 材料与试剂

##### 表4. 样品制备所需材料和试剂

### 3.1.2. 蛋白提取

样品从-80℃取出，待其完全化冻后，4℃，750 g 离心 5 min，将上清和细胞沉淀分开提取，向上清液中分别加入 4 倍体积预冷丙酮，涡旋混匀，于-20℃沉淀 2 h 4℃， 5500 g 离心 5 min，弃上清，沉淀再用预冷的丙酮洗涤三次，沉淀用 8 M 尿素复溶。 向细胞沉淀中分别加入 4 倍体积裂解缓冲液（8 M 尿素， 1%蛋白酶抑制剂， 3 μM TSA， 50 mM NAM），超声裂解。4℃，12000 g 离心 10 min，去除细胞碎片，上清液转移至新的离心管，与尿素复溶的上清蛋白合并，利用 BCA 试剂盒进行蛋白浓度测定。

### 3.1.3. 胰酶酶解

各样品蛋白取等量进行酶解，加入适量标准蛋白，用裂解液将体积调整至一致。缓慢加入终浓度20% TCA，涡旋混匀，4℃沉淀2h。4500g，离心5min，弃上清，用预冷的丙酮洗涤沉淀2-3次。晾干沉淀后加入终浓度200 mM的TEAB，超声打散沉淀，以1:50的比例（蛋白酶：蛋白，m/m）加入胰蛋白酶，酶解过夜。加入二硫苏糖醇（DTT）使其终浓度为5 mM，56℃还原30 min。之后加入碘乙酰胺（IAA）使其终浓度为11 mM，室温避光孵育15 min。

### 3.1.4. TMT标记

胰酶酶解的肽段用Strata X C18（Phenomenex）除盐后真空冷冻干燥。以0.5 M TEAB溶解肽段，根据TMT试剂盒操作说明标记肽段。简单的操作如下：标记试剂解冻后用乙腈溶解，与肽段混合后室温孵育2 h，标记后的肽段混合后除盐，真空冷冻干燥。

##### 表5. 样品标记信息

### 3.1.5 HPLC分级

肽段用高pH反向HPLC分级，色谱柱为Agilent 300Extend C18(5 μm粒径，4.6 mm 内径，250 mm长)。操作如下：肽段分级梯度为8%-32%乙腈、pH 9，60 min时间分离60个组分，随后肽段合并为4个组分，合并后的组分经真空冷冻干燥后进行后续操作。

### 3.1.6 修饰富集

将肽段溶解在IP缓冲溶液中（100 mM NaCl, 1 mM EDTA, 50 mM Tris-HCl, 0.5% NP-40, pH 8.0)，转移上清液至提前洗涤好的乙酰化树脂中（抗体树脂货号PTM-104，来源于杭州景杰生物科技有限公司，PTM Bio），放置于4 ℃ 环境的旋转摇床上，温和摇晃并过夜孵育。孵育结束后依次使用IP缓冲溶液洗涤树脂4次，去离子水洗涤两次。最后使用0.1%三氟乙酸洗脱液，将树脂结合的肽段洗脱下来，共洗脱三次，收集洗脱液并真空冷冻抽干。抽干后按照C18 ZipTips说明书除盐，真空冷冻抽干后供液质联用分析。

### 3.1.7 液相色谱-质谱联用分析

肽段用液相色谱流动相A相溶解后使用EASY-nLC 1000超高效液相系统进行分离。流动相A为含0.1%甲酸和2%乙腈的水溶液；流动相B为含0.1%甲酸和90%乙腈的水溶液。 液相梯度设置：0-26 min，8%~25%B；26-34 min，25%~35%B；34-37 min，35%~80%B；37-40 min，80%B， 流速维持在500 nL/min。   
 肽段经由超高效液相系统分离后被注入NSI离子源中进行电离然后进Q Exactive质谱进行分析。 离子源电压设置为2.0 kV，肽段母离子及其二级碎片都使用高分辨的Orbitrap进行检测和分析。一级质谱扫描范围设置为350-1800 m/z， 扫描分辨率设置为70,000；二级质谱扫描范围则固定起点为100 m/z，二级扫描分辨率设置为17,500。数据采集模式使用数据依赖型扫描（DDA）程序， 即在一级扫描后选择信号强度最高的前15肽段母离子依次进入HCD碰撞池使用28%的碎裂能量进行碎裂，同样依次进行二级质谱分析。为了提高质谱的有效利用率， 自动增益控制（AGC）设置为5E4，信号阈值设置为10000 ions/s，最大注入时间设置为200 ms， 串联质谱扫描的动态排除时间设置为15秒避免母离子的重复扫描。

### 3.1.8 数据库搜索

二级质谱数据使用Maxquant (v1.5.2.8)进行检索。检索参数设置：数据库为Homo\_sapiens\_9606\_SP\_20191115（20380条序列），添加了反库以计算随机匹配造成的假阳性率（FDR），并且在数据库中加入了常见的污染库，用于消除鉴定结果中污染蛋白的影响；酶切方式设置为Trypsin/P；漏切位点数设为2；肽段最小长度设置为7个氨基酸残基；肽段最大修饰数设为5；First search和Main search的一级母离子质量误差容忍度分别设为20 ppm和5 ppm，二级碎片离子的质量误差容忍度为0.02 Da。将半胱氨酸烷基化设置为固定修饰，可变修饰为甲硫氨酸的氧化,蛋白N端的乙酰化,脱酰胺化(NQ),赖氨酸的乙酰化。定量方法设置为TMT-6plex，蛋白鉴定、PSM鉴定的FDR都设置为1%。

### 3.1.9 质谱质控检测

如下图可见，大部分肽段分布在7-20个氨基酸，符合基于trypsin酶解和HCD碎裂方式的一般规律。其中小于5个氨基酸的肽段由于产生的碎片离子过少，不能产生有效的序列鉴定。大于20个氨基酸的肽段由于质量和电荷数较高，不适合HCD的碎裂方式。质谱鉴定到的肽段长度的分布符合质控要求。

#### 图19 质谱鉴定到肽段的长度分布

#### 图20 蛋白对应修饰位点量

#### 图21 质谱数据的质量精度分布

质控数据请参见：“../2-Basic\_analysis/QC/”。

## 3.2 生物信息学分析方法

### 3.2.1 分析使用软件

| 分析 | 软件/方法 | 版本/网址 |
| --- | --- | --- |
| 质谱数据解析 | MaxQuant | v.1.5.2.8 http://www.maxquant.org/ |
| Motif分析 | MoMo | V5.0.2 http://meme-suite.org/tools/momo |
| GO注释 | InterProScan | v.5.14-53.0 http://www.ebi.ac.uk/interpro/ |
| Domain注释 | InterProScan | v.5.14-53.0 http://www.ebi.ac.uk/interpro/ |
| KEGG注释 | KAAS | v.2.0 http://www.genome.jp/kaas-bin/kaas\_main |
|  | KEGG Mapper | V2.5 http://www.kegg.jp/kegg/mapper.html |
| 亚细胞定位 | Wolfpsort | v.0.2 http://www.genscript.com/psort/wolf\_psort.html |
|  | CELLO | v.2.5 http://cello.life.nctu.edu.tw/ |
| 富集分析 | Perl module | v.1.31 https://metacpan.org/pod/Text::NSP::Measures::2D::Fisher |
| 聚类热图 | R Package pheatmap | v.2.0.3 https://cran.r-project.org/web/packages/cluster/ |
| 蛋白互作 | Blast | v.2.2.26 http://blast.ncbi.nlm.nih.gov/Blast.cgi |
|  | R package networkD3 | v.0.4 https://cran.r-project.org/web/packages/networkD3/ |

### 3.2.2 蛋白注释方法

**Gene Ontology分析**   
 Gene Ontology分析，或GO分析，是一种能够将基因与基因产物（如蛋白质）的各项信息有机的联系在一起进而提供统计学信息的生物信息学分析方法。在蛋白质组学项目中，GO主要有下列用途：  
 1. 作为蛋白及基因的各项信息的数据库；  
 2. 提供蛋白及基因的各种信息，并按照信息对蛋白及基因进行分类；   
 3. 作为一个工具，为项目中的所有蛋白提供最全面的信息注释和分类服务。   
 Gene Ontology分析主要包括三个方面：  
 1. 细胞组成：细胞组成是指细胞的特定成分，在GO系统中它应当是细胞中较大组分的组成元件。比如解剖学上的某些细胞结构（糙面内质网、细胞核等），或一系列基因产物，如一些复杂成分的基础结构（核糖体，蛋白二聚体等）。

**KEGG通路注释**   
 KEGG（Kyoto Encyclopedia of Genes and Genomes）能够将当前已知的蛋白相互作用网络信息，比如通路及相关复合体（“Pathway”数据库）、基因及基因产物（“Gene”数据库）、生物内复合物及相关反应（“compound and reaction”数据库）等信息进行整合。KEGG的通路主要包括：代谢、遗传信息处理、环境信息相关进程、细胞生理进程、药物研究等。 我们使用KEGG通路数据库对蛋白通路进行注释：首先，使用KEGG在线服务工具KAAS对提交的蛋白进行注释，之后通过KEGG mapper将注释过的蛋白匹配入数据库中相应的通路中。  
 **亚细胞定位**  
 真核生物组织细胞中的蛋白，依据与其结合的膜结构的差异，被详尽的定位到细胞内各种元件上。真核细胞主要的亚细胞定位包括：胞外、细胞质、细胞核、线粒体、高尔基体、内质网、过氧化物酶体、液泡、细胞骨架、核质、核基质以及核糖体等。  
 基于此，我们使用预测亚细胞定位的软件wolfpsort对所提交的蛋白进行亚细胞定位注释。针对原核生物我们使用CELLO软件对其蛋白进行亚细胞结构预测分析。

### 3.2.3 修饰位点基序分析

软件MoMo，motif-x算法被用于分析修饰位点的基序特征。其中所有鉴定到的修饰位点上下游各10个（磷酸化修饰为上下游各6个）氨基酸组成的肽段序列作为分析对象；分析比较背景为物种中所有潜在发生修饰位点上下游各10个（磷酸化修饰为上下游各6个）氨基酸组成的肽段序列。当某个特性序列形式的肽段数量大于20，且统计学检验P值小于0.000001时，认为该特征序列形式为修饰肽段的一种基序。

### 3.2.4 蛋白质功能富集

**GO富集分析**   
 蛋白的GO注释被分为3个大类：生物进程、细胞组成、分子功能。费歇尔精确双端检验方法(Fisher’s exact test)被用于检验差异修饰蛋白在以鉴定到的蛋白为背景，GO富集检验P-value值小于0.05被认为是显著的。   
 **通路富集分析**   
 Kyoto Encyclopedia of Genes and Genomes（KEGG）数据库被用于通路的富集分析。费歇尔精确双端检验方法被用于检验差异修饰蛋白在以鉴定到的蛋白为背景，通路富集检验P-value值小于0.05被认为是显著的。最后根据KEGG网站通路层级分类方法将这些通路进行分类。   
 **蛋白结构域富集分析**   
 InterPro（对蛋白序列的家族分类、结构域和特殊位点的预测等功能分析提供资源）数据库用于分析差异修饰蛋白的功能结构域的富集情况。费歇尔精确双端检验方法被用于检验差异修饰蛋白在以鉴定到的蛋白为背景，结构域单元富集检验P-value值小于0.05被认为是显著的。

### 3.2.5 基于蛋白功能富集的聚类分析

基于不同分组的差异修饰蛋白（或者不同差异倍数的差异修饰蛋白）功能富集的聚类分析用于研究其在特定功能（GO，KEGG通路，蛋白结构域等）上存在的潜在联系和差异。我们首先收集所用蛋白分组富集到的功能分类信息和对应的富集P-value值，然后筛选出至少在一个蛋白分组中为显著富集（P-value<0.05）的功能分类。筛选得到的P-value数据矩阵首先经过以-log10的对数变换，然后将变换后的数据矩阵对各功能分类运用Z变换。最后将Z变换后得到的数据集使用分层聚类（欧式距离，平均连接聚类）方法做单边聚类分析。聚类关系使用R语言包gplots中的函数heatmap.2绘制出的热图进行可视化展示。

### 3.2.6 蛋白互作网络分析

将不同比较组中筛选得到的差异修饰蛋白数据库编号或蛋白序列，通过与STRING（v.11.0）蛋白网络互作网络数据库比对后，按照confidence score >0.7 (high confidence)提取得到差异蛋白互作关系。然后通过R package “networkD3”工具对差异蛋白互作网络进行可视化展示。

# 4 英文版实验方法模板（供参考）

以下实验方法仅供您英文文章撰写时参考，所描述的实验方法包括但不限于您的项目，具体实验步骤以中文版项目报告为准。请根据您的样品和项目类型选择相应部分，并作适当修改。

## 4.1 Experimental Procedures

### 4.1.1 Protein Extraction (optional)

**For cell samples:**   
 Sample was sonicated three times on ice using a high intensity ultrasonic processor (Scientz) in lysis buffer (8 M urea, 1% Protease Inhibitor Cocktail). (Note: For PTM experiments, inhibitors were also added to the lysis buffer, e.g. 3 μM TSA and 50 mM NAM for acetylation.) The remaining debris was removed by centrifugation at 12,000 g at 4 °C for 10 min. Finally, the supernatant was collected and the protein concentration was determined with BCA kit according to the manufacturer’s instructions.  
 **For tissue samples:**   
 The sample was grinded by liquid nitrogen into cell powder and then transferred to a 5-mL centrifuge tube. After that, four volumes of lysis buffer (8 M urea, 1% Protease Inhibitor Cocktail) was added to the cell powder, followed by sonication three times on ice using a high intensity ultrasonic processor (Scientz). (Note: For PTM experiments, inhibitors were also added to the lysis buffer, e.g. 3 μM TSA and 50 mM NAM for acetylation.) The remaining debris was removed by centrifugation at 12,000 g at 4 °C for 10 min. Finally, the supernatant was collected and the protein concentration was determined with BCA kit according to the manufacturer’s instructions.   
 **For plant and fungus samples:**   
 The sample was grinded by liquid nitrogen into cell powder and then transferred to a 5-mL centrifuge tube. After that, four volumes of lysis buffer (8 M urea, 1% Triton-100, 10 mM dithiothreitol, and 1% Protease Inhibitor Cocktail) was added to the cell powder, followed by sonication three times on ice using a high intensity ultrasonic processor (Scientz). (Note: For PTM experiments, inhibitors were also added to the lysis buffer, e.g. 3 μM TSA and 50 mM NAM for acetylation.) The remaining debris was removed by centrifugation at 20,000 g at 4 °C for 10 min. Finally, the protein was precipitated with cold 20% TCA for 2 h at -20 °C. After centrifugation at 12,000 g 4 °C for 10 min, the supernatant was discarded. The remaining precipitate was washed with cold acetone for three times. The protein was redissolved in 8 M urea and the protein concentration was determined with BCA kit according to the manufacturer’s instructions.  
 **For plant and fungus samples (Phenol):**  
 Sample was first grinded by liquid nitrogen, then the powder was transferred to 5 mL centrifuge tube and sonicated three times on ice using a high intensity ultrasonic processor (Scientz) in lysis buffer (including 1% TritonX-100, 10 mM dithiothreitol, and 1% Protease Inhibitor Cocktail, 50 μM PR-619，3 μM TSA，50 mM NAM and 2 mM EDTA). An equal volume of Tris-saturated phenol (pH 8.0) was added; then, the mixture was further vortexed for 5 min. After centrifugation (4 °C, 10 min, 5 000g), the upper phenol phase was transferred to a new centrifuge tube. Proteins were precipitated by adding at least four volumes of ammonium sulfate-saturated methanol and incubated at -20 °C for at least 6 h. After centrifugation at 4 °C for 10 min, the supernatant was discarded. The remaining precipitate was washed with ice-cold methanol once, followed by ice-cold acetone for three times. The protein was redissolved in 8 M urea and the protein concentration was determined with BCA kit according to the manufacturer’s instructions.  
 **Serum samples:**  
 Firstly, the cellular debris of serum sample was removed by centrifugation at 12,000 g at 4 °C for 10 min. Then, the supernatant was transferred to a new centrifuge tube. The top 12 high abundance proteins were removed by Pierce™ Top 12 Abundant Protein Depletion Spin Columns Kit (Thermo Fisher). Finally, the protein concentration was determined with BCA kit according to the manufacturer’s instructions.

### 4.1.2 Trypsin Digestion

For digestion, the protein solution was reduced with 5 mM dithiothreitol for 30 min at 56 °C and alkylated with 11 mM iodoacetamide for 15 min at room temperature in darkness. The protein sample was then diluted by adding 100 mM TEAB to urea concentration less than 2M. Finally, trypsin was added at 1:50 trypsin-to-protein mass ratio for the first digestion overnight and 1:100 trypsin-to-protein mass ratio for a second 4 h-digestion.

### 4.1.3 TMT/iTRAQ Labeling (optional)

After trypsin digestion, peptide was desalted by Strata X C18 SPE column (Phenomenex) and vacuum-dried. Peptide was reconstituted in 0.5 M TEAB and processed according to the manufacturer’s protocol for TMT kit/iTRAQ kit. Briefly, one unit of TMT/iTRAQ reagent were thawed and reconstituted in acetonitrile. The peptide mixtures were then incubated for 2 h at room temperature and pooled, desalted and dried by vacuum centrifugation.

### 4.1.4 HPLC Fractionation (optional)

The tryptic peptides were fractionated into fractions by high pH reverse-phase HPLC using Thermo Betasil C18 column (5 μm particles, 10 mm ID, 250 mm length). Briefly, peptides were first separated with a gradient of 8% to 32% acetonitrile (pH 9.0) over 60 min into 60 fractions. Then, the peptides were combined into 6 fractions and dried by vacuum centrifuging.

### 4.1.5 Affinity Enrichment (optional)

Pan antibody-based PTM enrichment:   
 To enrich modified peptides, tryptic peptides dissolved in NETN buffer (100 mM NaCl, 1 mM EDTA, 50 mM Tris-HCl, 0.5% NP-40, pH 8.0) were incubated with pre-washed antibody beads (Lot number 001, PTM Bio) at 4°C overnight with gentle shaking. Then the beads were washed four times with NETN buffer and twice with H2O. The bound peptides were eluted from the beads with 0.1% trifluoroacetic acid. Finally, the eluted fractions were combined and vacuum-dried. For LC-MS/MS analysis, the resulting peptides were desalted with C18 ZipTips (Millipore) according to the manufacturer’s instructions. Bio-material-based PTM enrichment (for phosphorylation):   
 Peptide mixtures were first incubated with IMAC microspheres suspension with vibration in loading buffer (50% acetonitrile/6% trifluoroacetic acid). The IMAC microspheres with enriched phosphopeptides were collected by centrifugation, and the supernatant was removed. To remove nonspecifically adsorbed peptides, the IMAC microspheres were washed with 50% acetonitrile/6% trifluoroacetic acid and 30% acetonitrile/0.1% trifluoroacetic acid, sequentially. To elute the enriched phosphopeptides from the IMAC microspheres, elution buffer containing 10% NH4OH was added and the enriched phosphopeptides were eluted with vibration. The supernatant containing phosphopeptides was collected and lyophilized for LC-MS/MS analysis.

### 4.1.6 LC-MS/MS Analysis

The tryptic peptides were dissolved in 0.1% formic acid (solvent A), directly loaded onto a home-made reversed-phase analytical column (15-cm length, 75 μm i.d.). The gradient was comprised of an increase from 6% to 23% solvent B (0.1% formic acid in 98% acetonitrile) over 26 min, 23% to 35% in 8 min and climbing to 80% in 3 min then holding at 80% for the last 3 min, all at a constant flow rate of 400 nL/min on an EASY-nLC 1000 UPLC system.   
 The peptides were subjected to NSI source followed by tandem mass spectrometry (MS/MS) in Q ExactiveTM Plus (Thermo) coupled online to the UPLC. The electrospray voltage applied was 2.0 kV. The m/z scan range was 350 to 1800 for full scan, and intact peptides were detected in the Orbitrap at a resolution of 70,000. Peptides were then selected for MS/MS using NCE setting as 28 and the fragments were detected in the Orbitrap at a resolution of 17,500. A data-dependent procedure that alternated between one MS scan followed by 20 MS/MS scans with 15.0s dynamic exclusion. Automatic gain control (AGC) was set at 5E4. Fixed first mass was set as 100 m/z.

### 4.1.7 Database Search

The resulting MS/MS data were processed using Maxquant search engine (v.1.5.2.8). Tandem mass spectra were searched against human uniprot database concatenated with reverse decoy database. Trypsin/P was specified as cleavage enzyme allowing up to 4 missing cleavages. The mass tolerance for precursor ions was set as 20 ppm in First search and 5 ppm in Main search, and the mass tolerance for fragment ions was set as 0.02 Da. Carbamidomethyl on Cys was specified as fixed modification and Acetylation modification and oxidation on Met were specified as variable modifications. FDR was adjusted to < 1% and minimum score for modified peptides was set > 40.

## 4.2. Bioinformatics Methods

### 4.2.1 Annotation Methods

**GO Annotation**   
 The Gene Ontology, or GO, is a major bioinformatics initiative to unify the representation of gene and gene product attributes across all species. More specifically, the project aims to:   
 1. Maintain and develop its controlled vocabulary of gene and gene product attributes;  
 2. Annotate genes and gene products, and assimilate and disseminate annotation data;  
 3. Provide tools for easy access to all aspects of the data provided by the project.  
 The ontology covers three domains:   
 1. Cellular component: A cellular component is just that, a component of a cell, but with the proviso that it is part of some larger object; this may be an anatomical structure (e.g. rough endoplasmic reticulum or nucleus) or a gene product group (e.g. ribosome, proteasome or a protein dimer).  
 2. Molecular function: Molecular function describes activities, such as catalytic or binding activities, that occur at the molecular level. GO molecular function terms represent activities rather than the entities (molecules or complexes) that perform the actions, and do not specify where or when, or in what context, the action takes place.  
 3. Biological process: A biological process is series of events accomplished by one or more ordered assemblies of molecular functions. It can be difficult to distinguish between a biological process and a molecular function, but the general rule is that a process must have more than one distinct steps.  
 Gene Ontology (GO) annotation proteome was derived from the UniProt-GOA database ( http://www.ebi.ac.uk/GOA/). Firstly, Converting identified protein ID to UniProt ID and then mapping to GO IDs by protein ID. If some identified proteins were not annotated by UniProt-GOA database, the InterProScan soft would be used to annotated protein’s GO functional based on protein sequence alignment method. Then proteins were classified by Gene Ontology annotation based on three categories: biological process, cellular component and molecular function.   
 **Domain Annotation**   
 A protein domain is a conserved part of a given protein sequence and structure that can evolve, function and exist independently of the rest of the protein chain. Each domain forms a compact three-dimensional structure and often can be independently stable and folded. Many proteins consist of several structural domains. One domain may appear in a variety of differentially modified proteins. Molecular evolution uses domains as building blocks and these may be recombined in different arrangements to create proteins with different functions. Domains vary in length from between about 25 amino acids up to 500 amino acids in length. The shortest domains such as zinc fingers are stabilized by metal ions or disulfide bridges. Domains often form functional units, such as the calcium-binding EF hand domain of calmodulin. Because they are independently stable, domains can be “swapped” by genetic engineering between one protein and another to make chimeric proteins.   
 Identified proteins domain functional description were annotated by InterProScan (a sequence analysis application) based on protein sequence alignment method, and the InterPro domain database was used. InterPro (http://www.ebi.ac.uk/interpro/) is a database that integrates diverse information about protein families, domains and functional sites, and makes it freely available to the public via Web-based interfaces and services. Central to the database are diagnostic models, known as signatures, against which protein sequences can be searched to determine their potential function. InterPro has utility in the large-scale analysis of whole genomes and meta-genomes, as well as in characterizing individual protein sequences.  
 **KEGG Pathway Annotation**   
 KEGG connects known information on molecular interaction networks, such as pathways and complexes (the “Pathway” database), information about genes and proteins generated by genome projects (including the gene database) and information about biochemical compounds and reactions (including compound and reaction databases). These databases are different networks, known as the “protein network”, and the “chemical universe” respectively. There are efforts in progress to add to the knowledge of KEGG, including information regarding ortholog clusters in the KEGG Orthology database. KEGG Pathways mainly including: Metabolism, Genetic Information Processing, Environmental Information Processing, Cellular Processes, Rat Diseases, Drug development. Kyoto Encyclopedia of Genes and Genomes (KEGG) database was used to annotate protein pathway. Firstly, using KEGG online service tools KAAS to annotated protein’s KEGG database description. Then mapping the annotation result on the KEGG pathway database using KEGG online service tools KEGG mapper.   
 **Subcellular Localization**   
 The cells of eukaryotic organisms are elaborately subdivided into functionally distinct membrane bound compartments. Some major constituents of eukaryotic cells are: extracellular space, cytoplasm, nucleus, mitochondria, Golgi apparatus, endoplasmic reticulum (ER), peroxisome, vacuoles, cytoskeleton, nucleoplasm, nucleolus, nuclear matrix and ribosomes.   
 Bacteria also have subcellular localizations that can be separated when the cell is fractionated. The most common localizations referred to include the cytoplasm, the cytoplasmic membrane (also referred to as the inner membrane in Gram-negative bacteria), the cell wall (which is usually thicker in Gram-positive bacteria) and the extracellular environment. Most Gram-negative bacteria also contain an outer membrane and periplasmic space. Unlike eukaryotes, most bacteria contain no membrane-bound organelles, however there are some exceptions.   
 There, we used wolfpsort a subcellular localization predication soft to predict subcellular localization. Wolfpsort is an updated version of PSORT/PSORT II for the prediction of eukaryotic sequences. Special for protokaryon species, Subcellular localization prediction soft CELLO was used.

### 4.2.2 Motif Analysis

Soft MoMo (motif-x algorithm) was used to analysis the model of sequences constituted with amino acids in specific positions of modify-21-mers (10 amino acids upstream and downstream of the site， but phosphorylation with modify-13-mers that 6 amino acids upstream and downstream of the site) in all protein sequences. And all the database protein sequences were used as background database parameter. Minimum number of occurrences was set to 20. Emulate original motif-x was ticked, and other parameters with default.

### 4.2.3 Functional Enrichment

**Enrichment of Gene Ontology analysis**   
 Proteins were classified by GO annotation into three categories: biological process, cellular compartment and molecular function. For each category, a two-tailed Fisher’s exact test was employed to test the enrichment of the differentially modified protein against all identified proteins. The GO with a corrected p-value < 0.05 is considered significant.   
 **Enrichment of pathway analysis**  
 Encyclopedia of Genes and Genomes (KEGG) database was used to identify enriched pathways by a two-tailed Fisher’s exact test to test the enrichment of the differentially modified protein against all identified proteins. The pathway with a corrected p-value < 0.05 was considered significant. These pathways were classified into hierarchical categories according to the KEGG website.   
 **Enrichment of protein domain analysis**   
 For each category proteins, InterPro (a resource that provides functional analysis of protein sequences by classifying them into families and predicting the presence of domains and important sites) database was researched and a two-tailed Fisher’s exact test was employed to test the enrichment of the differentially modified protein against all identified proteins. Protein domains with a corrected p-value < 0.05 were considered significant.

### 4.2.4 Enrichment-based Clustering

For further hierarchical clustering based on differentially modified protein functional classification (such as: GO, Domain, Pathway, Complex). We first collated all the categories obtained after enrichment along with their P values, and then filtered for those categories which were at least enriched in one of the clusters with P value <0.05. This filtered P value matrix was transformed by the function x = −log10 (P value). Finally these x values were z-transformed for each functional category. These z scores were then clustered by one-way hierarchical clustering (Euclidean distance, average linkage clustering) in Genesis. Cluster membership were visualized by a heat map using the “heatmap.2” function from the “gplots” R-package.

### 4.2.5 Protein-protein Interaction Network

All differentially expressed modified protein database accession or sequence were searched against the STRING database version 10.1 for protein-protein interactions. Only interactions between the proteins belonging to the searched data set were selected, thereby excluding external candidates. STRING defines a metric called “confidence score” to define interaction confidence; we fetched all interactions that had a confidence score ≥ 0.7 (high confidence). Interaction network form STRING was visualized in R package “networkD3”.
